# Supplementary material for: Blood metabolomic profiling reveals new targets in the management of psychological symptoms associated with severe alcohol use disorder
Source: eLife. 2024 Nov 29;13:RP96937. doi: 10.7554/eLife.96937 (PMC11606602; doi:10.7554/eLife.96937)
Supplement: Supplementary file 1. [file elife-96937-supp1.docx]

**Supplementary File 1.** biological features of healthy controls and background characteristics of the selected subjects from the TSDS cohort.

**Table S1.** biological features of healthy controls

|  | ALCOHOLBIS cohort | GUT2BRAIN cohort | All | p value |
| --- | --- | --- | --- | --- |
| Number of subjects | 19 | 13 | 32 |  |
| Age | 43 ± 8 | 47 ± 12 | 45 ± 10 | 0.35 |
| Gender  Men, n (%)  Women, n (%) | 8 (42%)  11 (58%) | 8 (61.5%)  5 (8.5%) | 16 (50%)  16 (50%) | 0.28^$^ |
| Smoking status  Active smoker (%)  Non-smoker (%) | 1 (5%)  18 (95%) | 3 (23%)  10 (77%) | 4 (12.5%)  28 (87.5%) | 0.14^$^ |
| BMI | 23.6 ± 4.7 | 23.9 ± 3.2 | 23.7 ± 4.1 | 0.20 |
| AUDIT |  | 3.3 ± 2.2 |  |  |

Results are means ± standard deviations. Independent t-tests to compare ALCOHOLBIS *versus* GUT2BRAIN cohorts; ^$^chi-square test for categorical variables

**Table S2.** Background characteristics of the selected subjects from the TSDS cohort

|  | Control  (n = 100) | | Alcohol  (n = 97) | | p value |
| --- | --- | --- | --- | --- | --- |
| PMI (days, mean ±SD) | 5.8 | ± 3.0 | 5.5 | ± 2.1 | 0.3081^a^ |
| Age (years, mean ±SD) | 57 | ± 13 | 57 | ± 10 | 0.8320^a^ |
| Sex (n females, %) | 12 | (12 %) | 22 | (22%) | 0.0738^b^ |
| BMI (mean ±SD) | 30.9 | ± 8.0 | 28.0 | ± 7.2 | 0.0090^a^ |
| Brain (grams, mean ±SD) | 1471 | ± 152 | 1448 | ± 148 | 0.2965^a^ |
| Smoking (n, %)^c^ | 32 | (55 %) | 37 | (67 %) | 0.1873^b^ |
| CSF Alcohol (‰, mean ±SD)^d^ | 0.13 | ± 0.37 | 0.94 | ± 0.67 | <0.0001^a^ |
| Urine Alcohol (‰, mean ±SD)^e^ | 0.28 | ± 0.54 | 1.12 | ± 0.56 | <0.0001^a^ |

^a^Welch’s t-test; ^b^ χ2 test; ^c^smoking status known only from 113 subjects (58 controls, 55 heavy alcohol users); ^d^CSF alcohol concentration only from 106 subjects (50 controls, 56 heavy alcohol users); ^e^Urine alcohol concentration only from 38 subjects (18 controls, 20 heavy alcohol users); BMI, body mass index; CSF, cerebrospinal fluid; PMI, post-mortem interval
