# Supplementary material for: Blood metabolomic profiling reveals new targets in the management of psychological symptoms associated with severe alcohol use disorder
Source: eLife. 2024 Nov 29;13:RP96937. doi: 10.7554/eLife.96937 (PMC11606602; doi:10.7554/eLife.96937)

# **Supplementary File 3.** EICs, retention times and reference spectra for level 1 identifications

1-Methylhistidine

RT std: 6.21min, RT experimental: 6.42min, RT Δ 0.21min


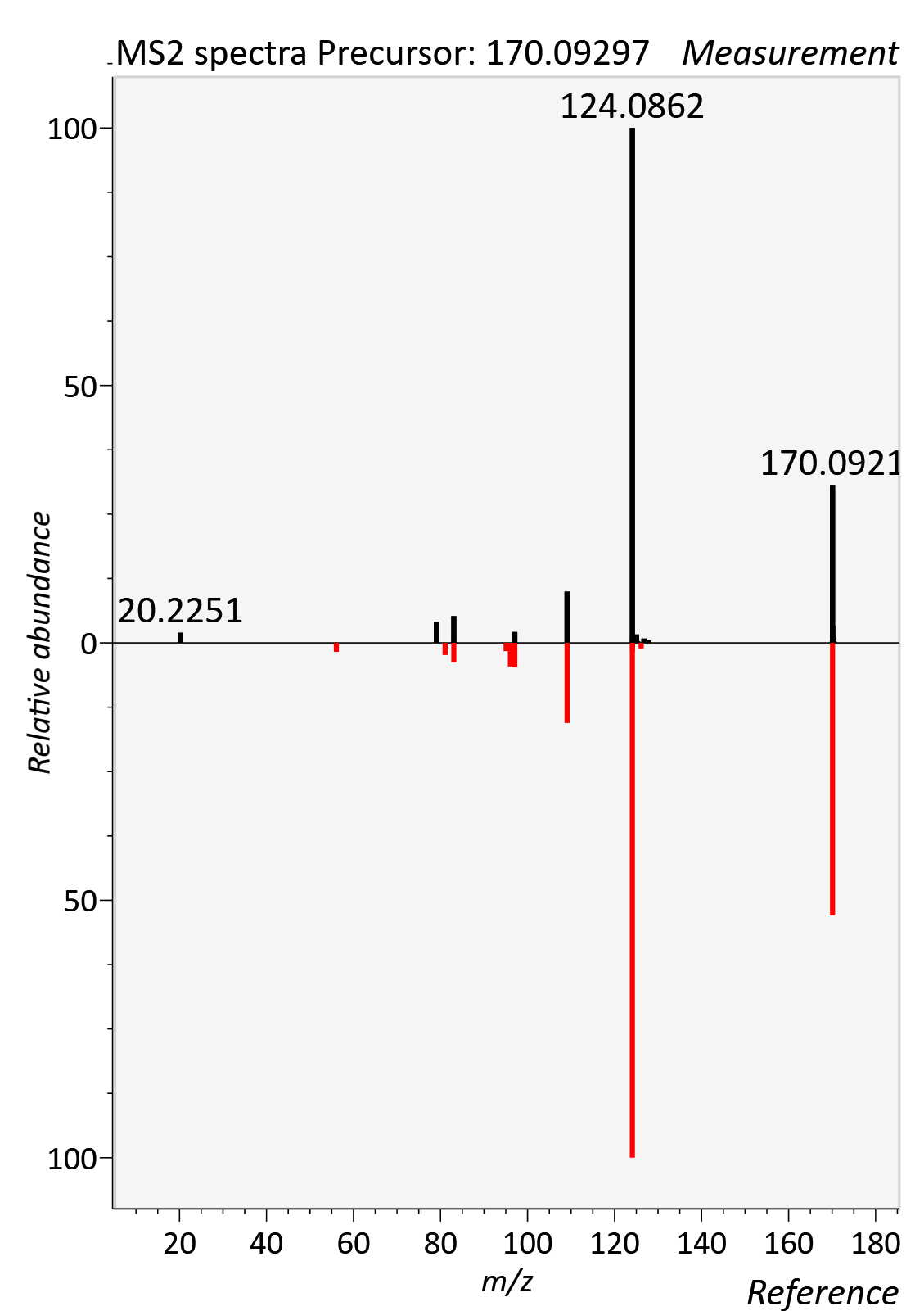


1-Methylnicotincamide

RT std: 2.18min, RT experimental: 2.39min, RT Δ 0.21min


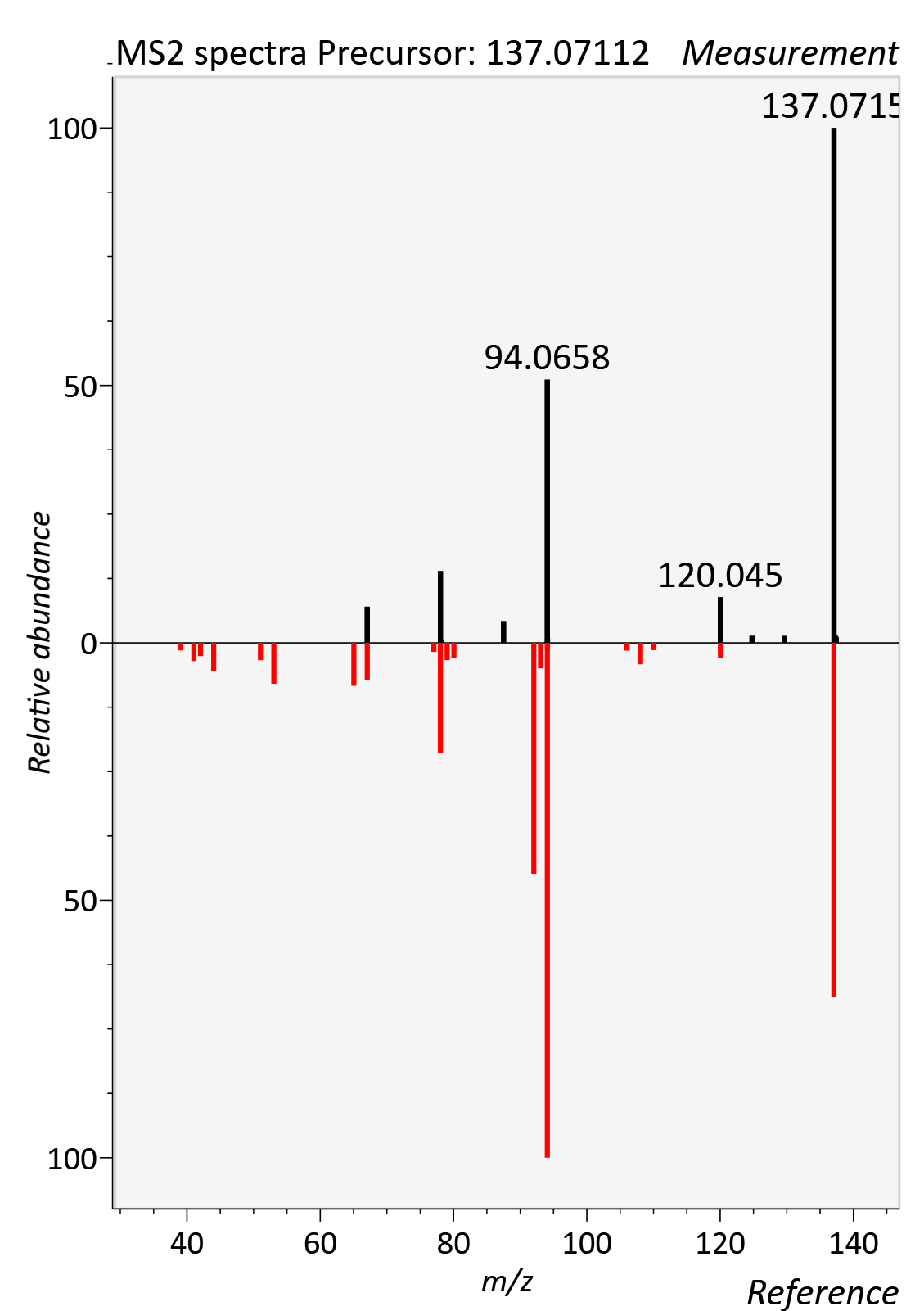


3-Indoleacetic acid

RT std: 5.00min, RT experimental: 5.28min, RT Δ 0.28min


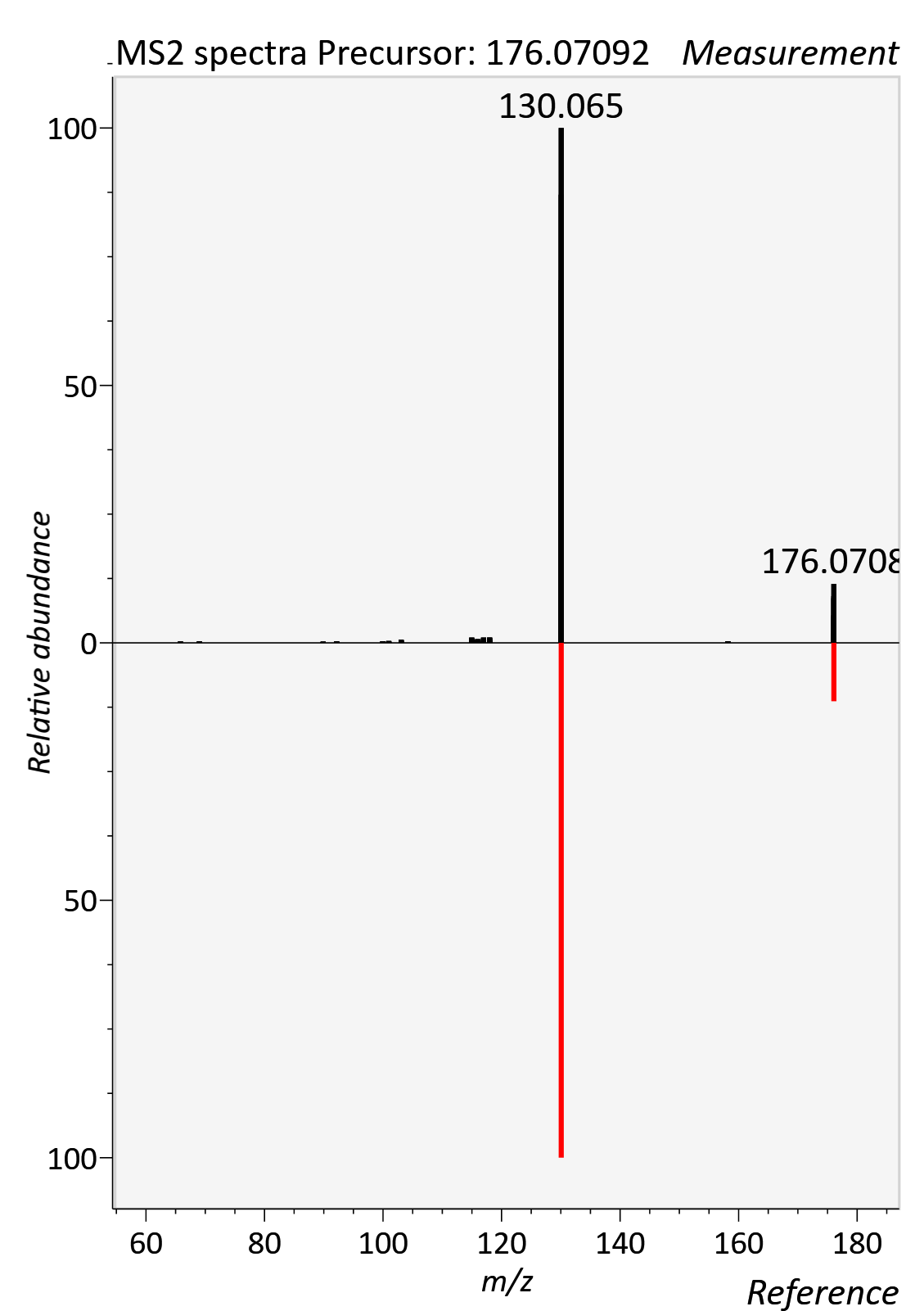


4-Trimethylammoniobutanoic acid

RT std: 3.36min, RT experimental: 3.60min, RT Δ 0.06min


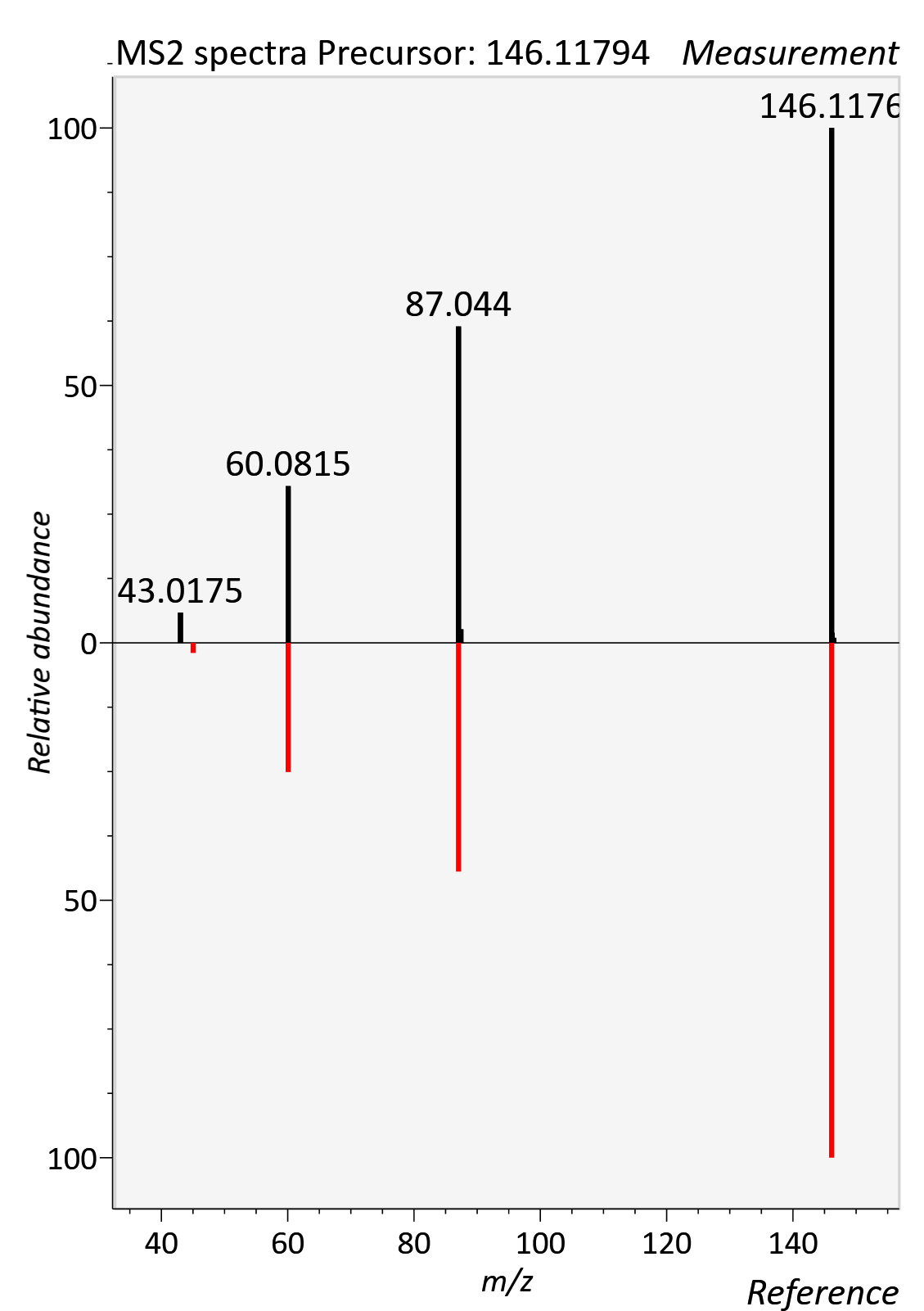


5-AVAB

RT std: 2.39min, RT experimental: 2.27min, RT Δ 0.12min


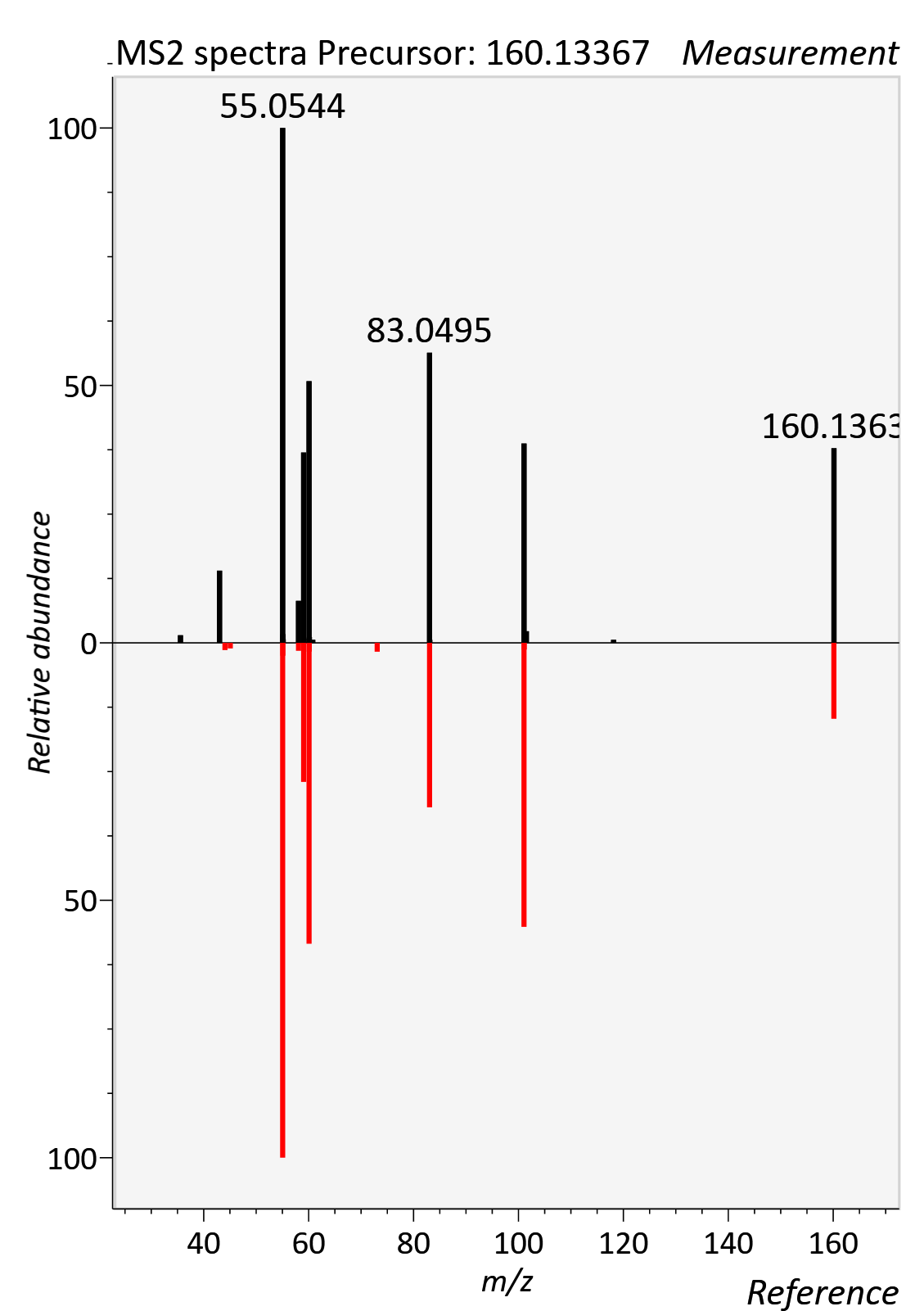


AC 05:0 (Isovalerylcarnitine)

RT std: 1.28min, RT experimental: 1.40min, RT Δ 0.12min


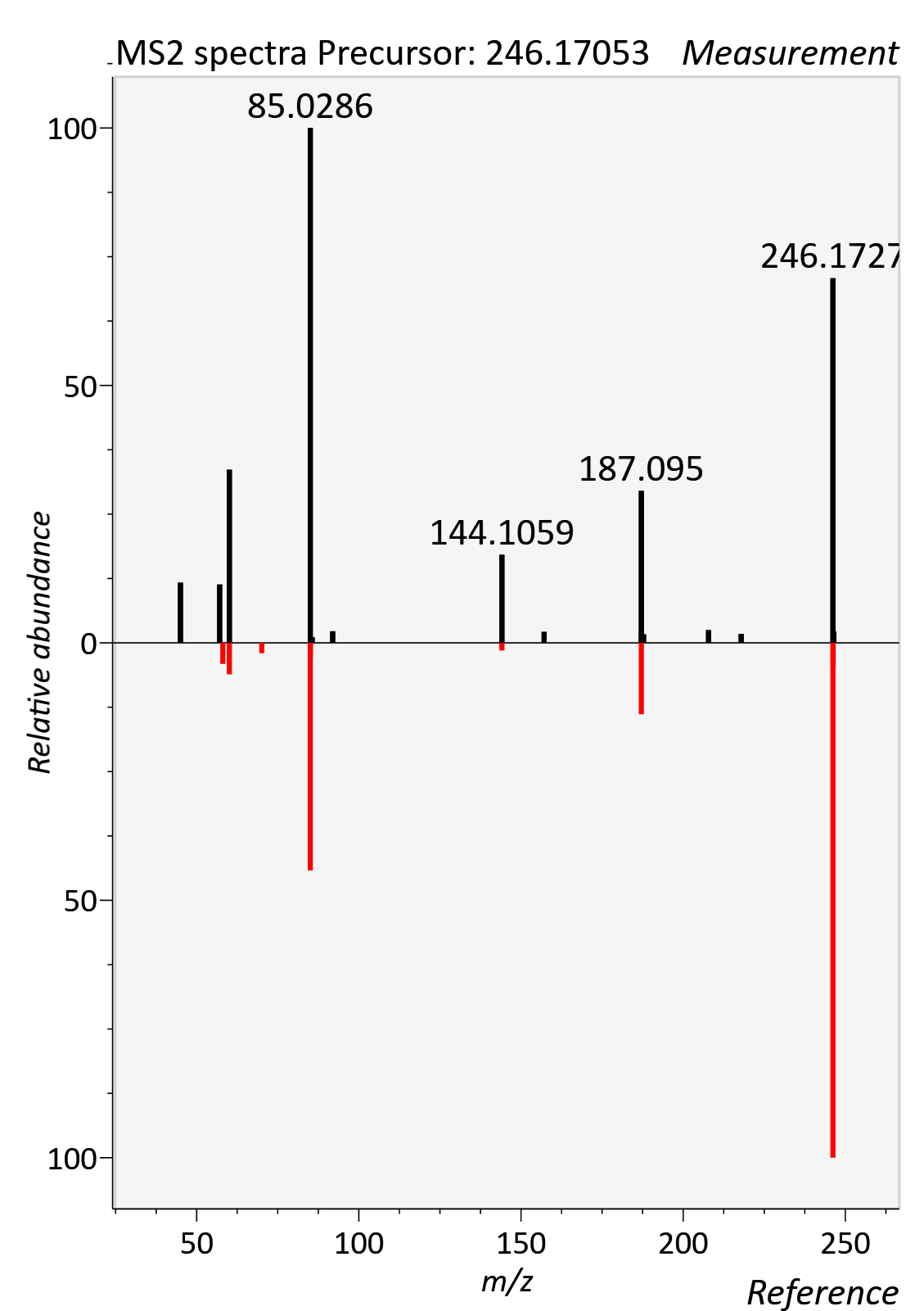


7a-hydroxy-3-oxo-4-cholestenoic acid

RT std: 9.71min, RT experimental: 9.94min, RT Δ 0.23min


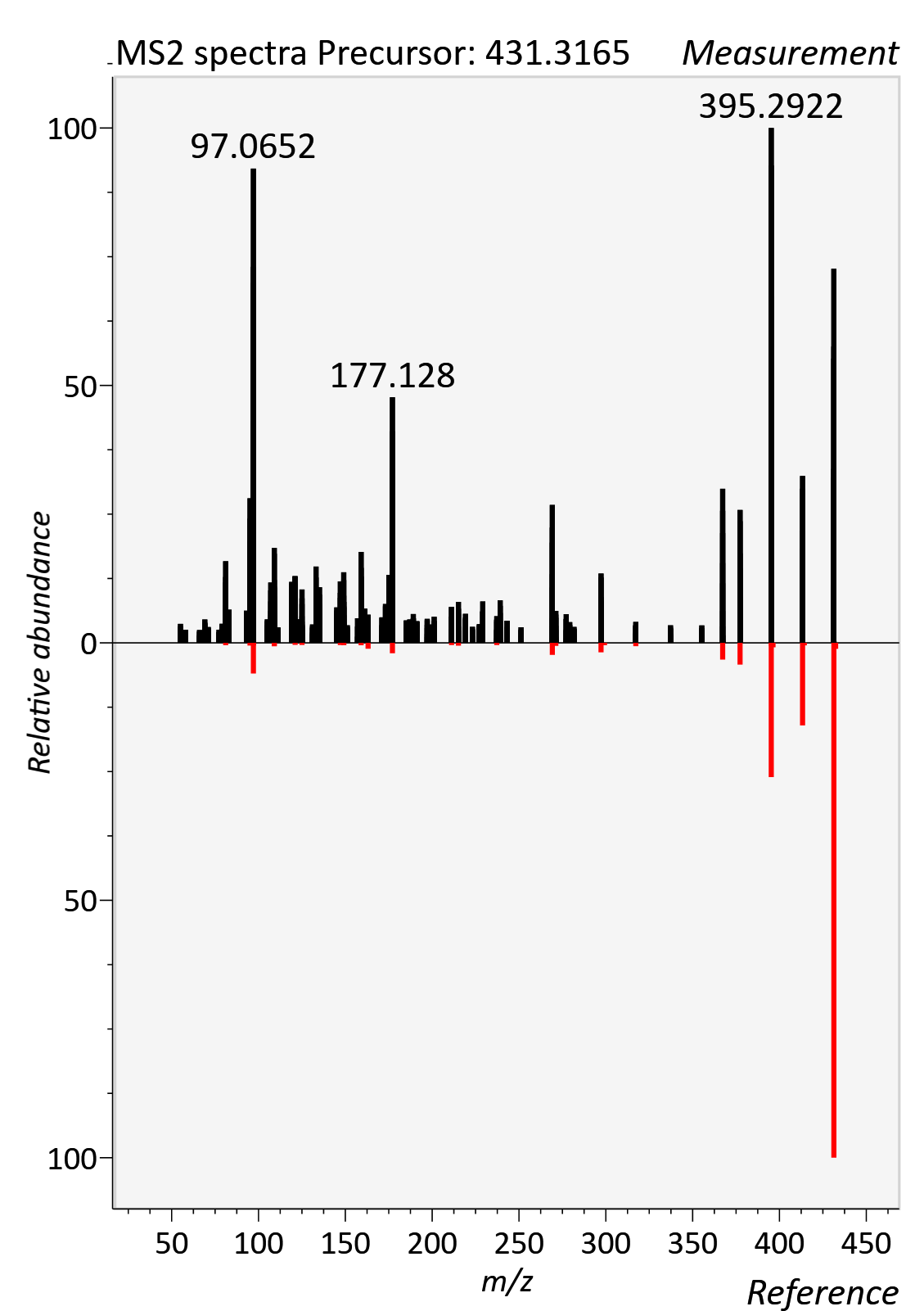


AC 04:0 (Butanoylcarnitine)

RT std: 1.85min, RT experimental: 1.77min, RT Δ 0.08min


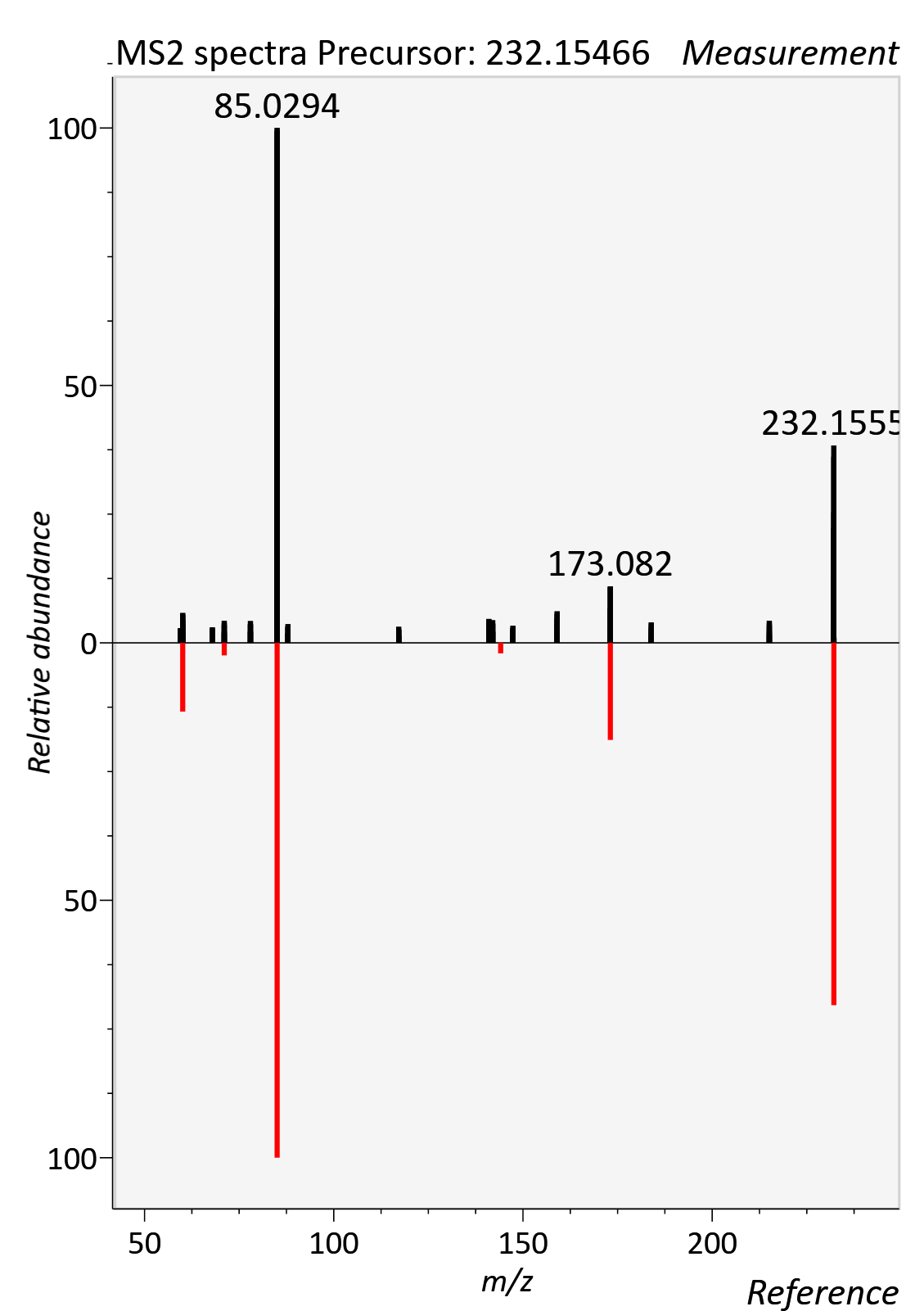


AC 06:0 (Hexanoylcarnitine)

RT std: 4.09min, RT experimental: 4.20min, RT Δ 0.11min


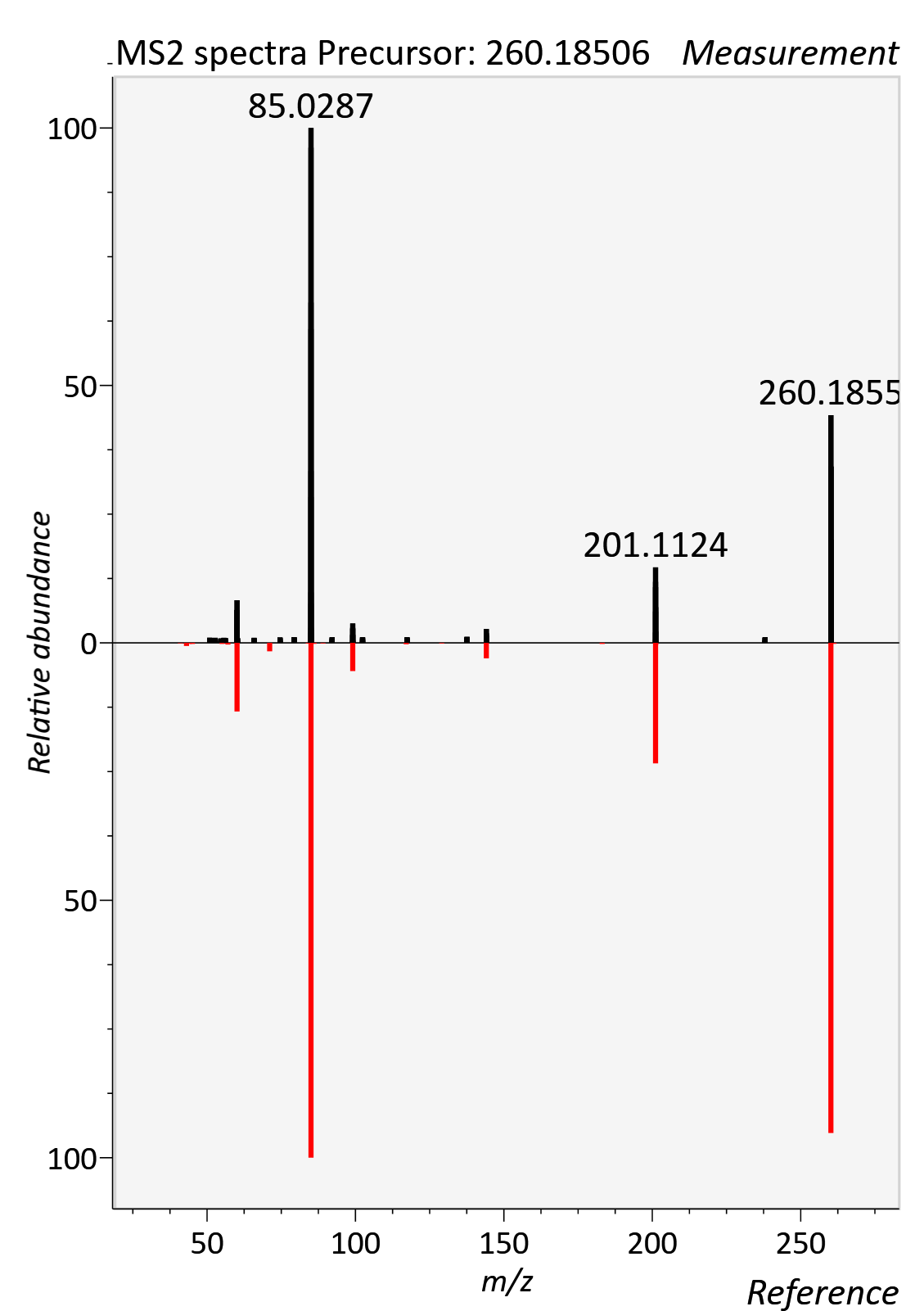


AC 08:0 (Octanoylcarnitine)

RT std: 5.89, RT experimental: 6.02min, RT Δ 0.13min


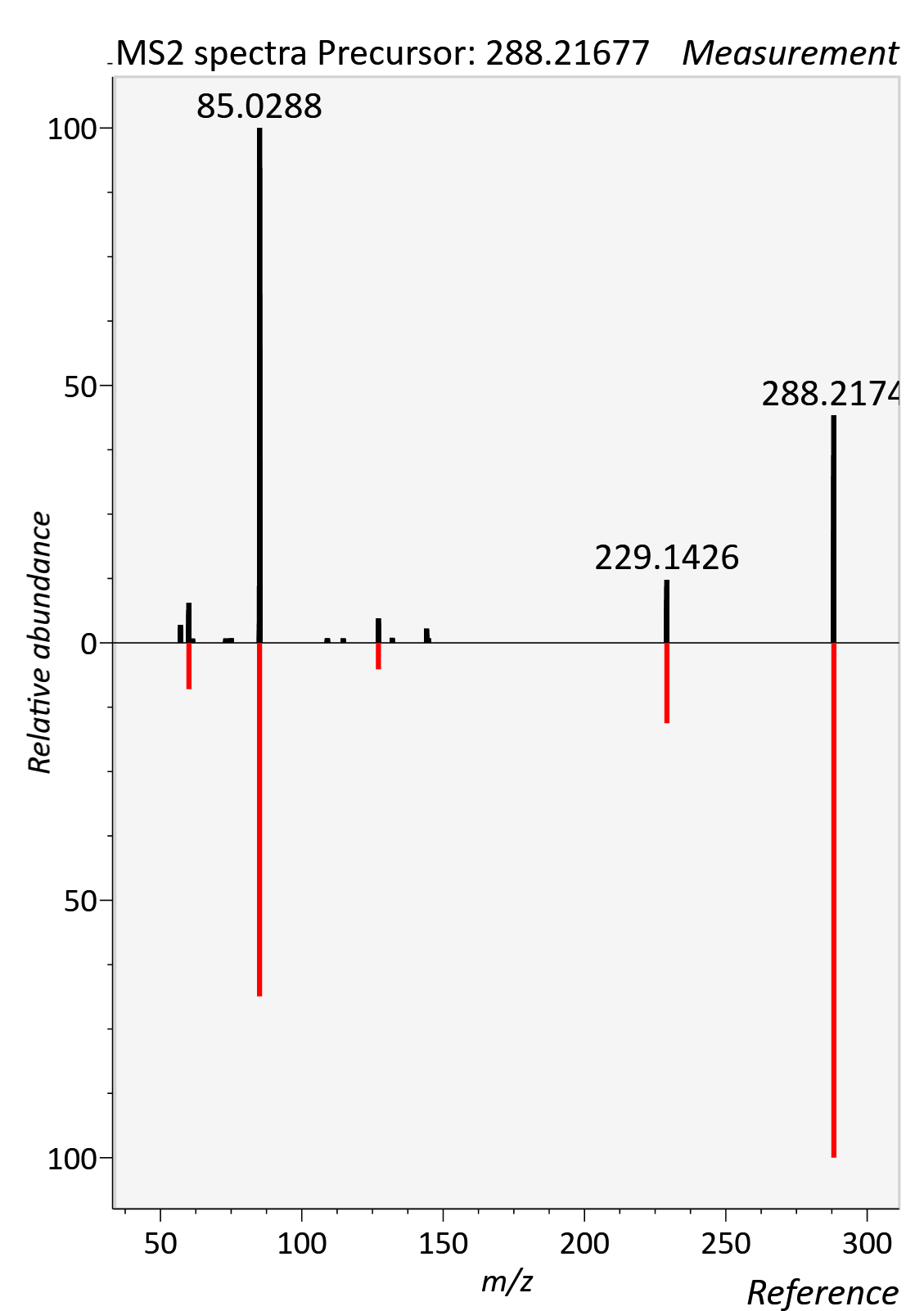


AC 08:1 (Octenoyl-L-carnitine)

RT std: 5.56min, RT experimental: 5.20min, RT Δ 0.23min


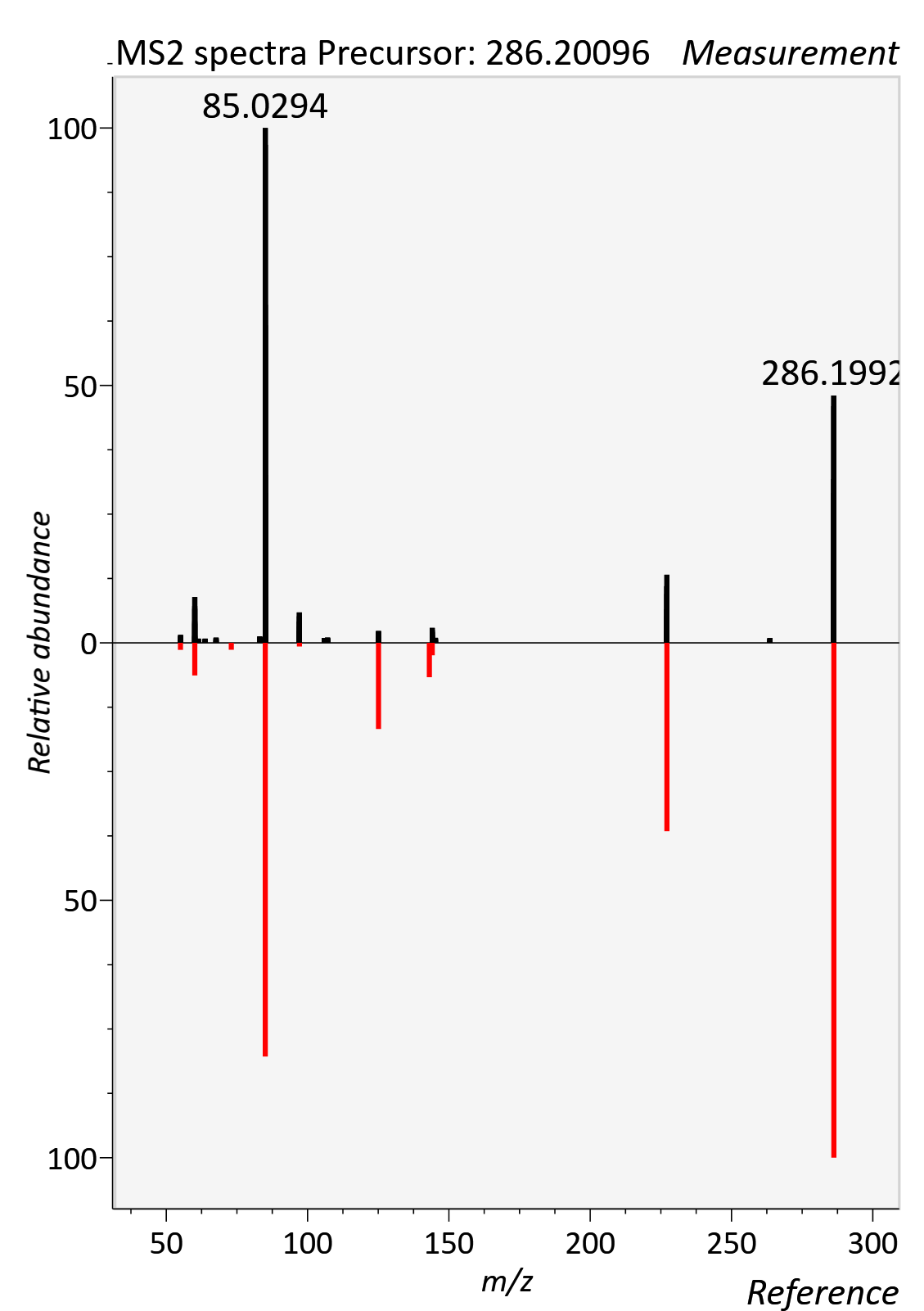


AC 10:0 (Decanoylcarnitine)

RT std: 7.17min, RT experimental: 7.28min, RT Δ 0.11min


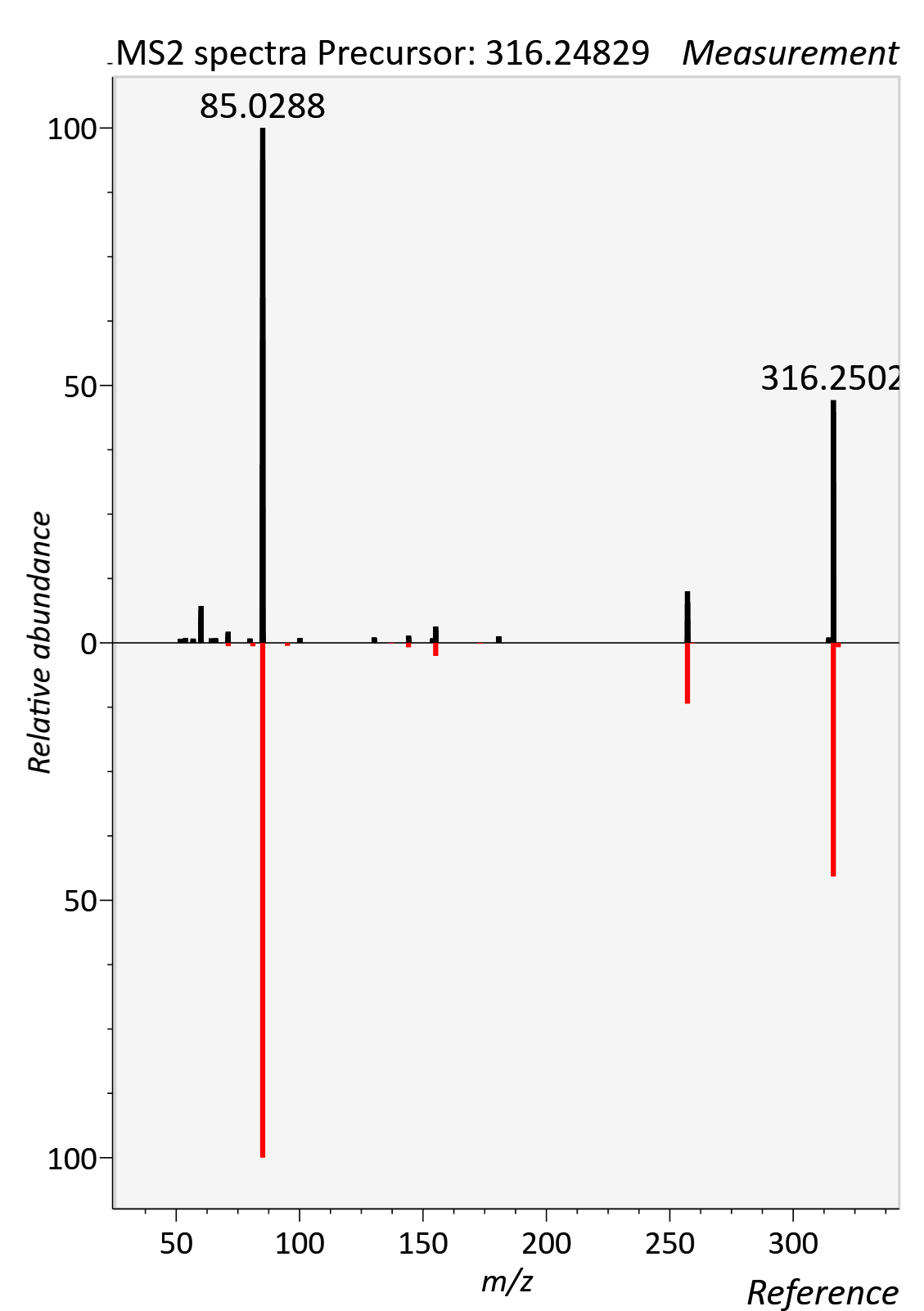


AC 12:0 (Dodecanoylcarnitine)

RT std: 8.09min, RT experimental: 8.17min, RT Δ 0.08min


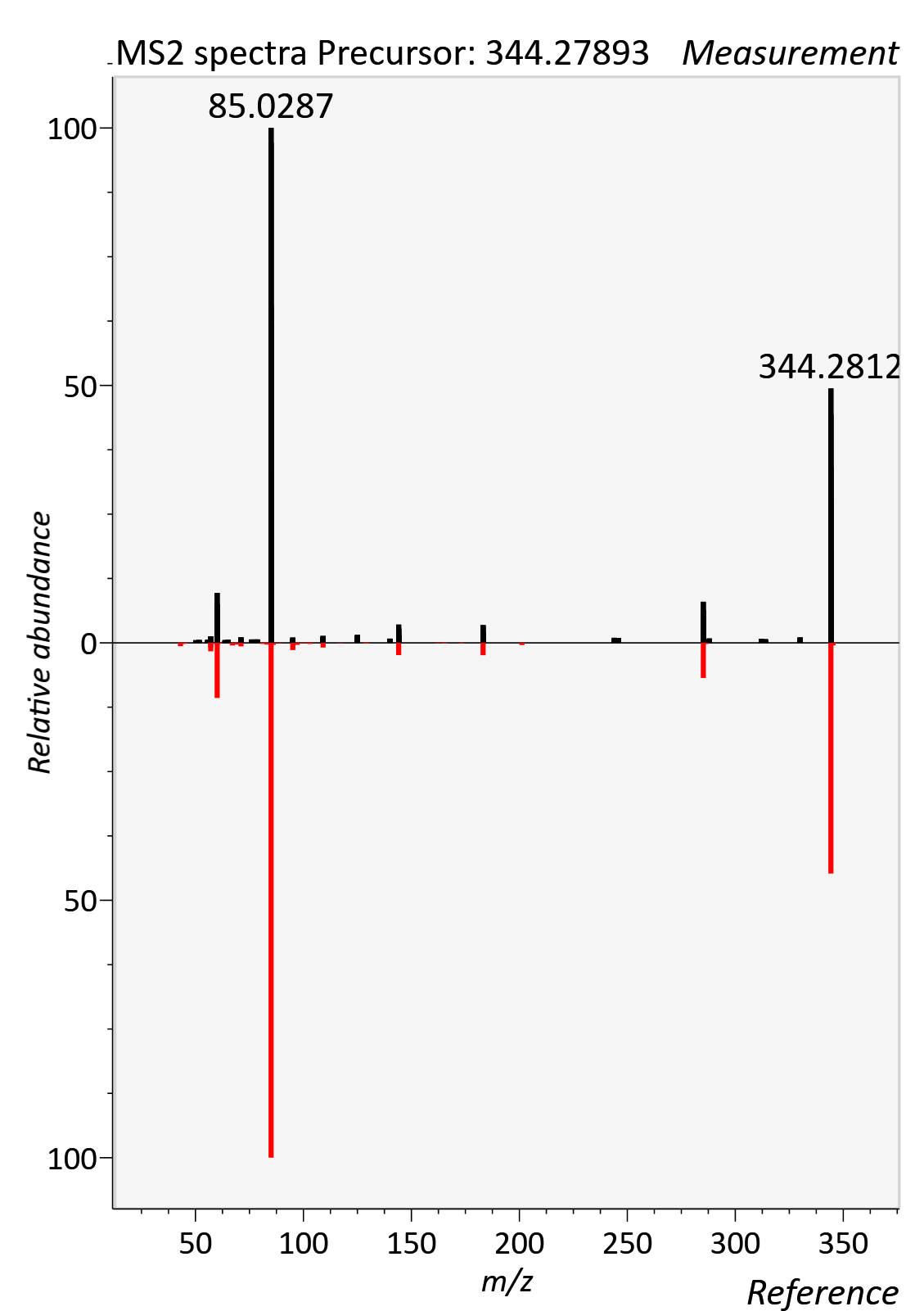


AC 12:1 (Dodecenoylcarnitine)

RT std: 7.91min, RT experimental: 7.76min, RT Δ 0.15min


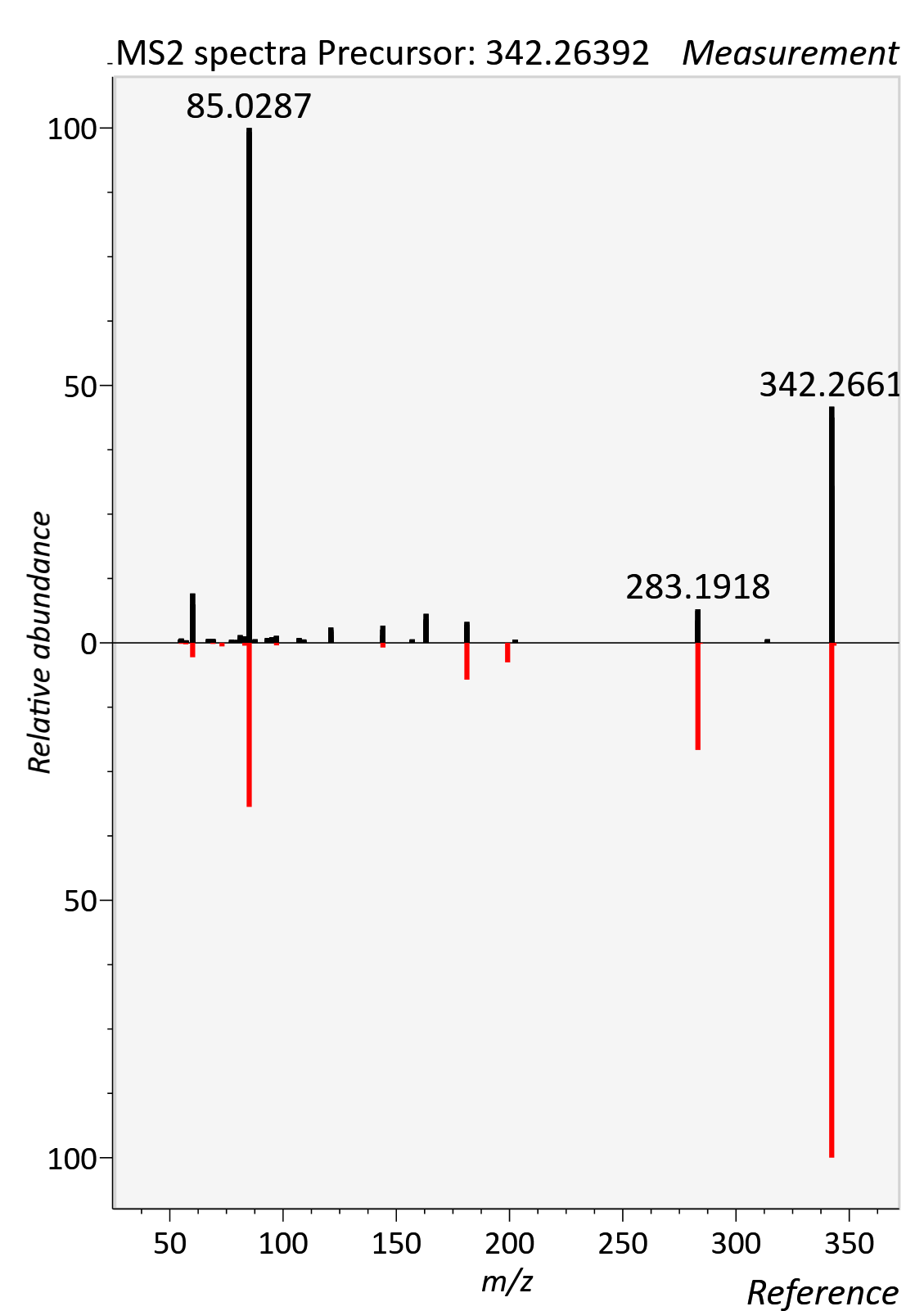


AC 14:0 (Tetradecanoyl-L-carnitine)

RT std: 8.60min, RT experimental: 8.80min, RT Δ 0.20min


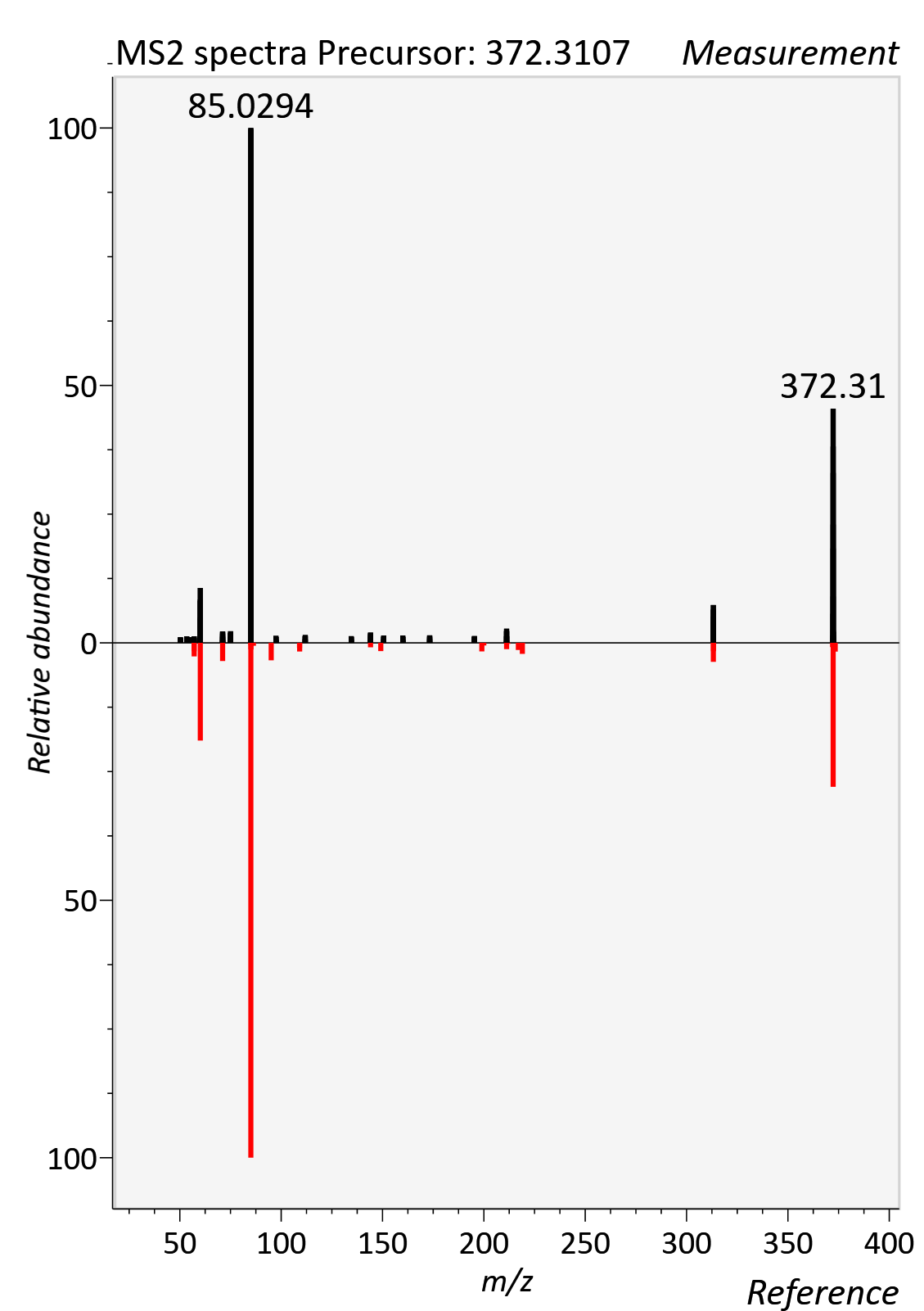


AC 14:1 (Tetradecenoylcarnitine)

RT std: 8.65min, RT experimental: 8.48min, RT Δ 0.23min


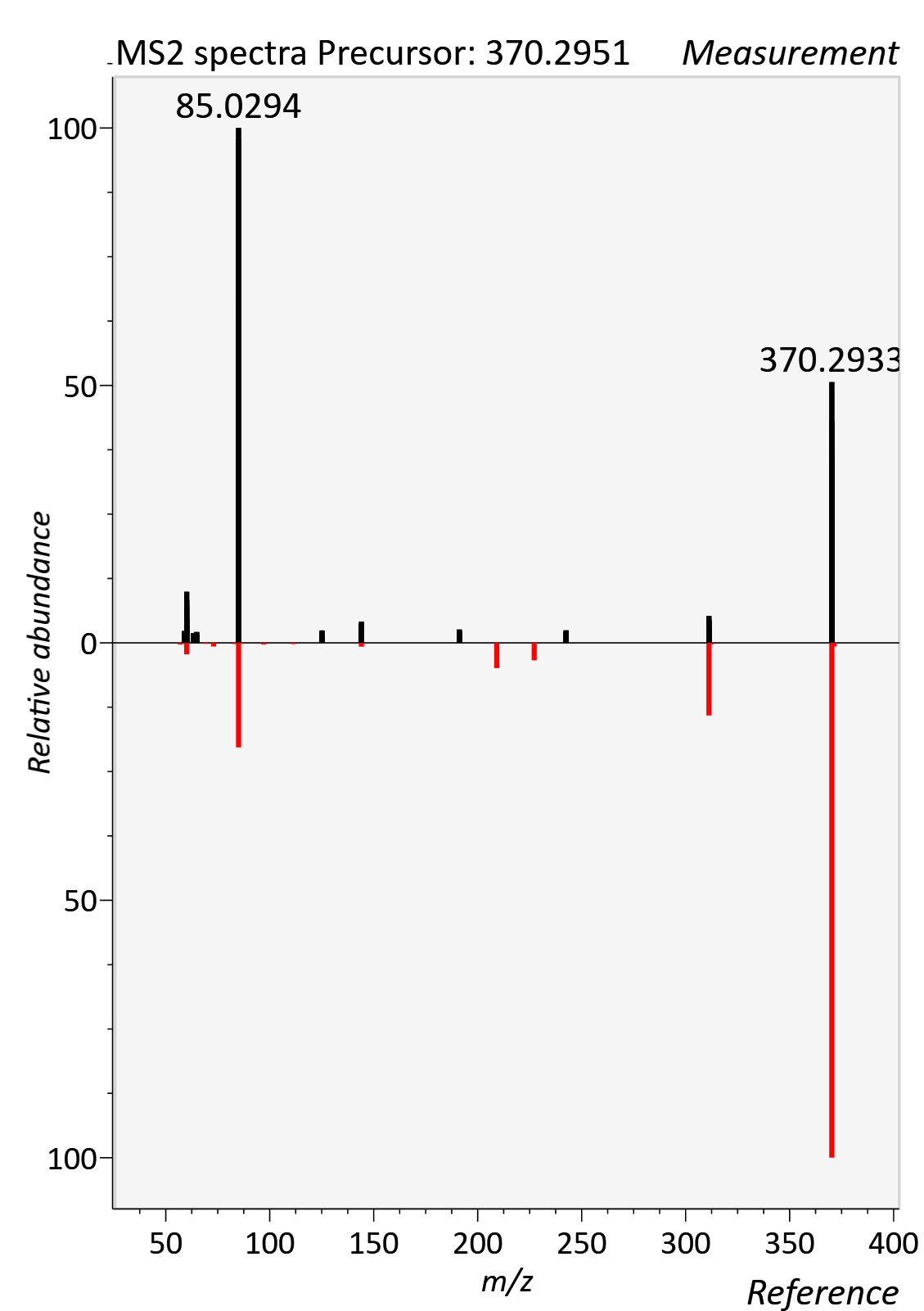


AC 16:0 (Palmitoylcarnitine)

RT std: 9.13min, RT experimental: 9.30min, RT Δ 0.20min


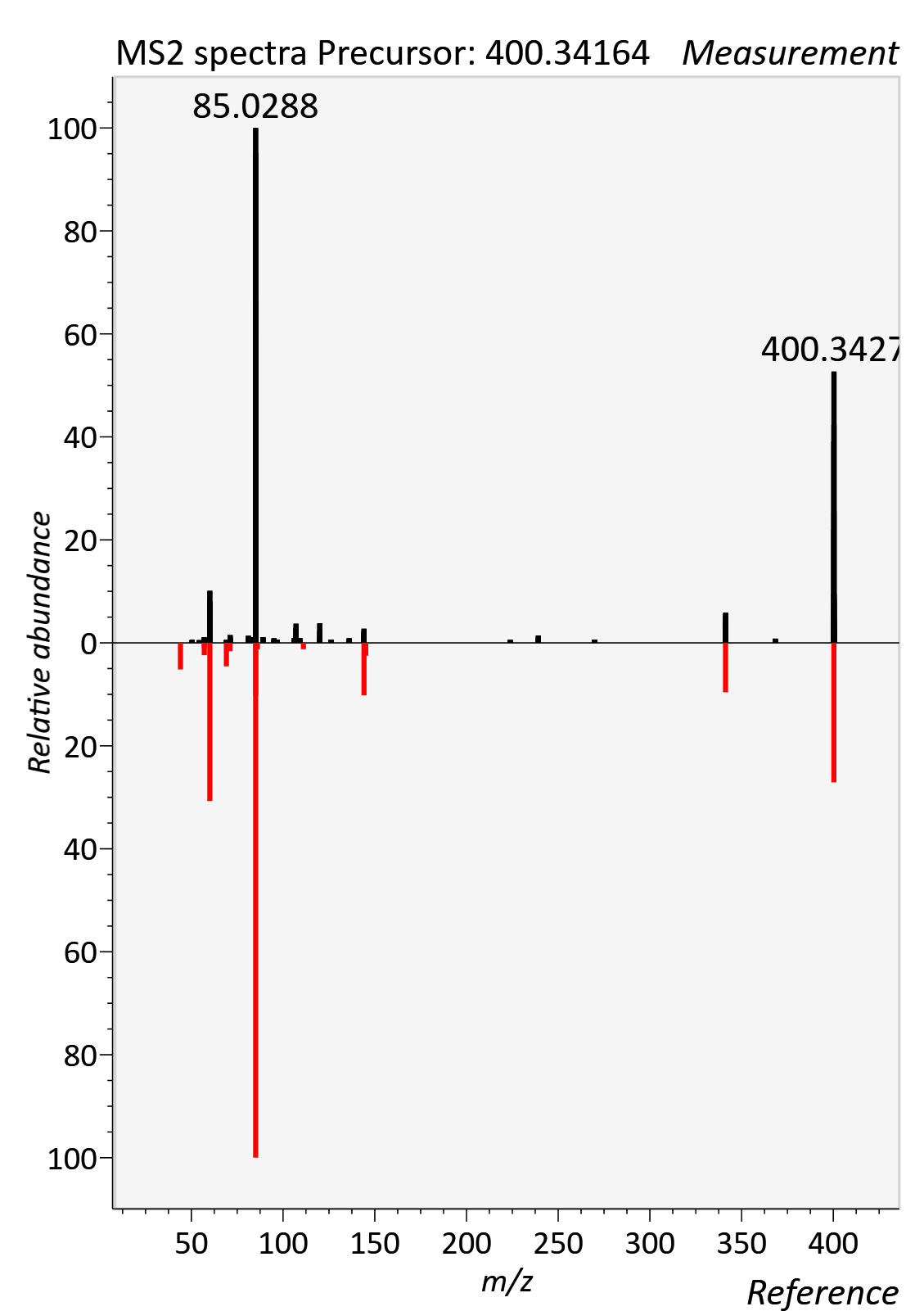


AC 18:1 (Oleylcarnitine)

RT std: 9.34min, RT experimental: 9.44min, RT Δ 0.10min


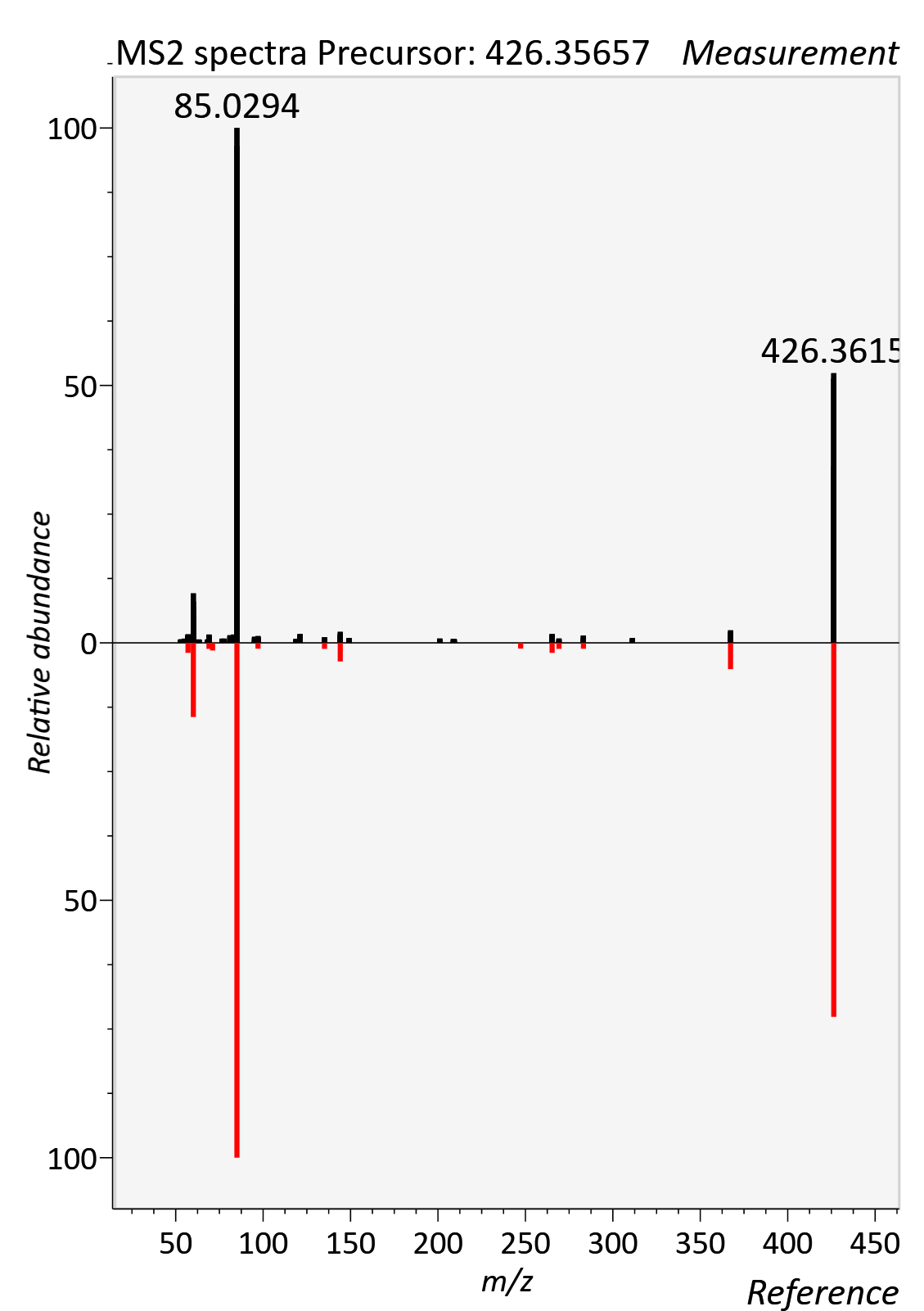


AC 18:2 (Linoleylcarnitine)

RT std: 9.15min, RT experimental: 9.17min, RT Δ 0.02min


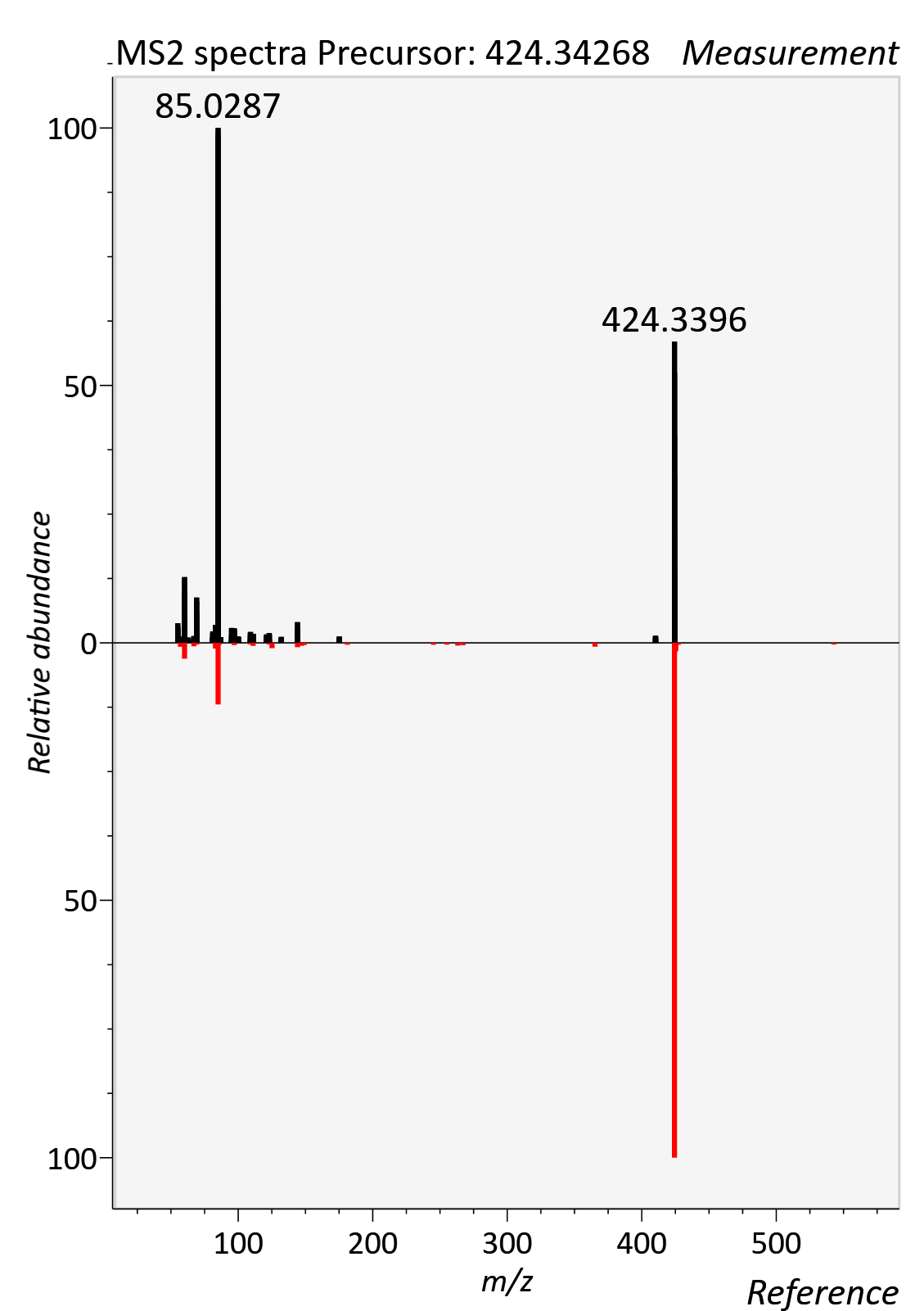


Acetylcarnitine

RT std: 2.71min, RT experimental: 3.25min, RT Δ 0.54min


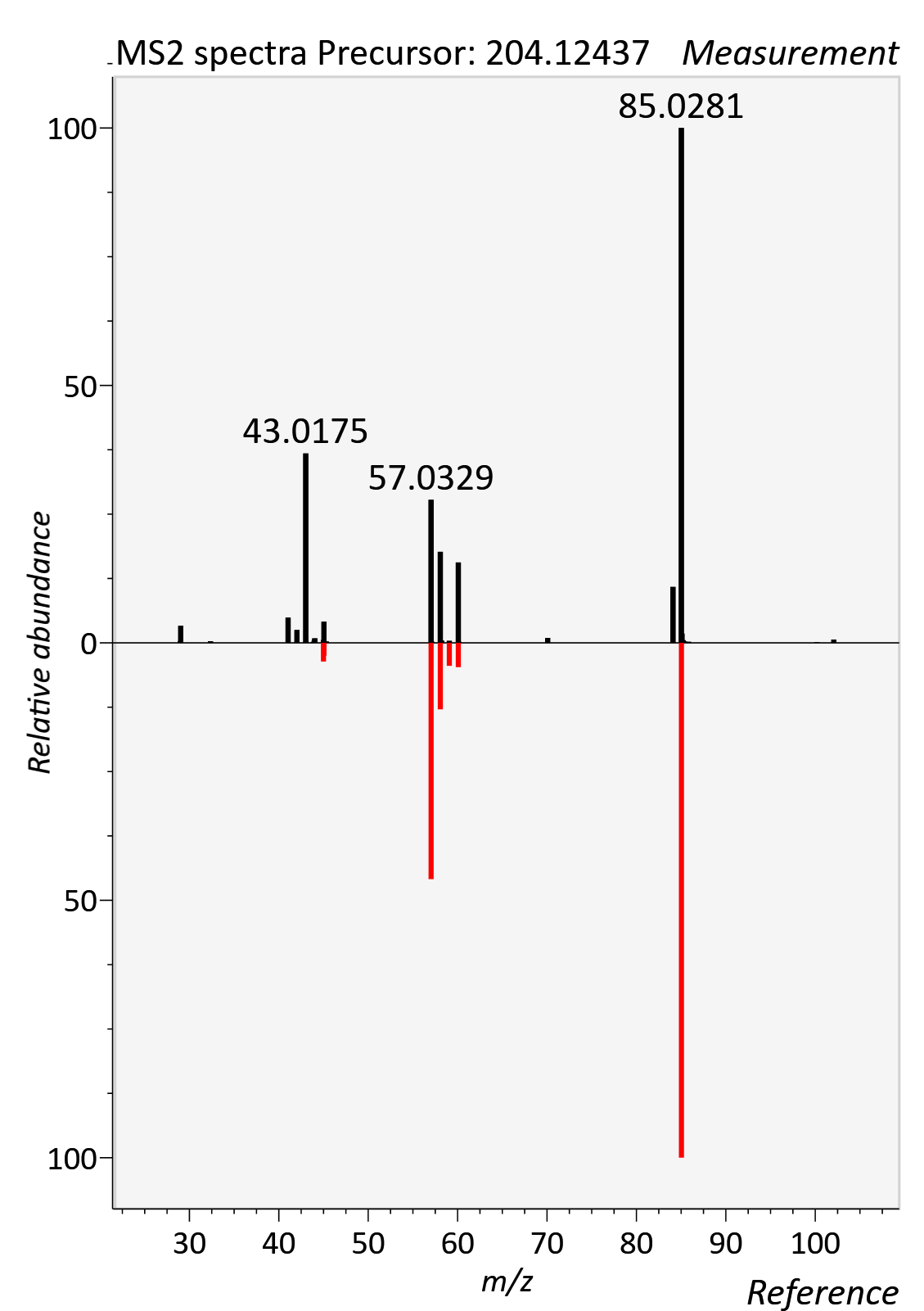


Caffeine

RT std: 3.67min, RT experimental: 3.80min, RT Δ 0.13min


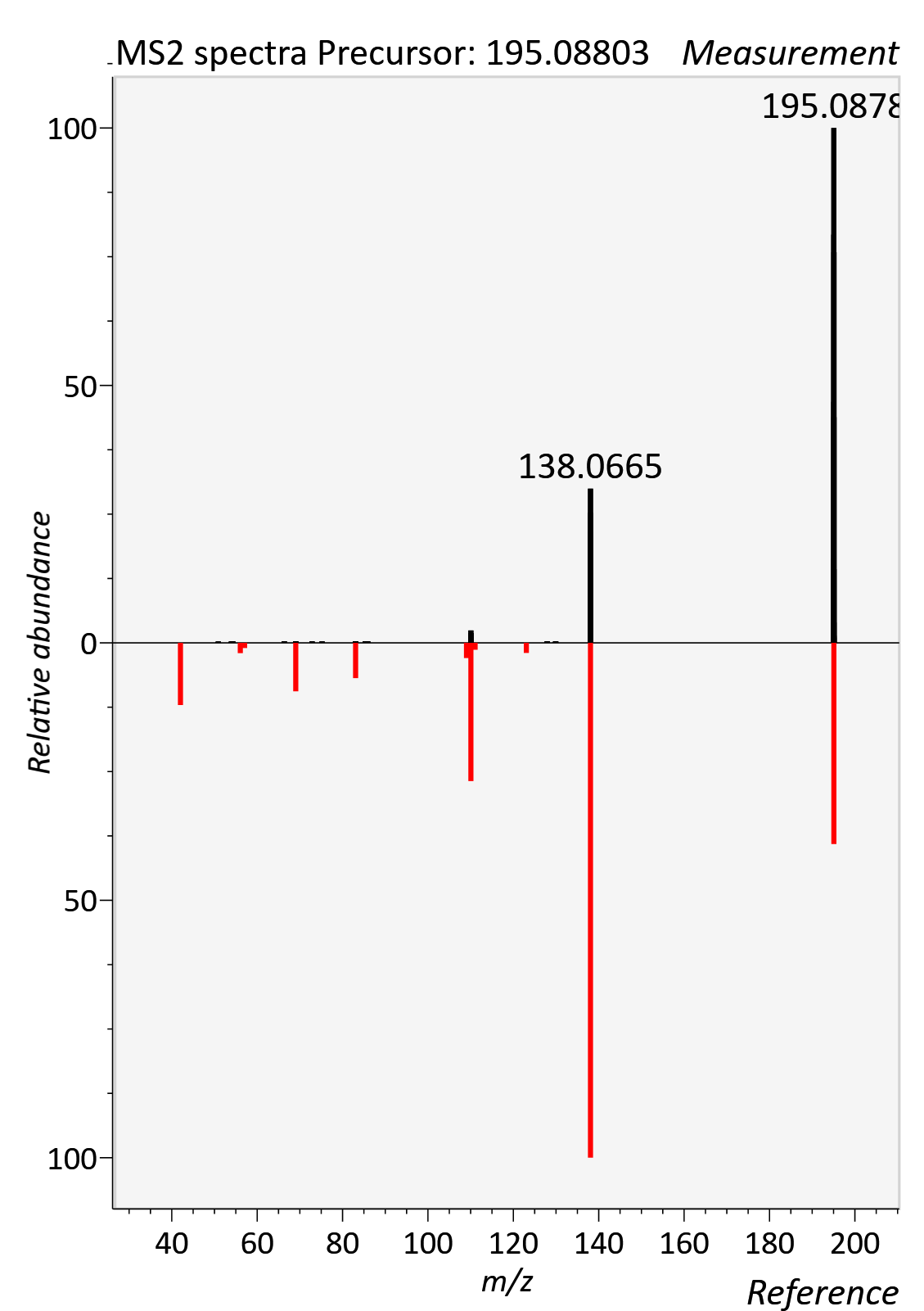


Citrulline

RT std: 6.35min, RT experimental: 6.50min, RT Δ 0.15min


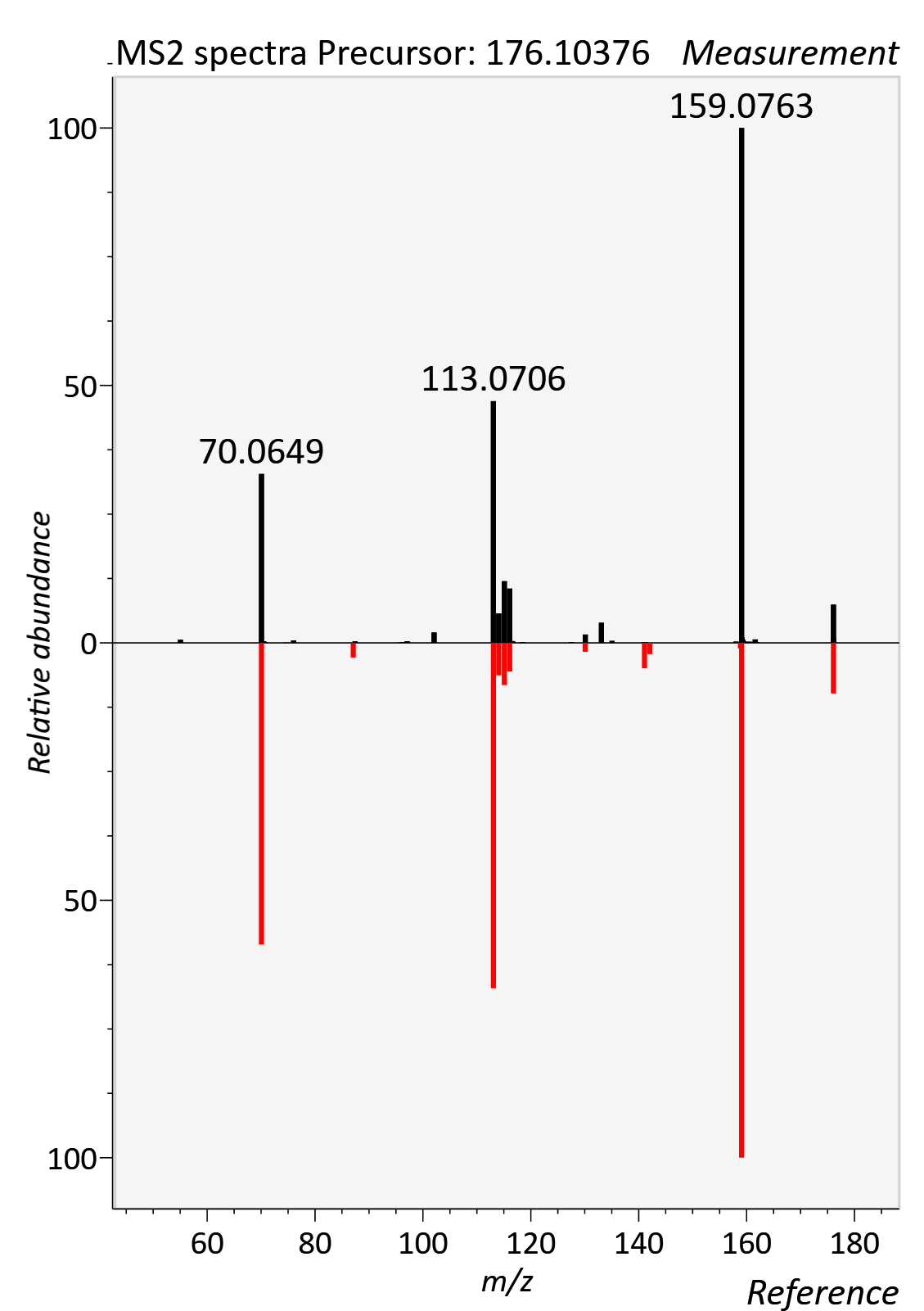


Cholic acid

RT std: 9.08min, RT experimental: 9.28min, RT Δ 0.20min


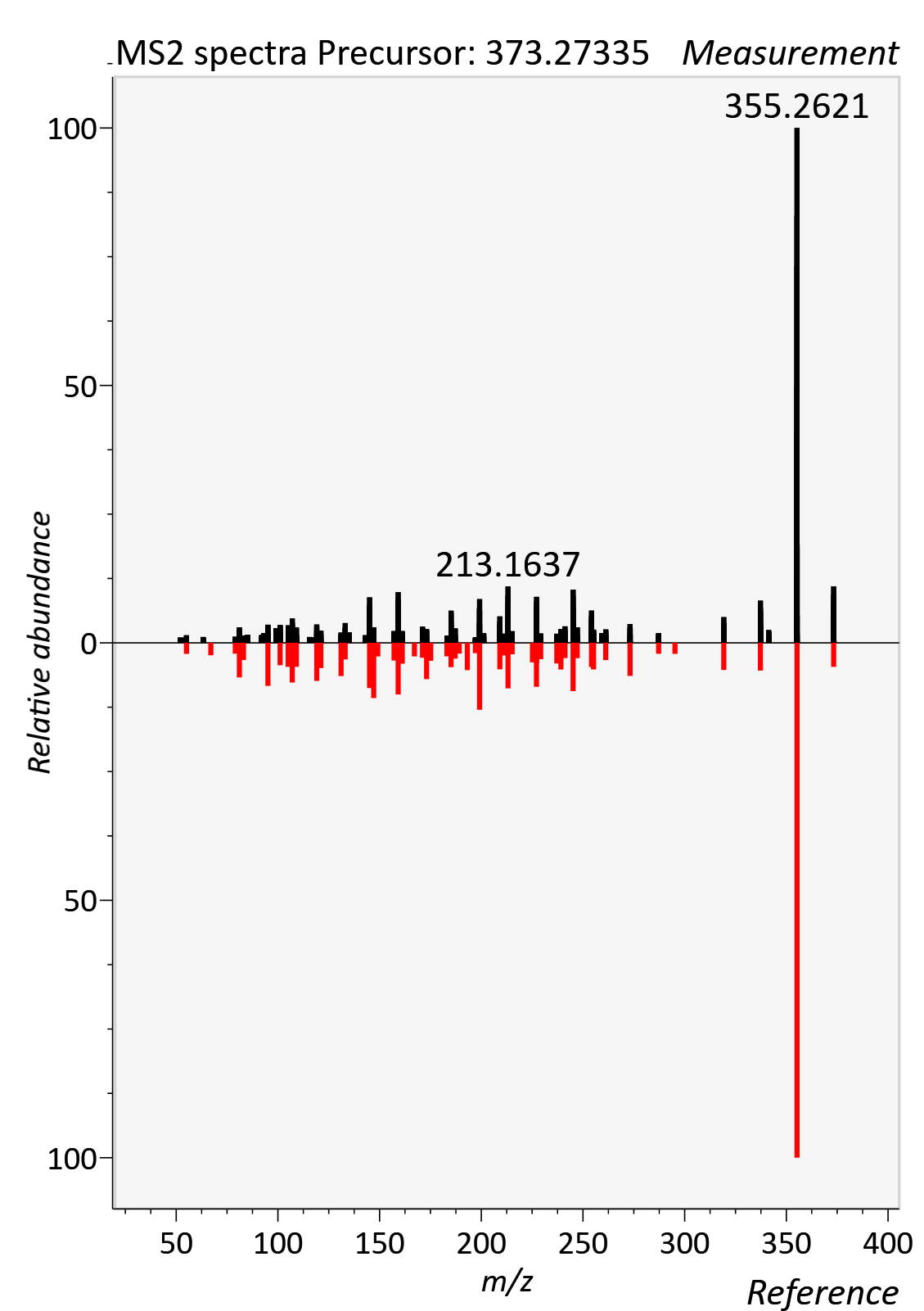


Cortisol

RT std: 6.79min, RT experimental: 6.99min, RT Δ 0.20min


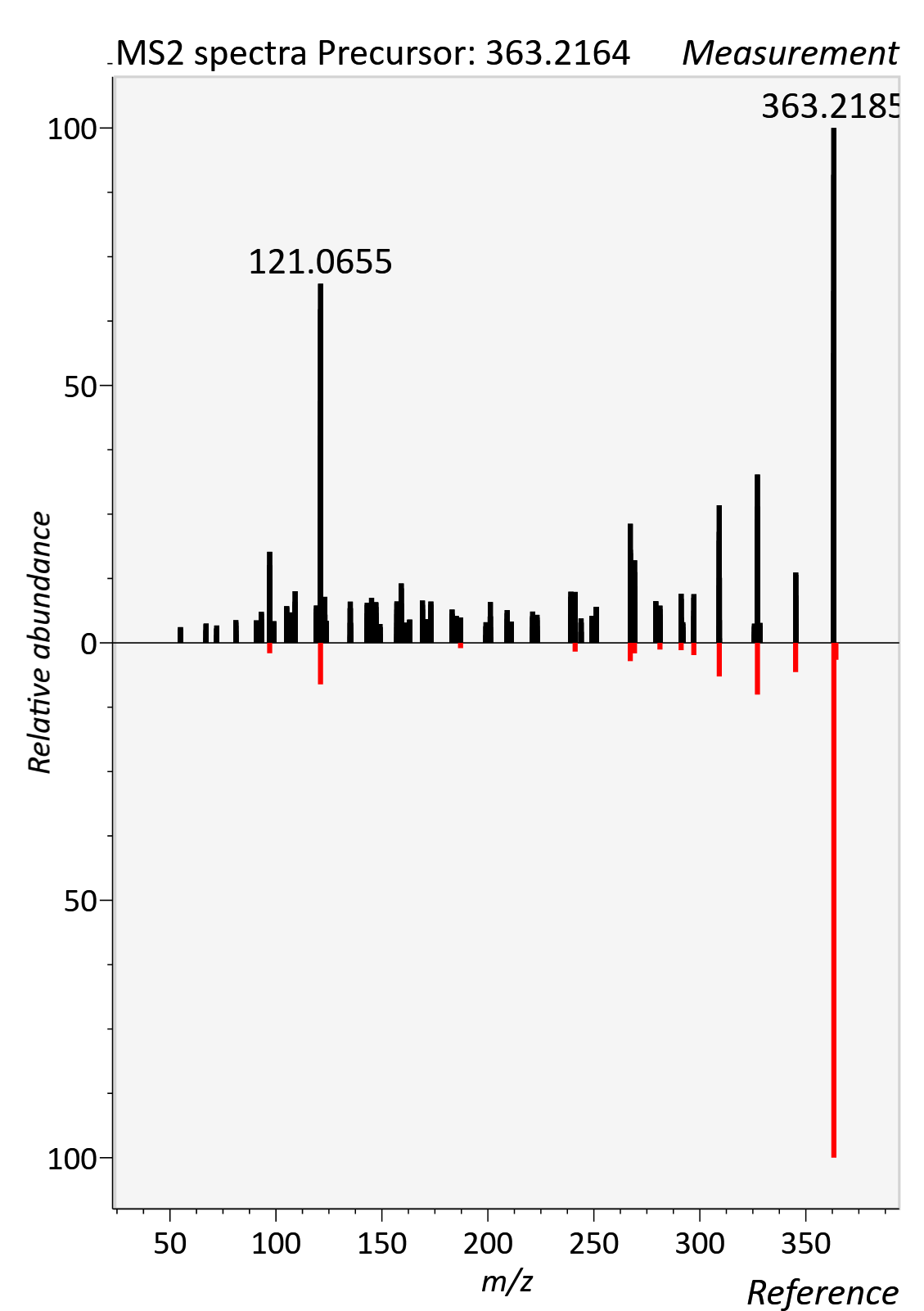


Cortisone, [M+FA-H]-

RT std: 6.55min, RT experimental: 6.73min, RT Δ 0.22min


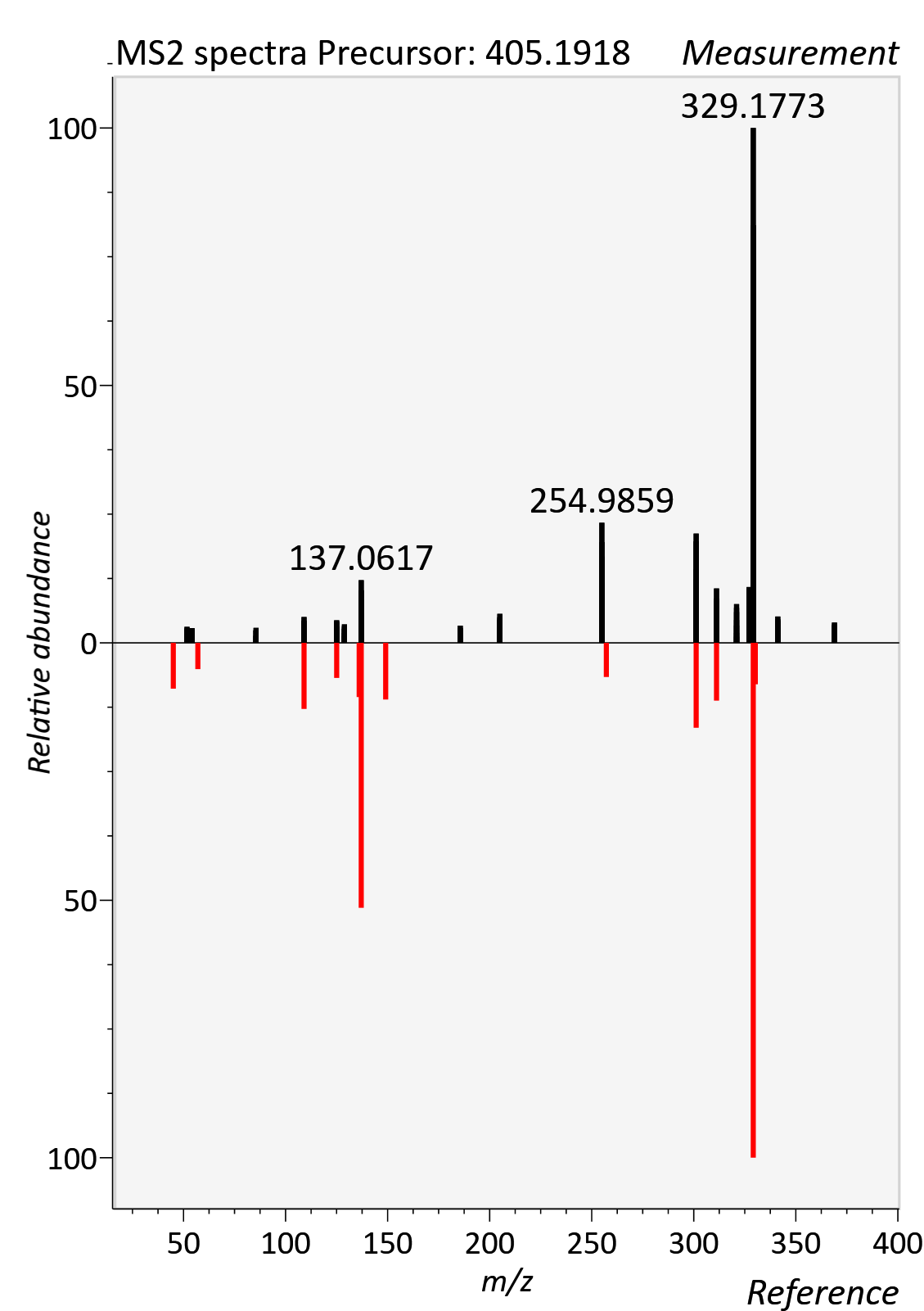


Creatinine

RT std: 1.22min, RT experimental: 1.35min, RT Δ 0.13min


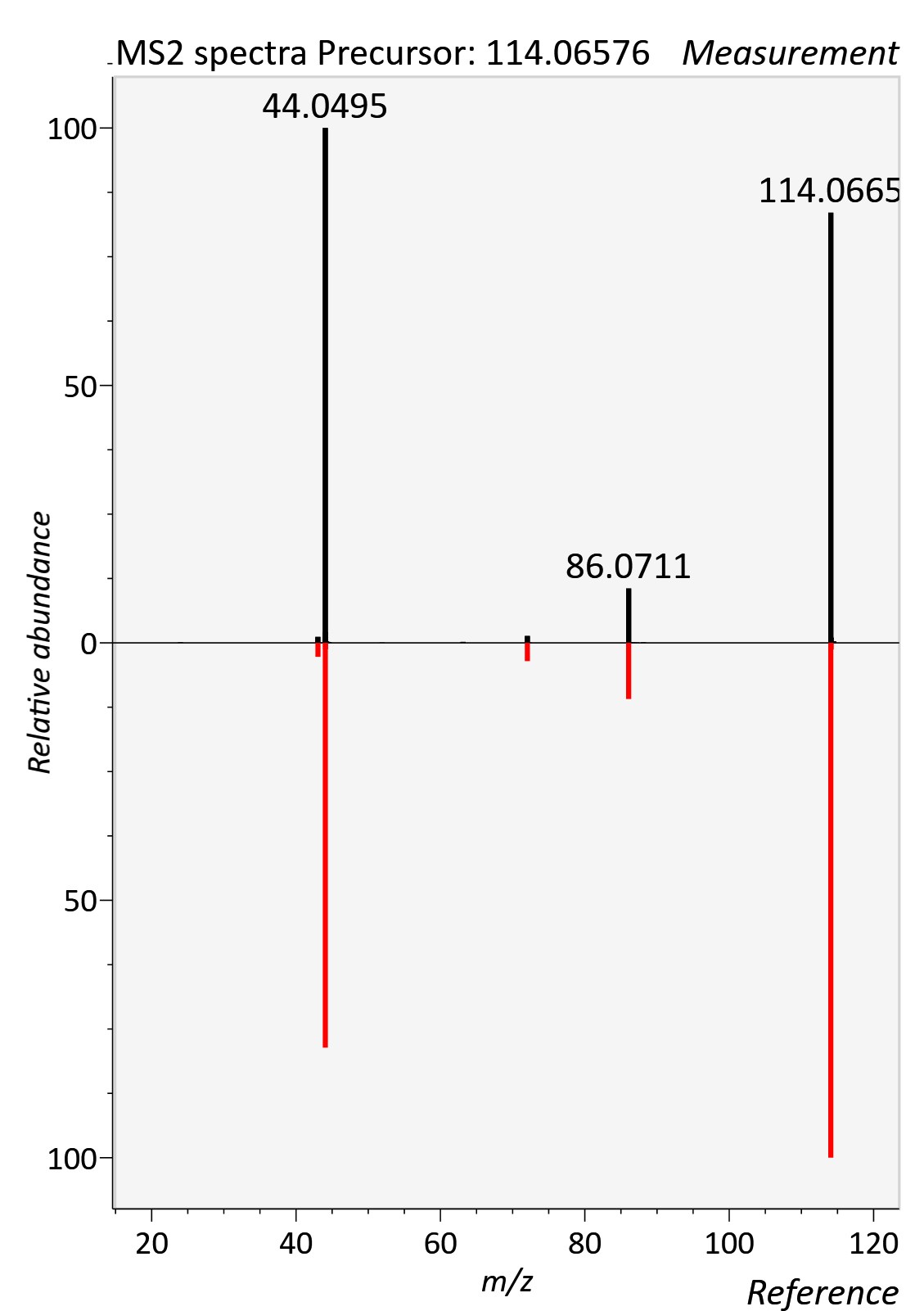


Deoxycholic acid

RT std: 9.71min, RT experimental: 9.90min, RT Δ 0.19min


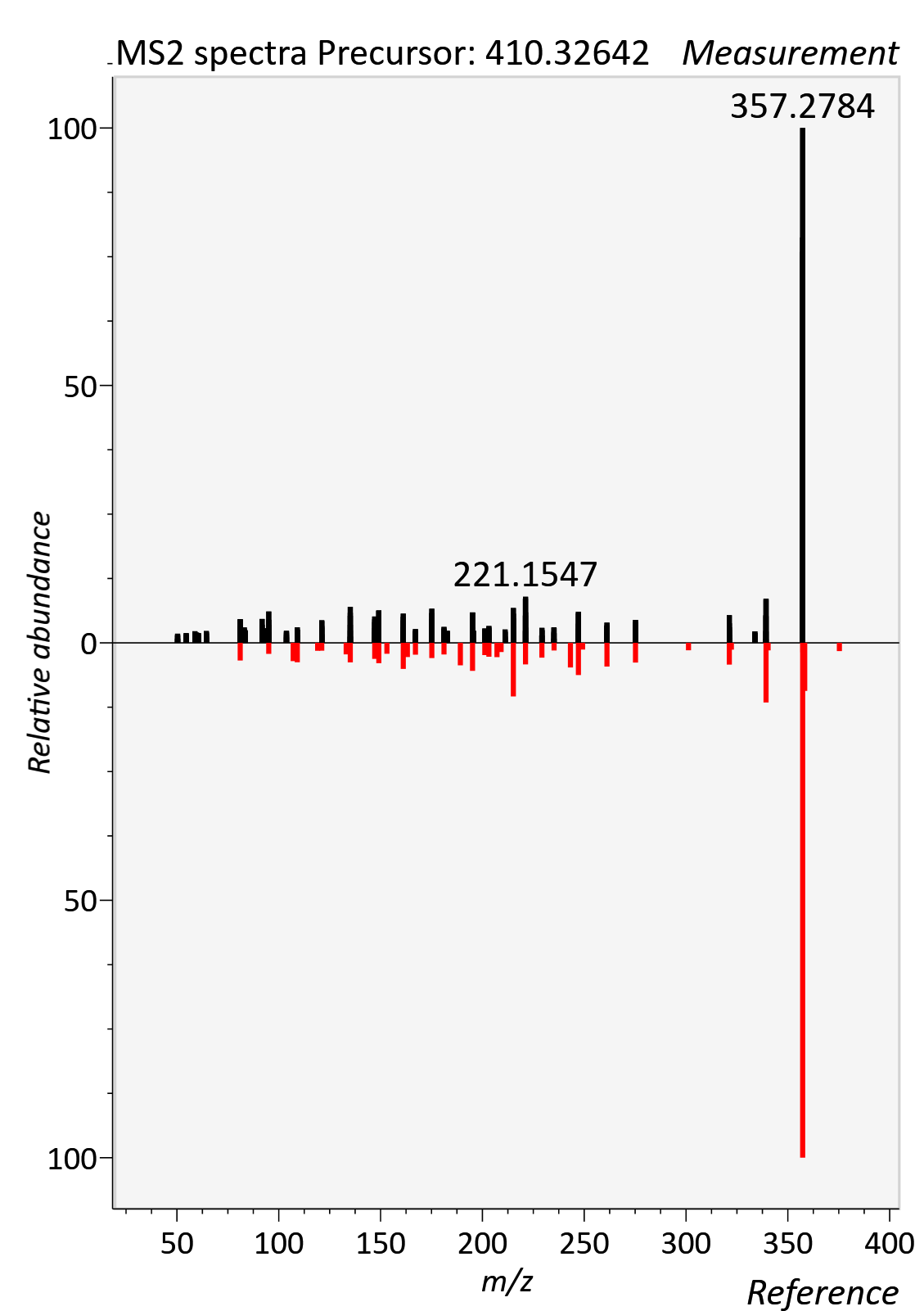


FA 18:1 (Octadecenoic acid)

RT std: 11.23min, RT experimental: 10.92min, RT Δ 0.31min


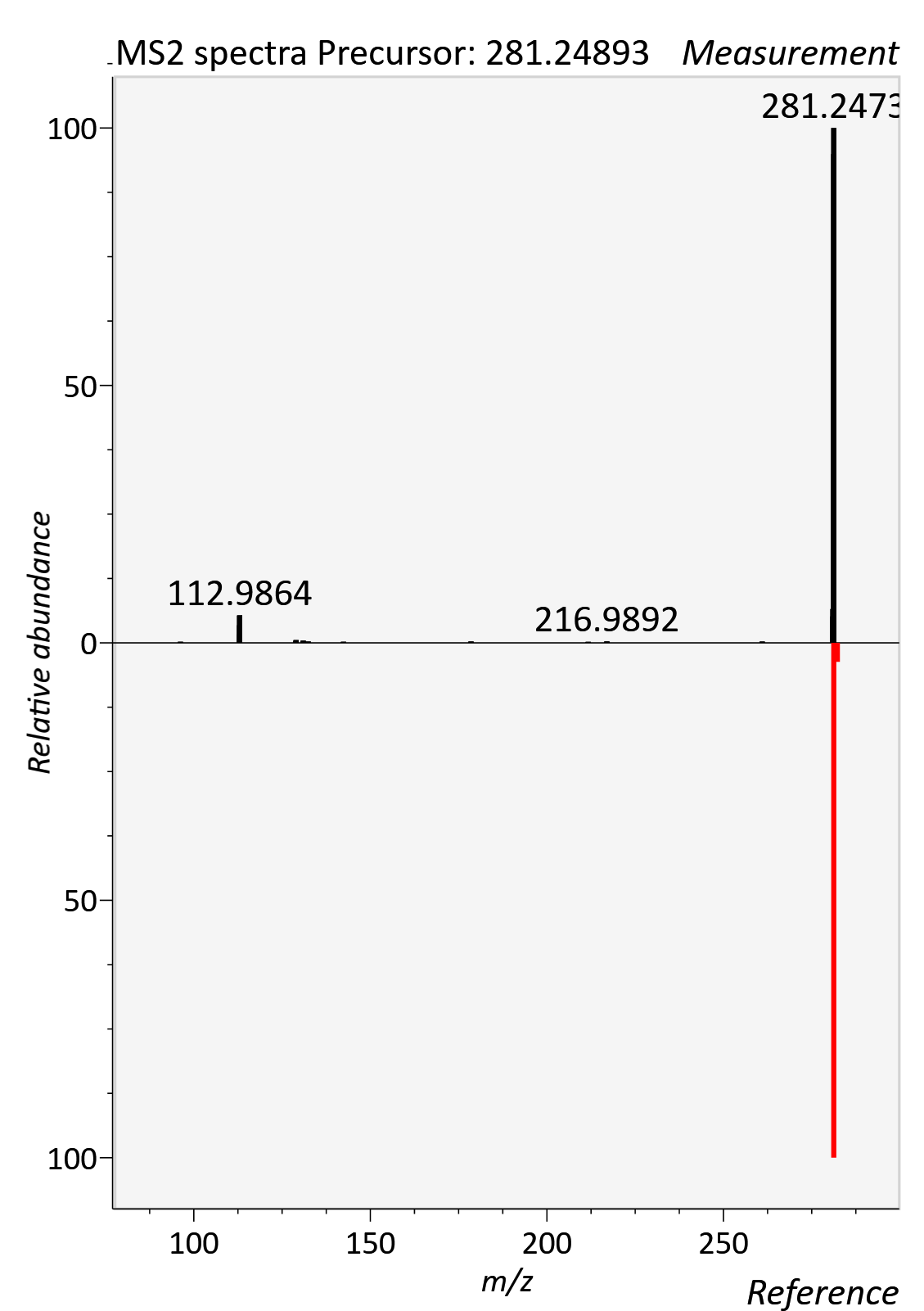


FA 22:6 (DHA)

RT std: 10.66min, RT experimental: 10.89min, RT Δ 0.23min


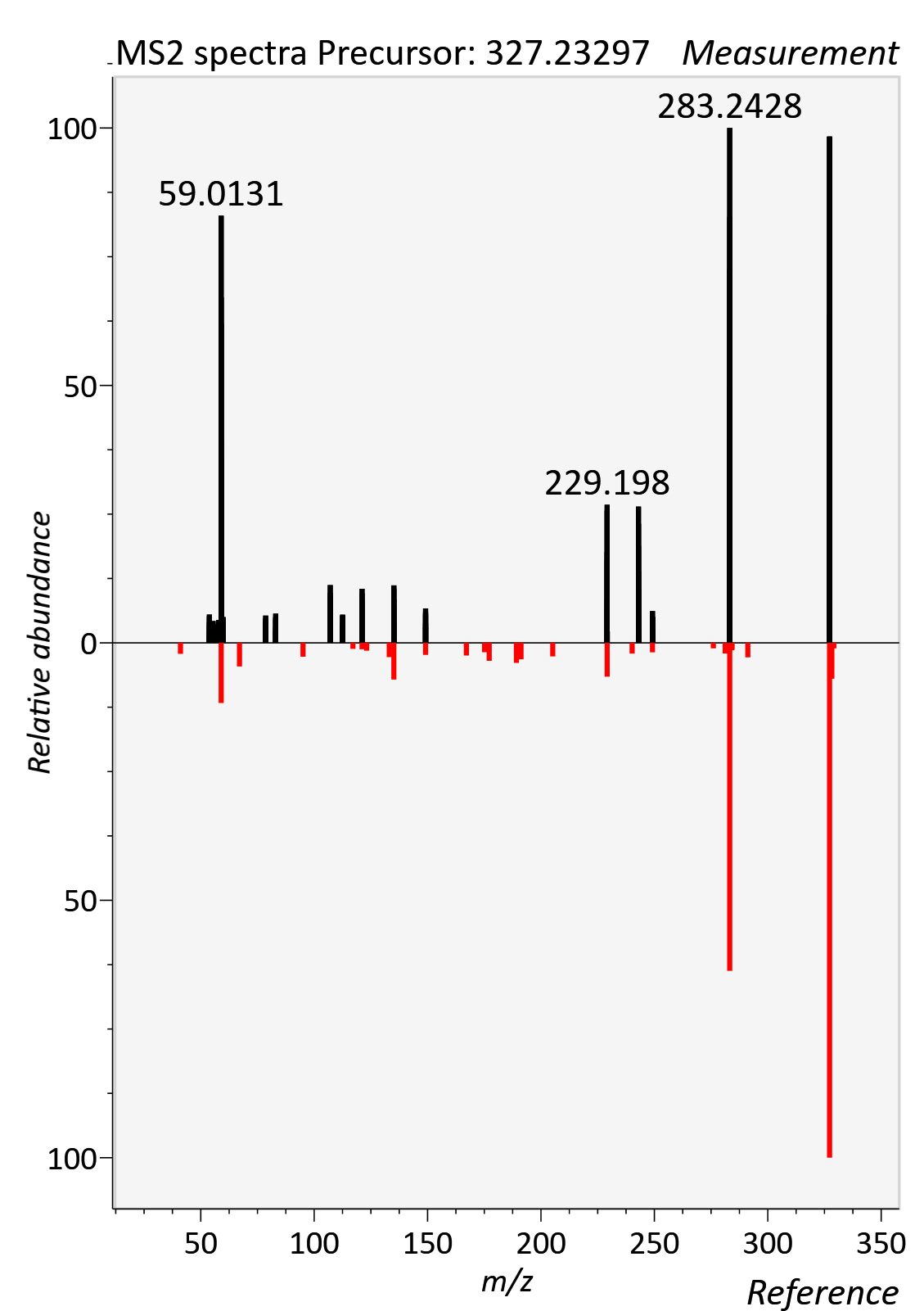


Glutamine

RT std: 6.13min, RT experimental: 6.27min, RT Δ 0.14min


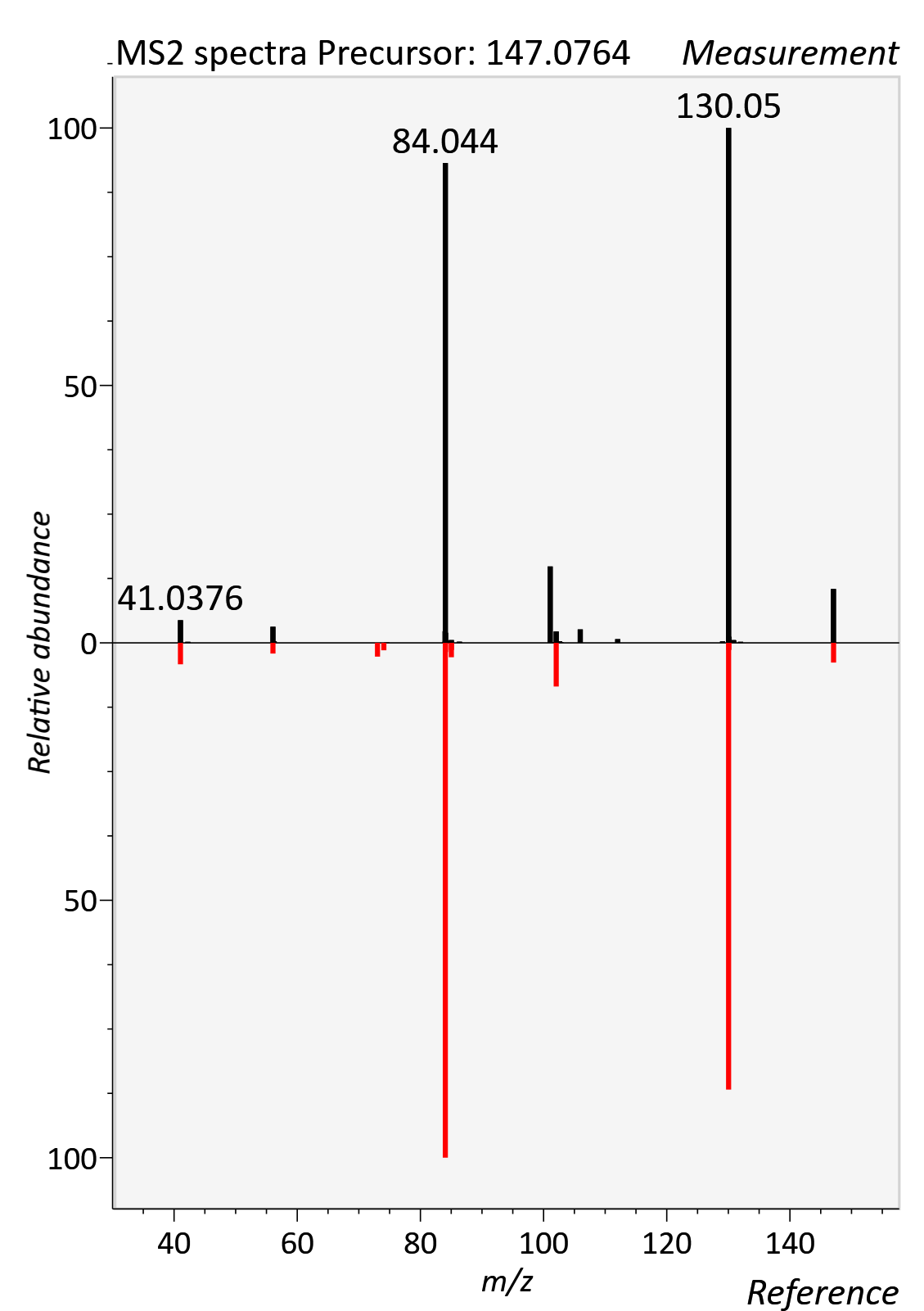


Glycine Betaine

RT std: 3.53min, RT experimental: 4.04min, RT Δ 0.49min


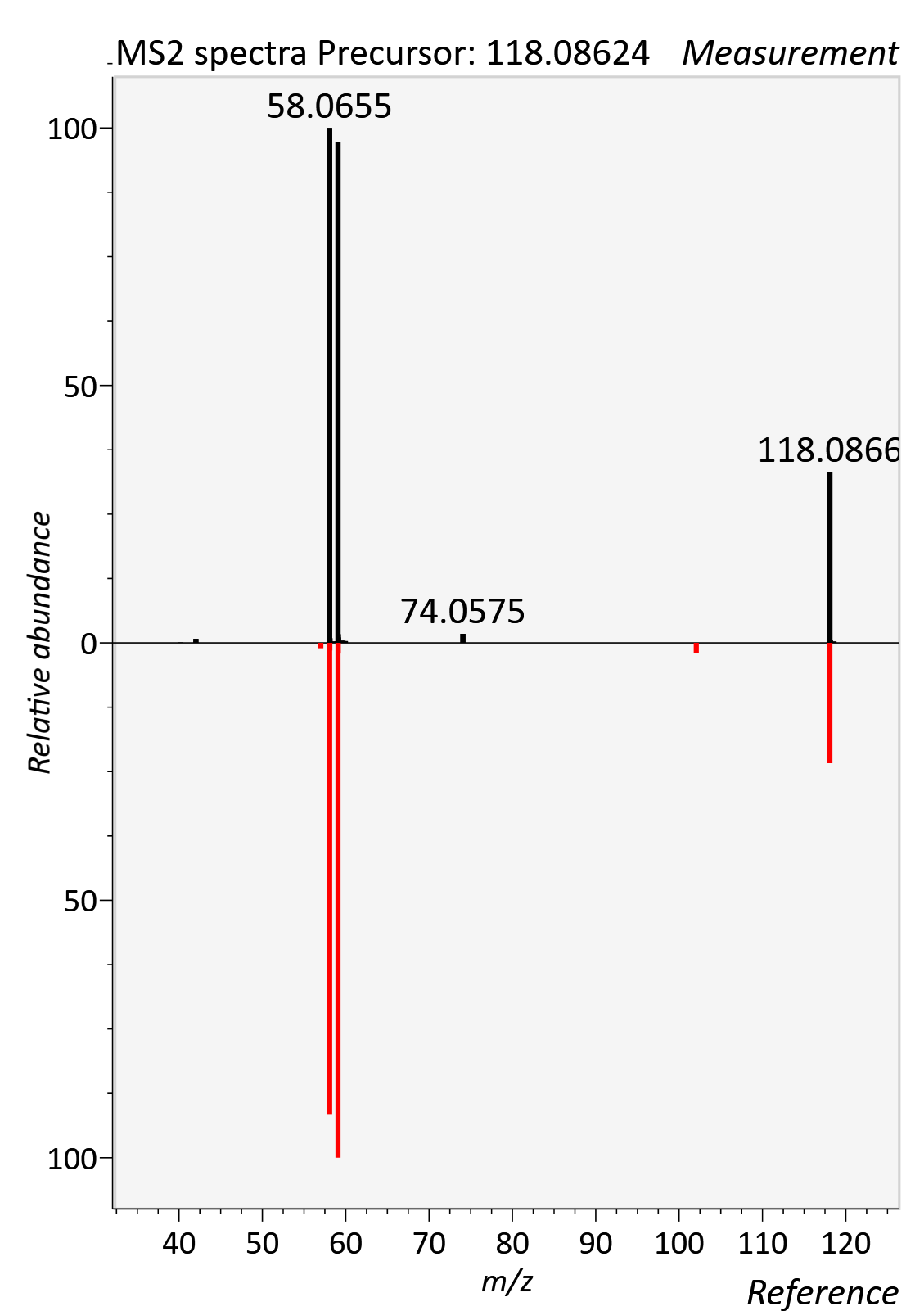


Glycochenodeoxycholic acid

RT std: 9.03min, RT experimental: 9.23min, RT Δ 0.20min


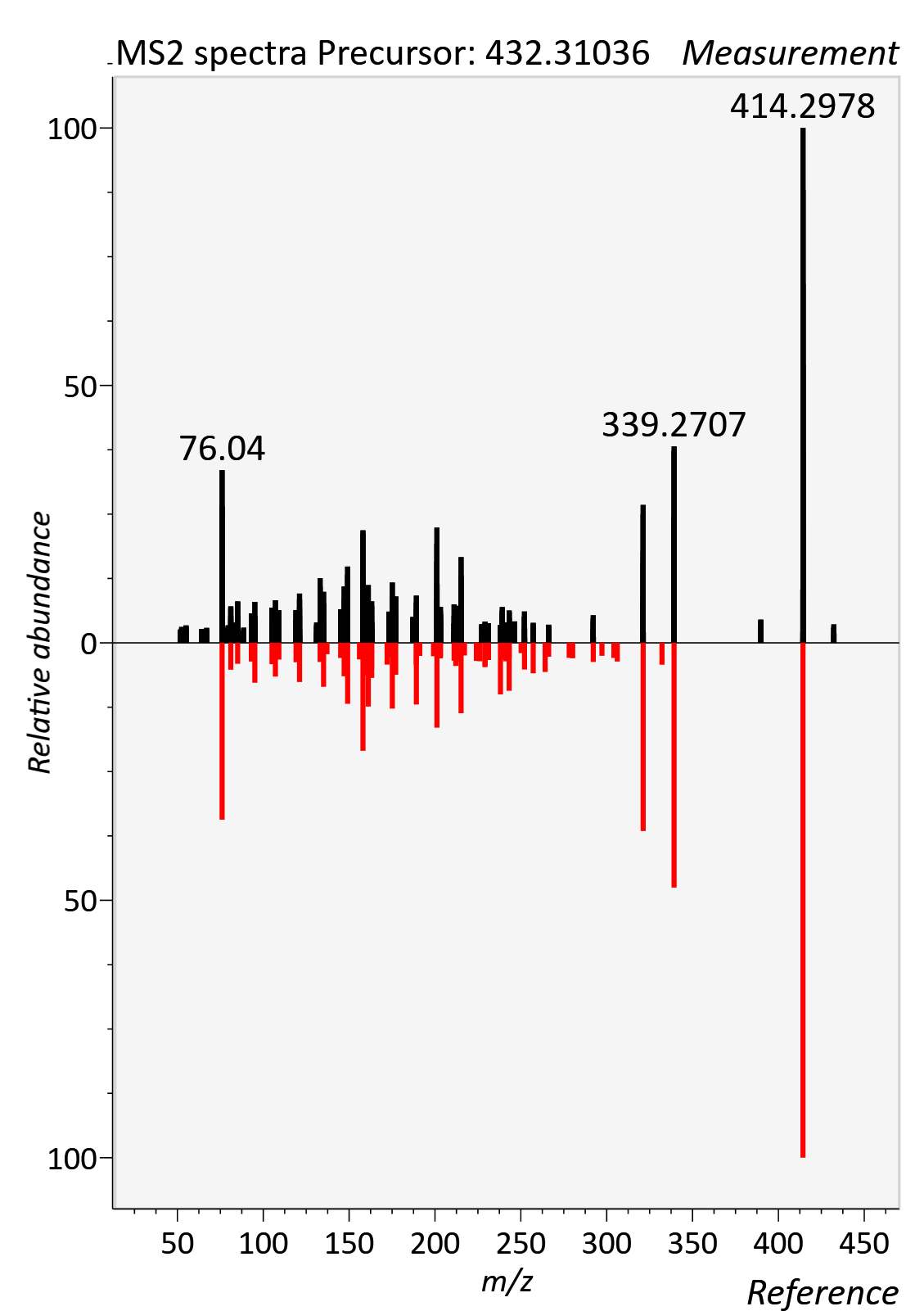


Glycocholic acid

RT std: 8.74min, RT experimental: 8.59min, RT Δ 0.15 min


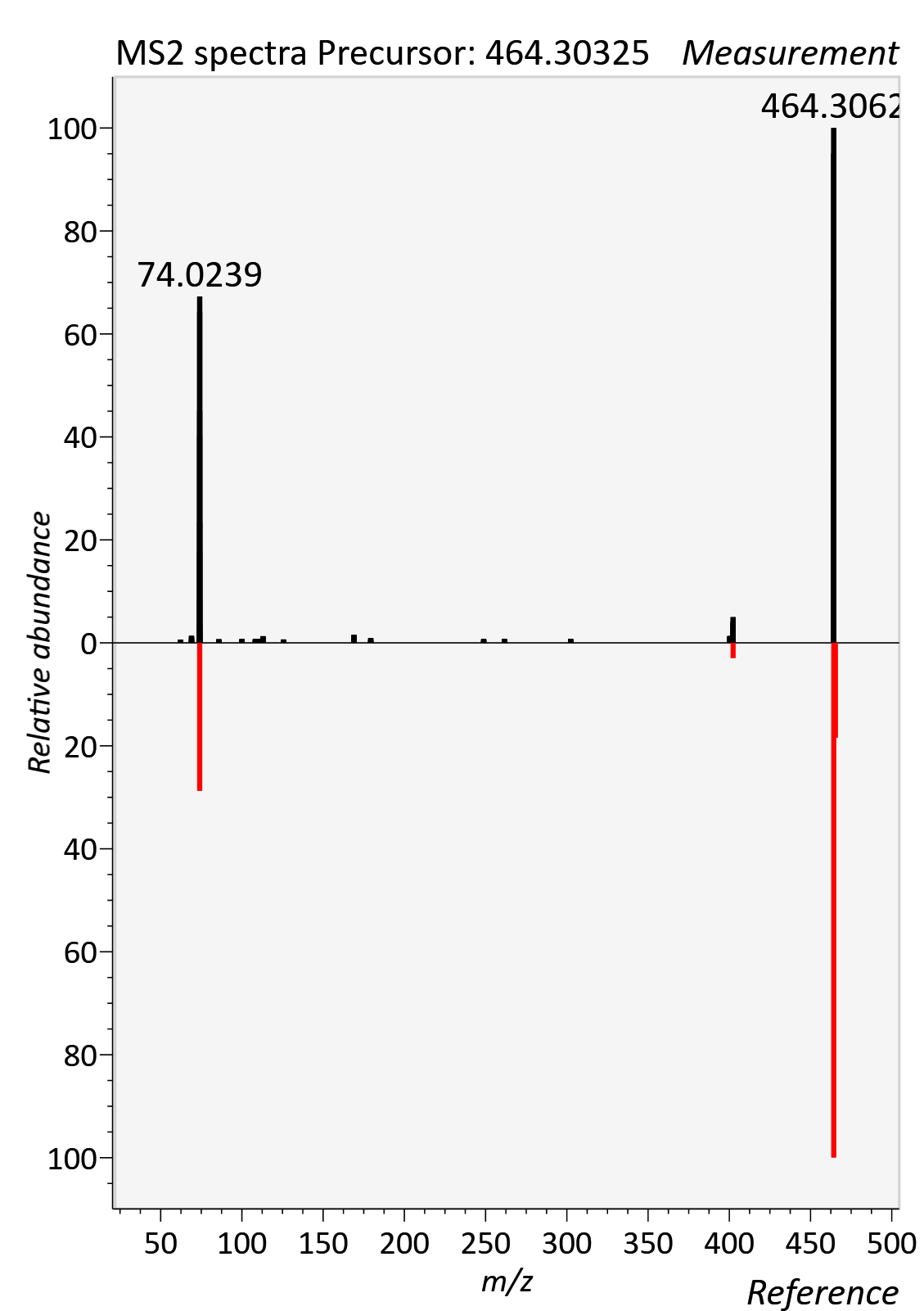


Glycohyodeoxycholic acid

RT std: 8.24min, RT experimental: 8.25min, RT Δ 0.01min


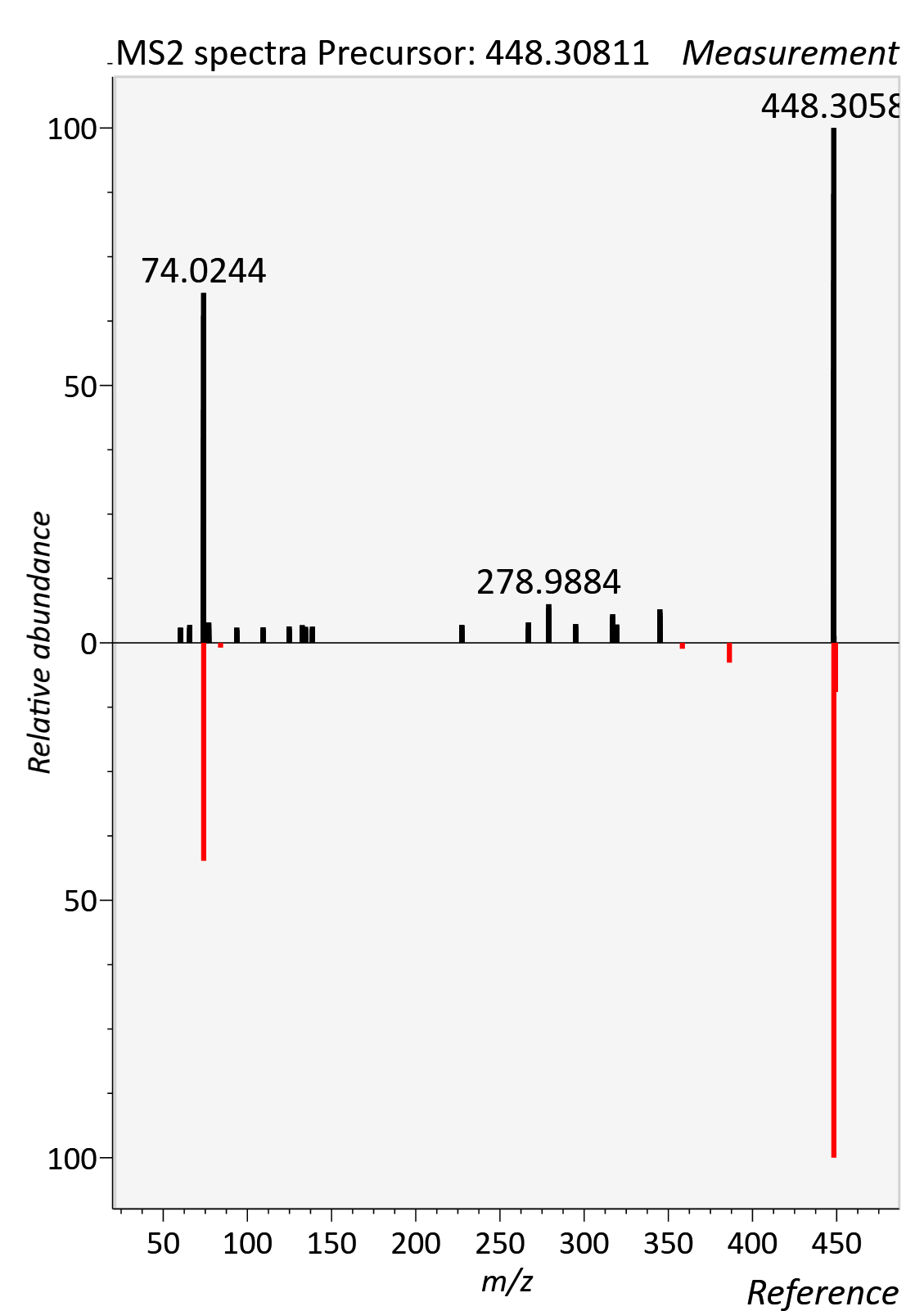


Glycolithocholic aicd

RT std: 9.33min, RT experimental: 9.58min, RT Δ 0.25min


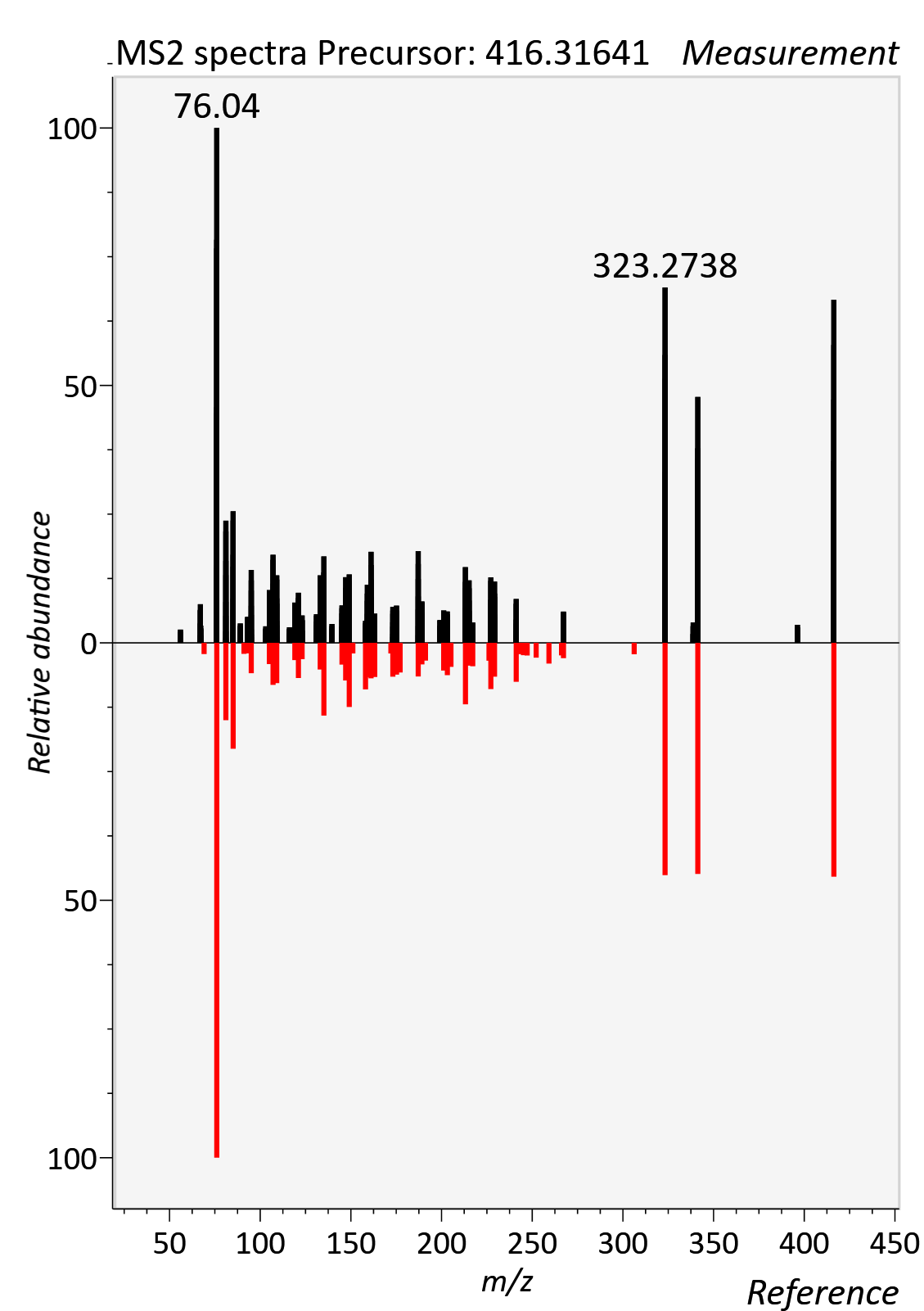


Hippuric acid

RT std: 3.30min, RT experimental: 3.54min, RT Δ 0.24min


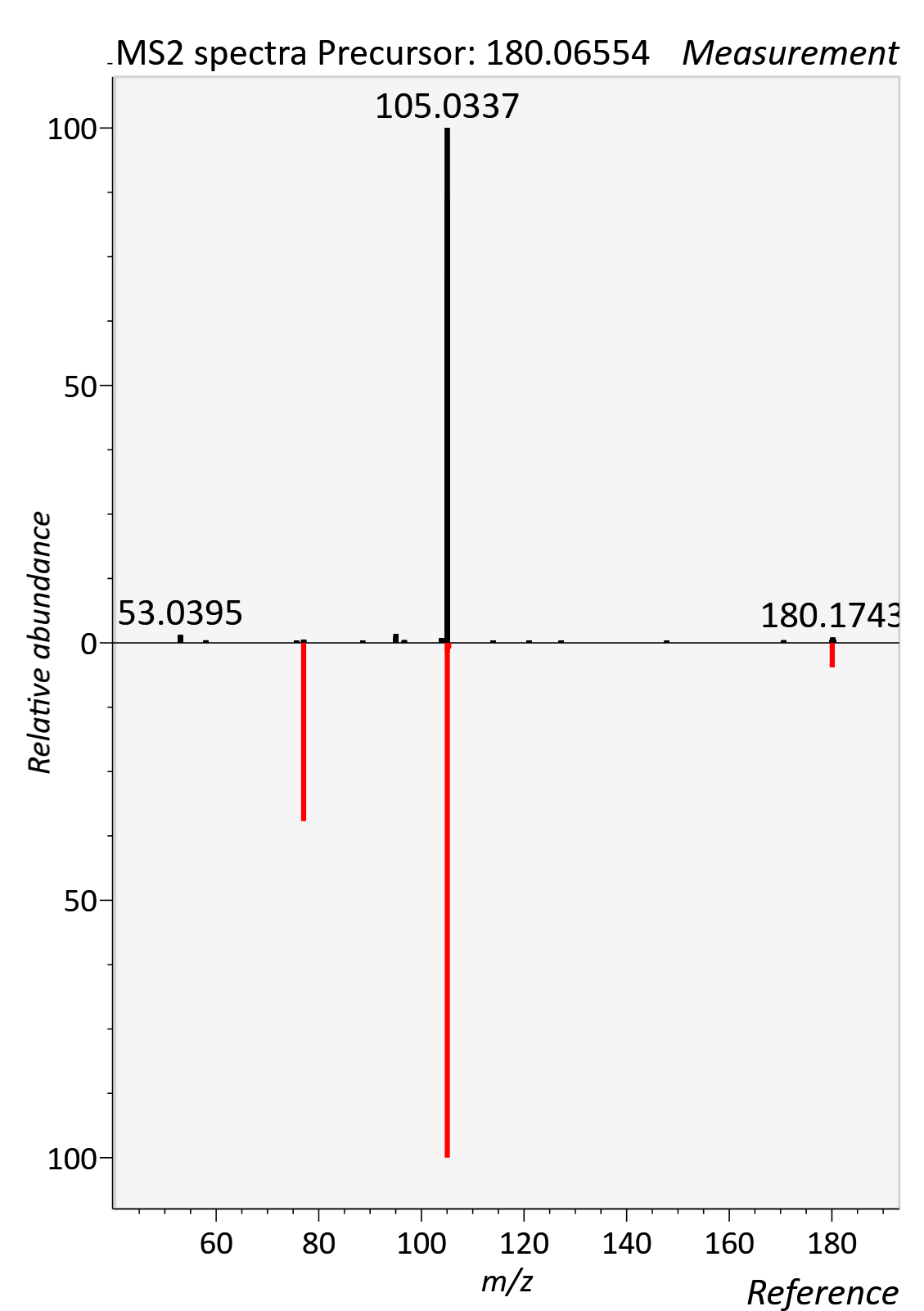


Hydroxyphenyllactic acid

RT std: 2.73min, RT experimental: 3.03min, RT Δ 0.30min


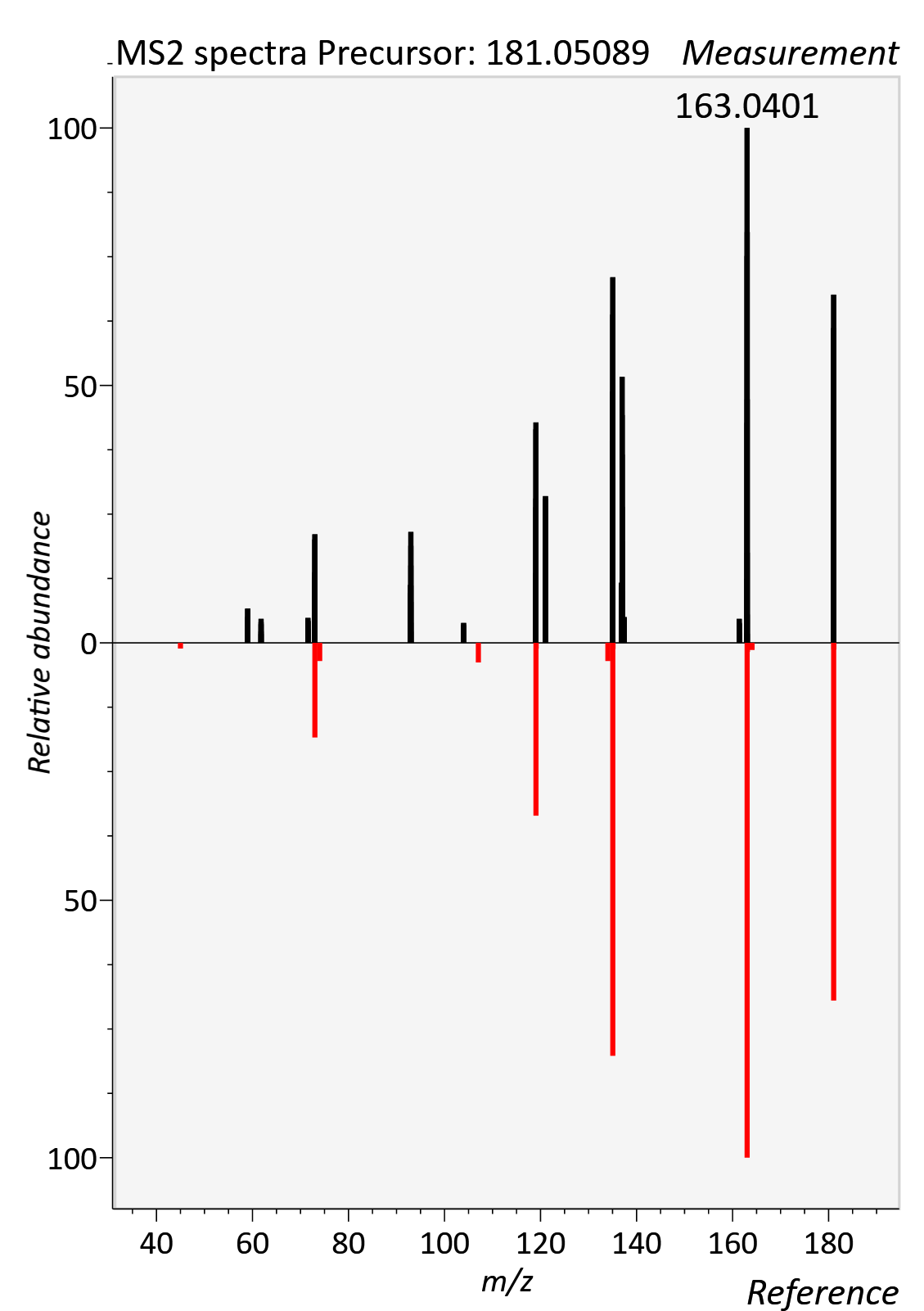


Hypoxanthine

RT std: 1.63min, RT experimental: 1.55min, RT Δ 0.08min


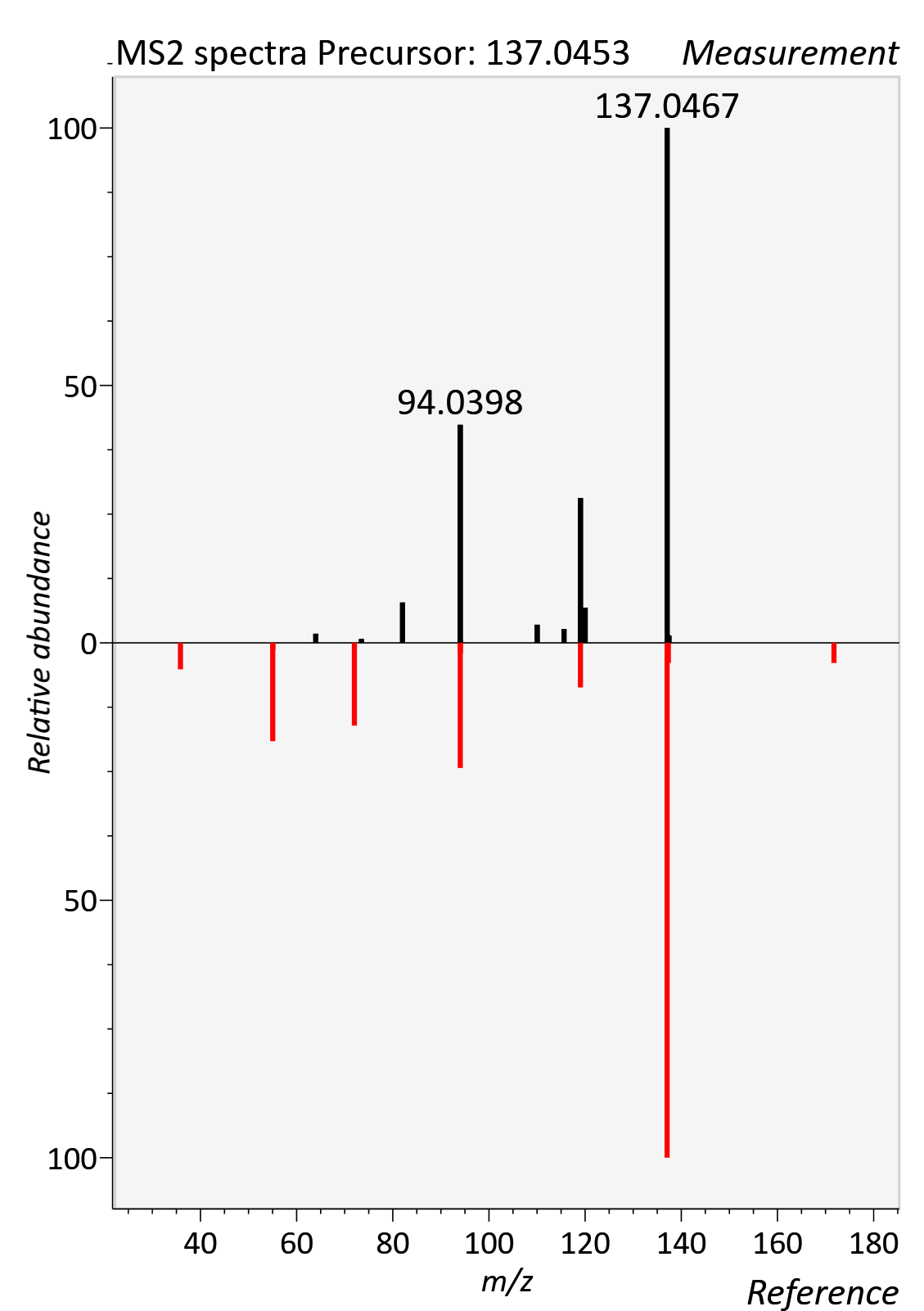


Indolelactic acid

RT std: 4.29min, RT experimental: 4.82min, RT Δ 0.53min


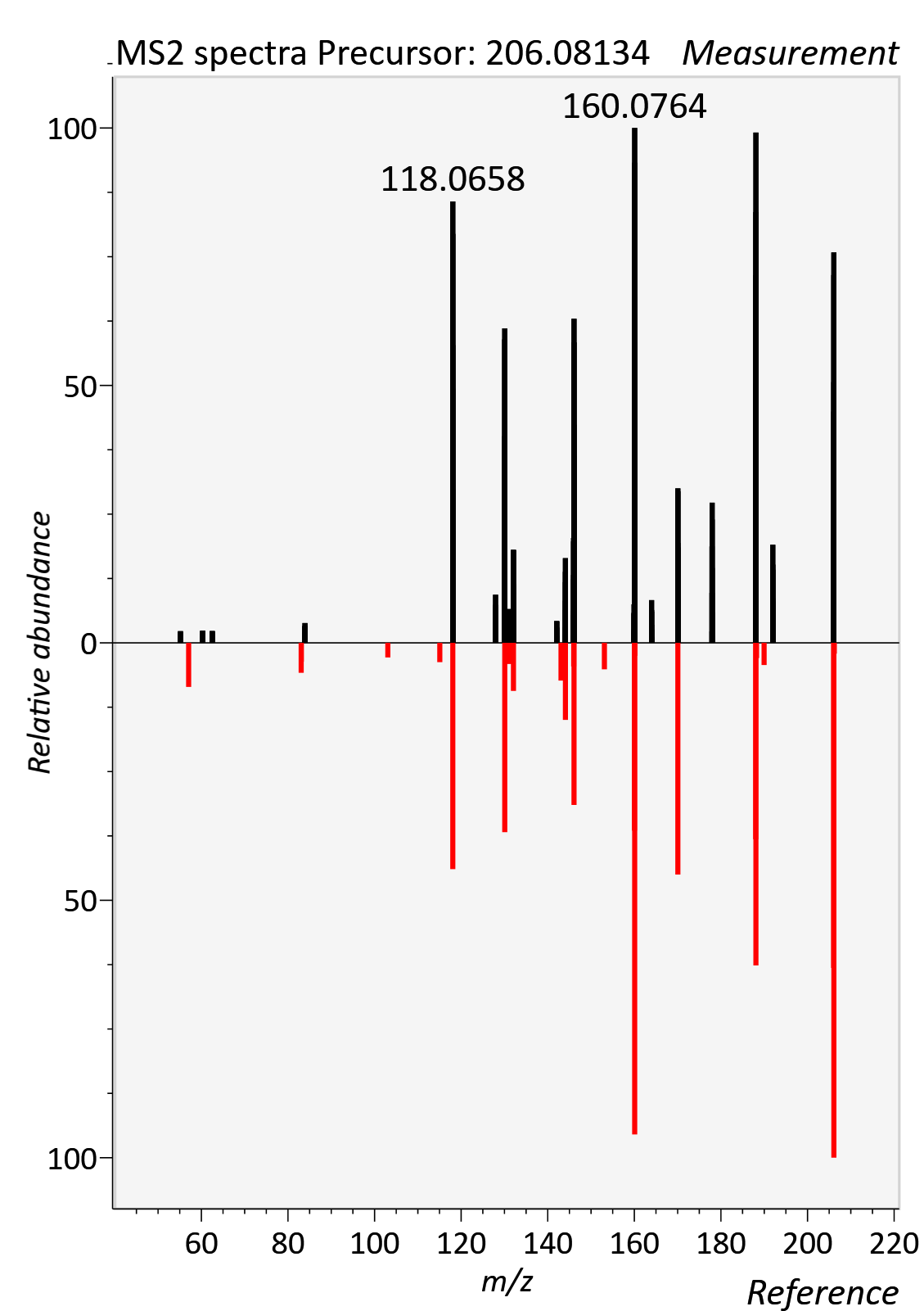


Indoxyl sulfate

RT std: 2.64min, RT experimental: 2.73min, RT Δ 0.09min


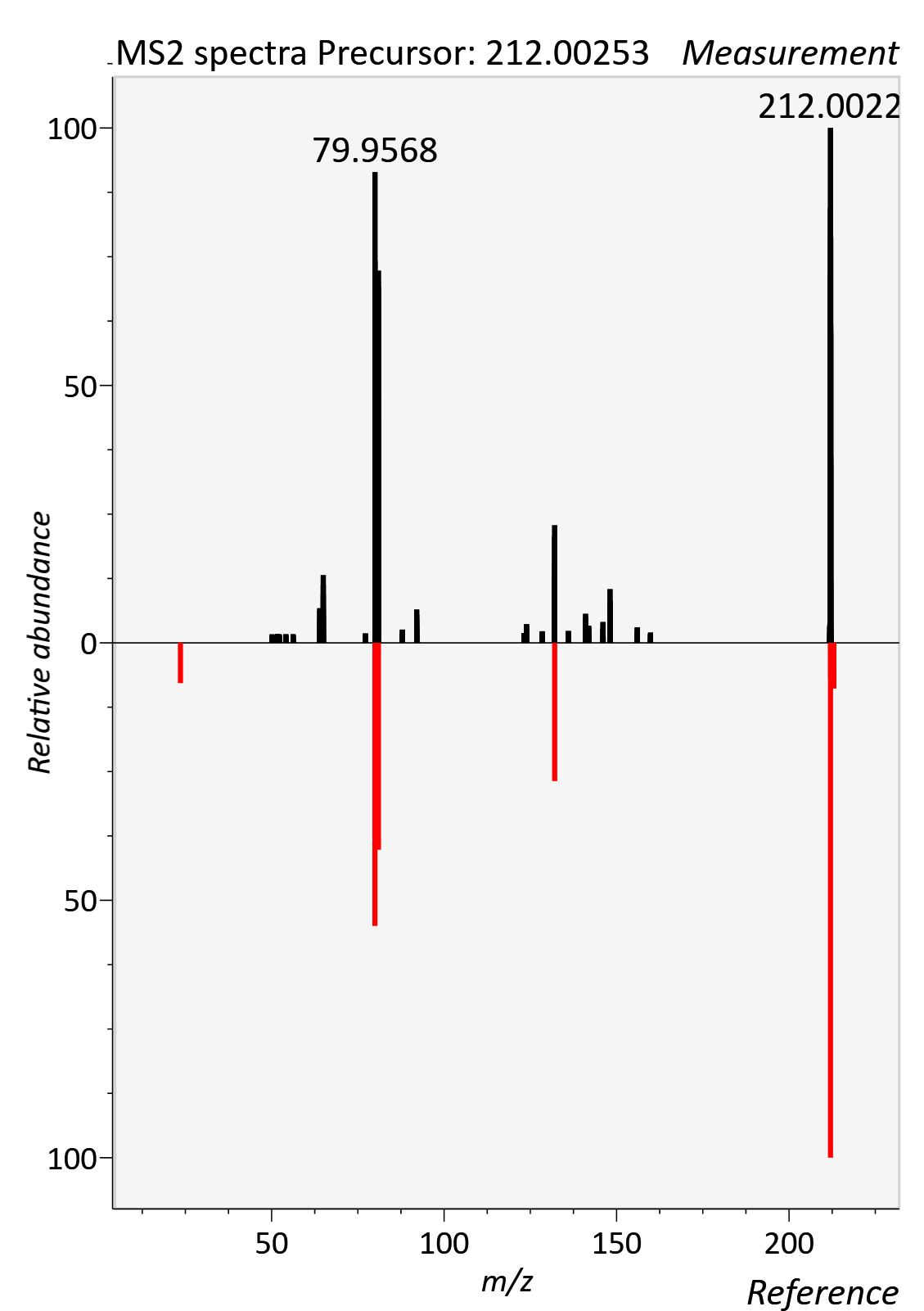


Isoleucine

RT std: 4.14min, RT experimental: 4.53min, RT Δ 0.39min


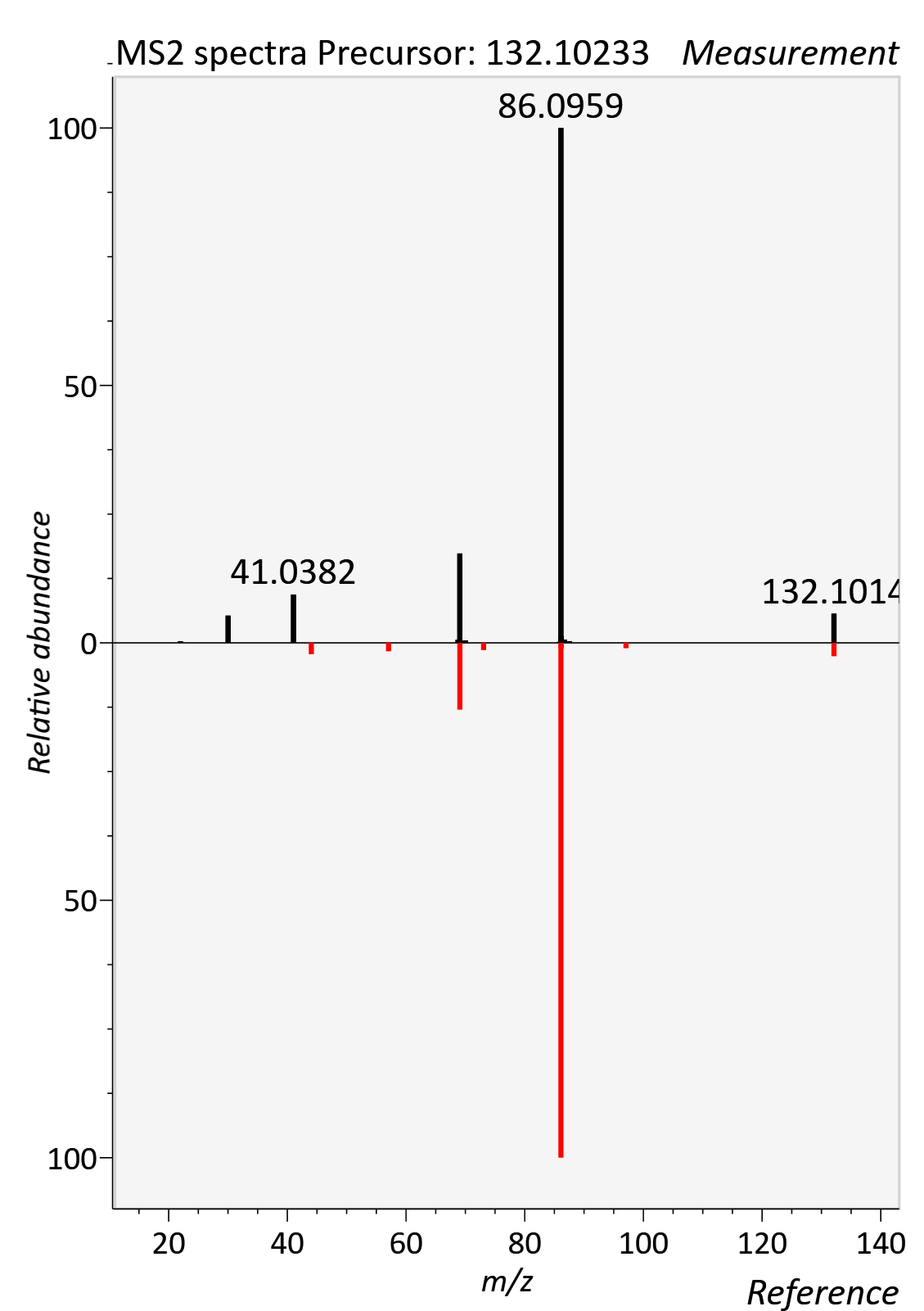


L-Arginine

RT std: 6.94min, RT experimental: 7.11min, RT Δ 0.17min


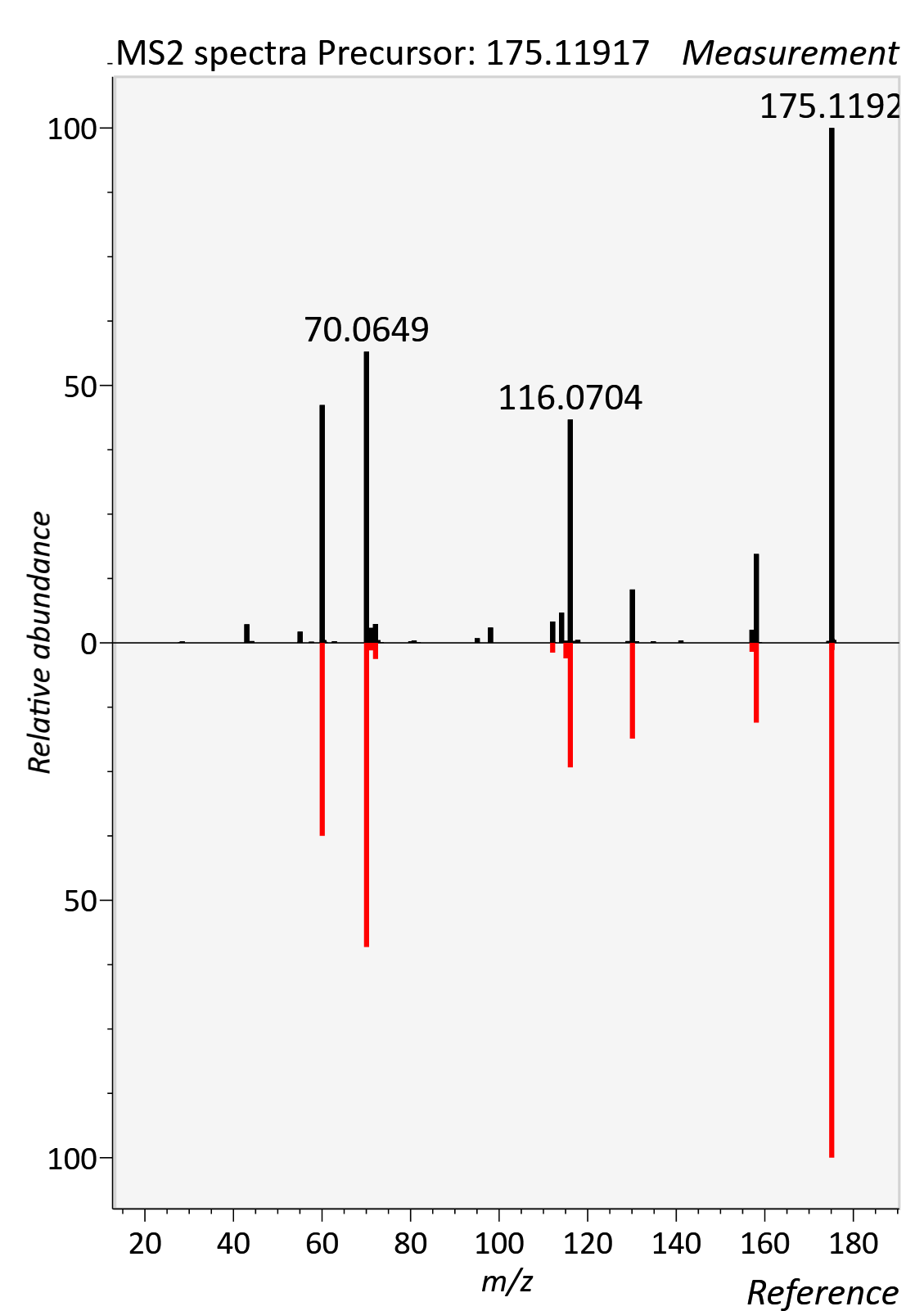


L-Carnitine

RT std: 4.77min, RT experimental: 5.10min, RT Δ 0.37min


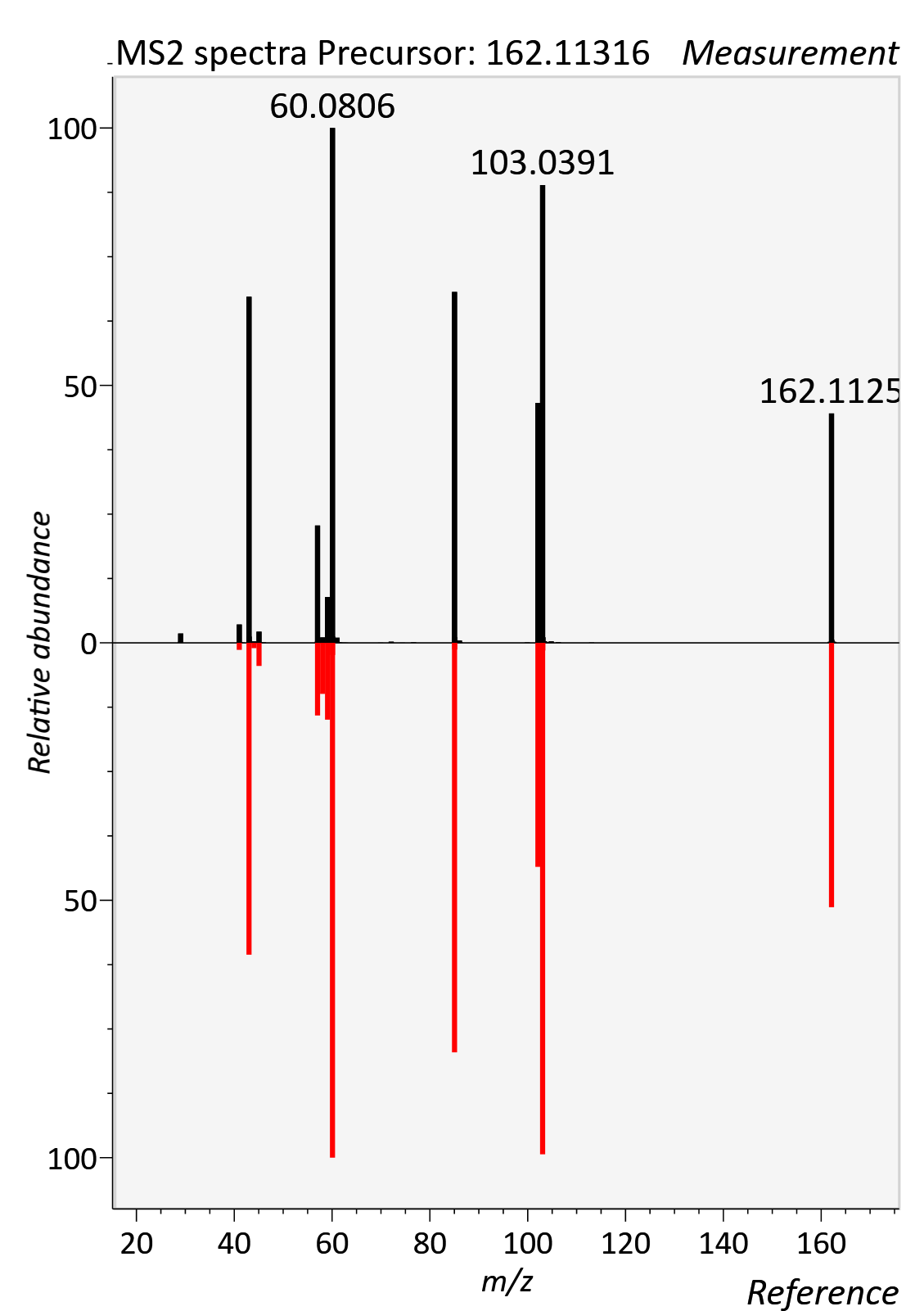


Leucine

RT std: 3.80, RT experimental: 4.28min, RT Δ 0.48min


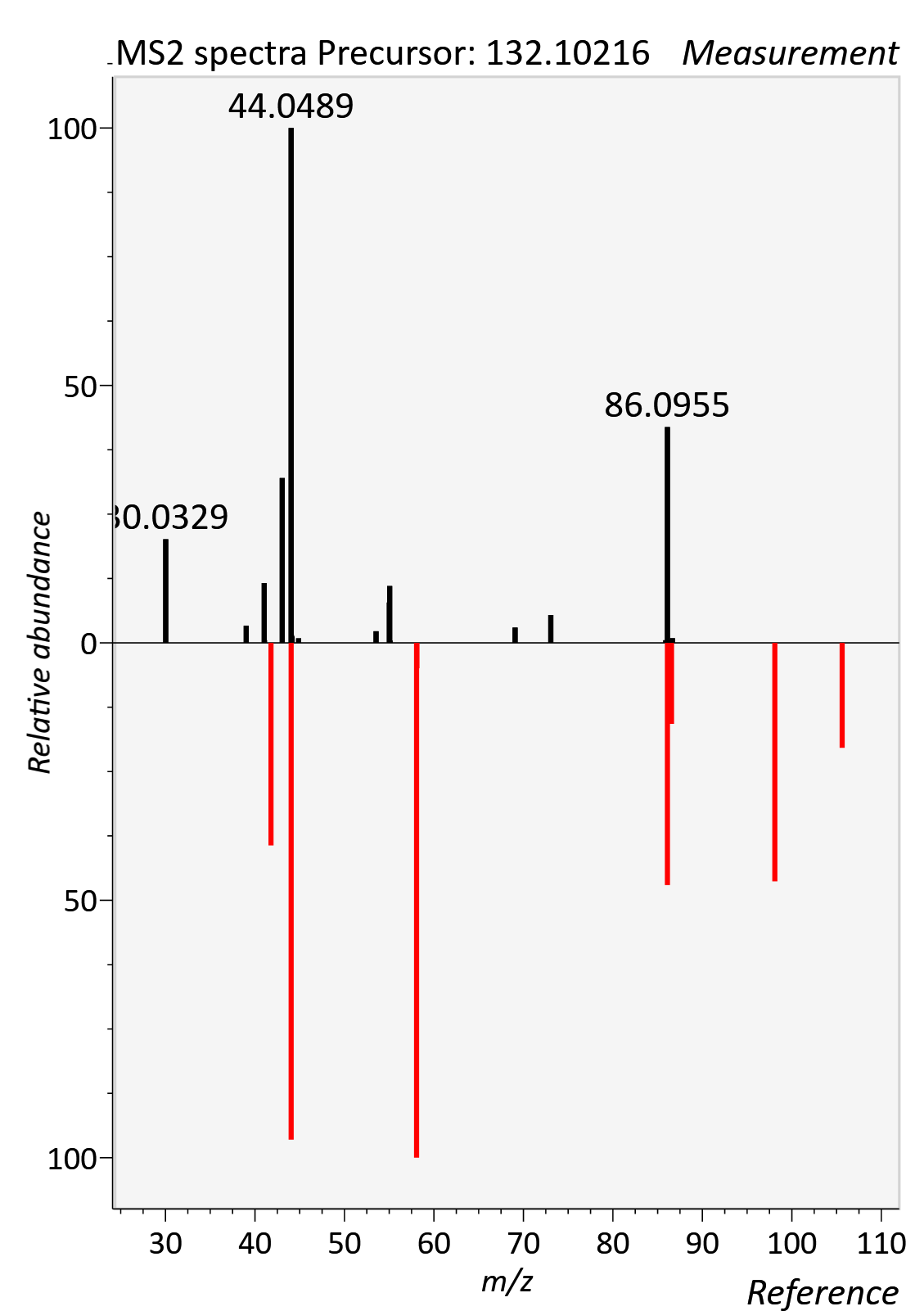


LysoPC 15:0_0:0

RT std: 10.04min, RT experimental: 10.21min, RT Δ 0.16min


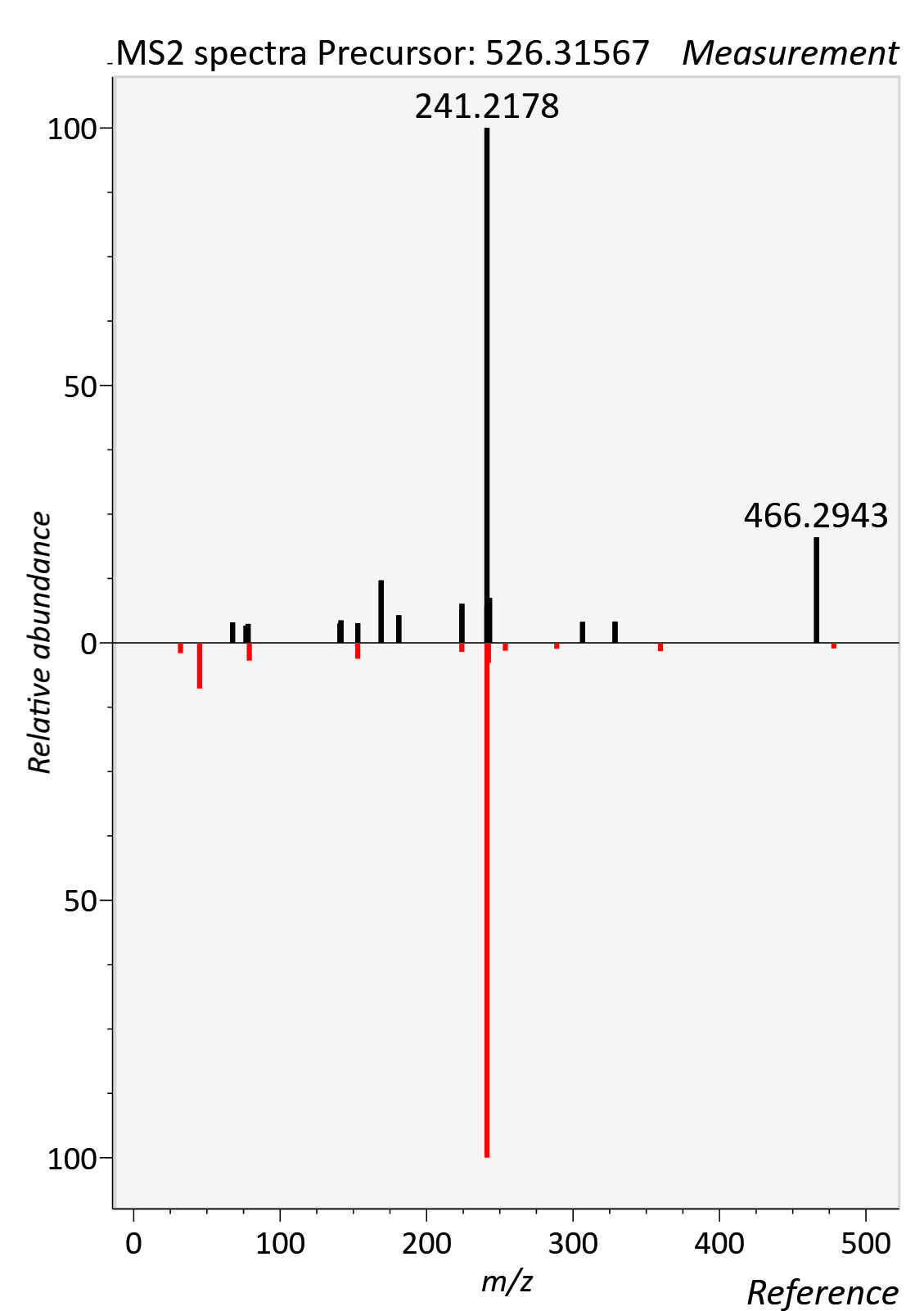


LysoPC 17:0_0:0

RT std: 10.42min, RT experimental: 10.70min, RT Δ 0.28min


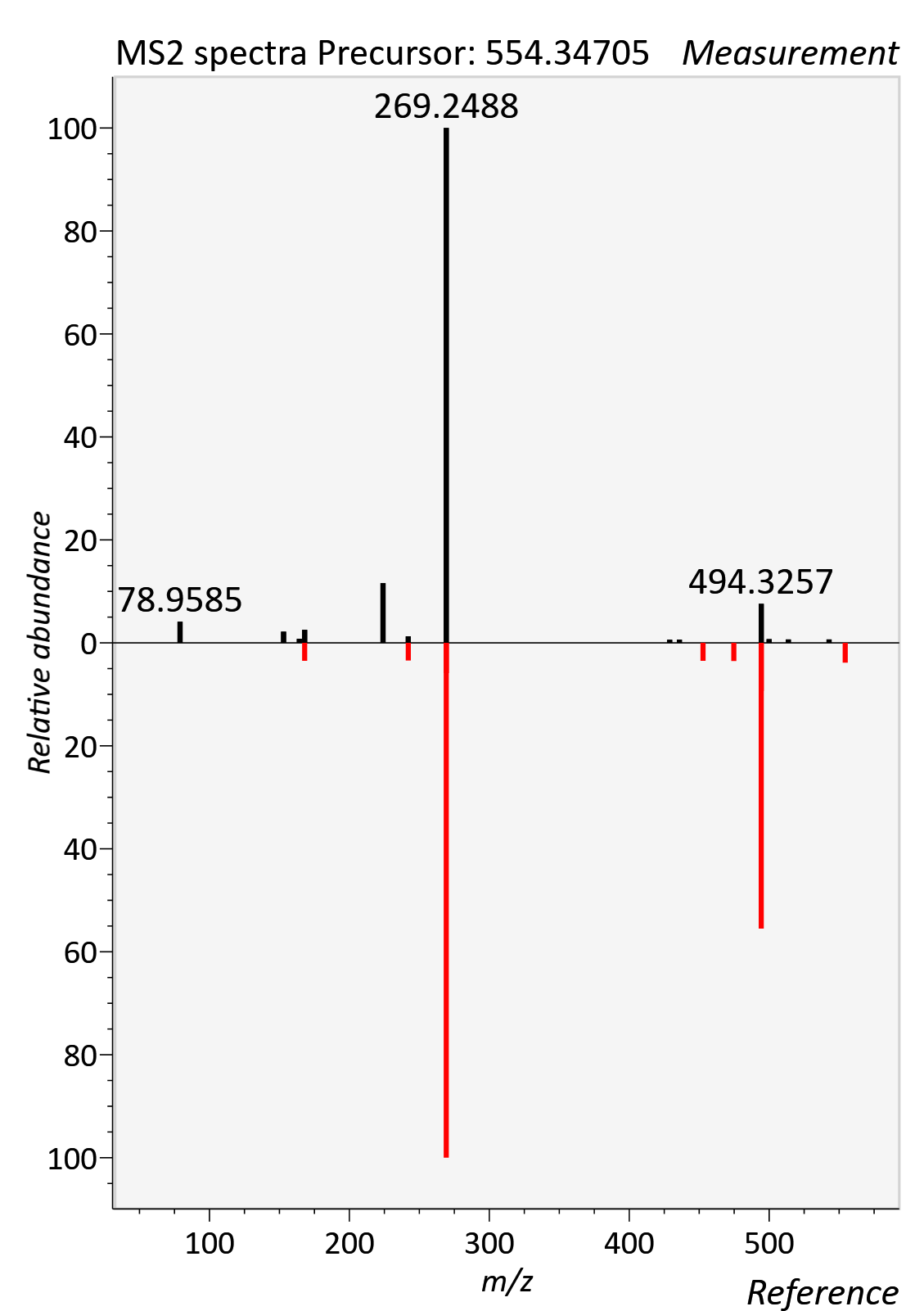


Kynurenine

RT std: 1.73min, RT experimental: 1.90min, RT Δ 0.17min


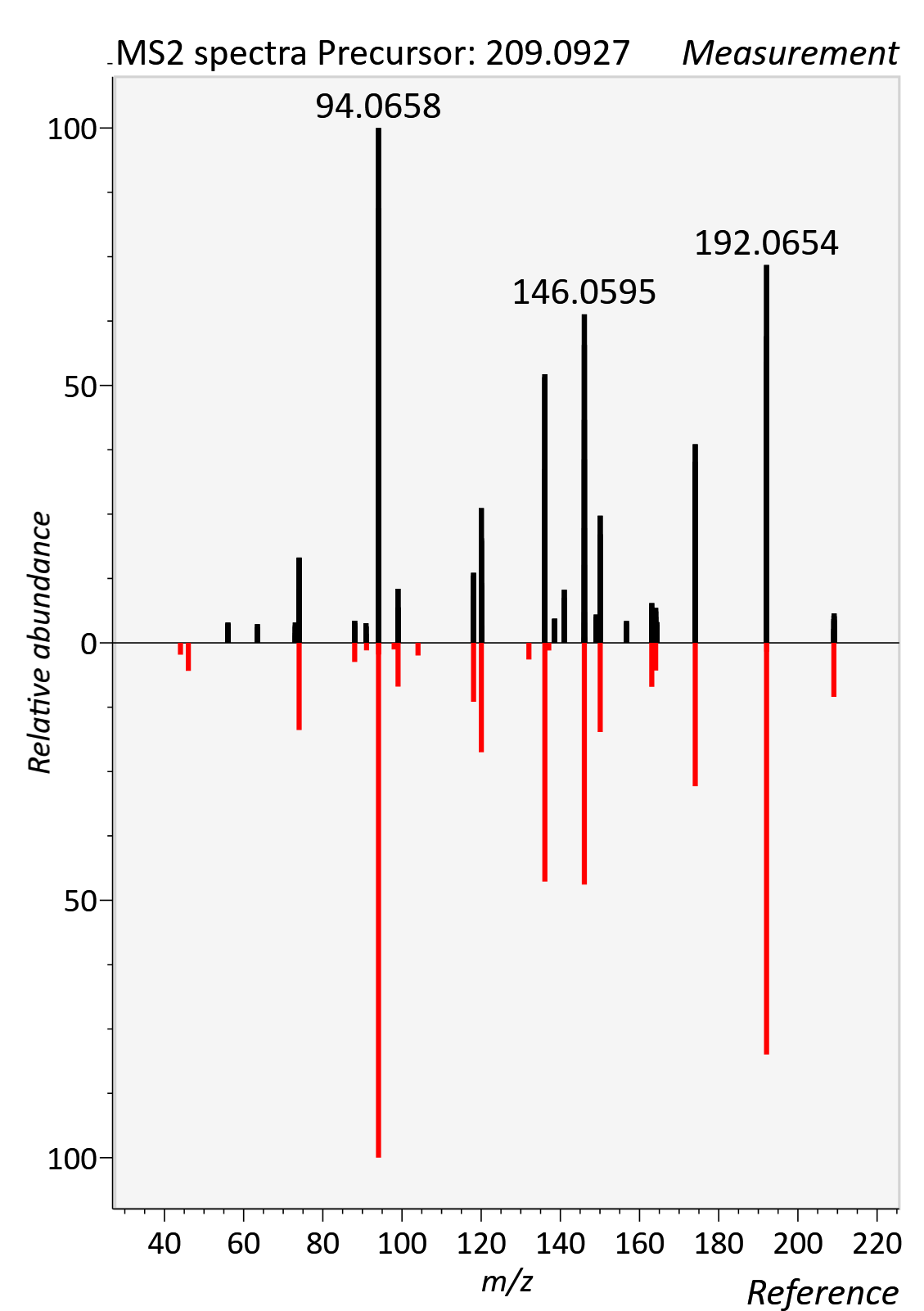


Ornithine

RT std: 7.17min, RT experimental: 7.30min, RT Δ 0.13min


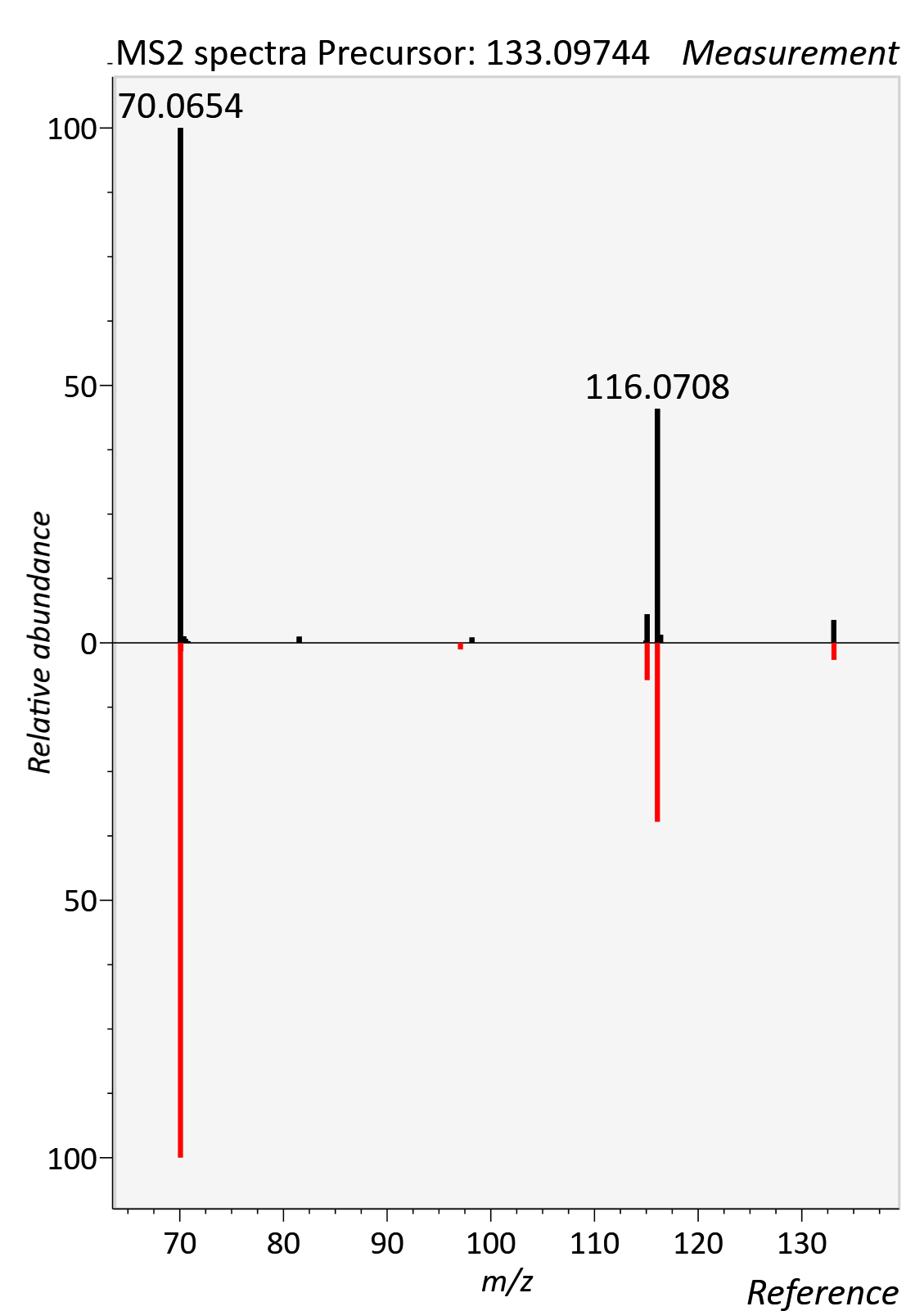


Paraxanthine

RT std: 2.93min, RT experimental: 2.61min, RT Δ 0.32min


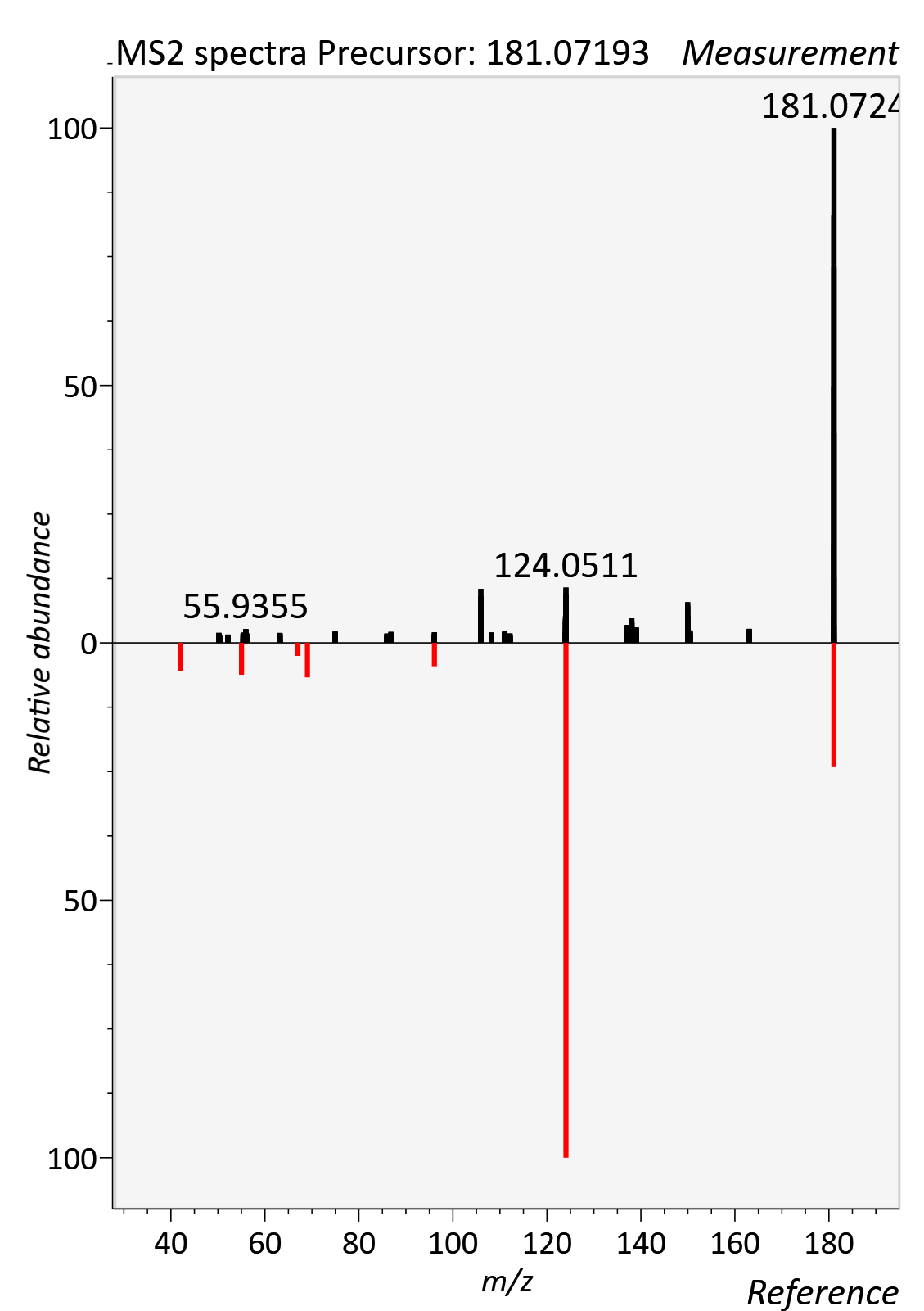


Phenylalanine

RT std: 3.71min, RT experimental: 4.22min, RT Δ 0.51min


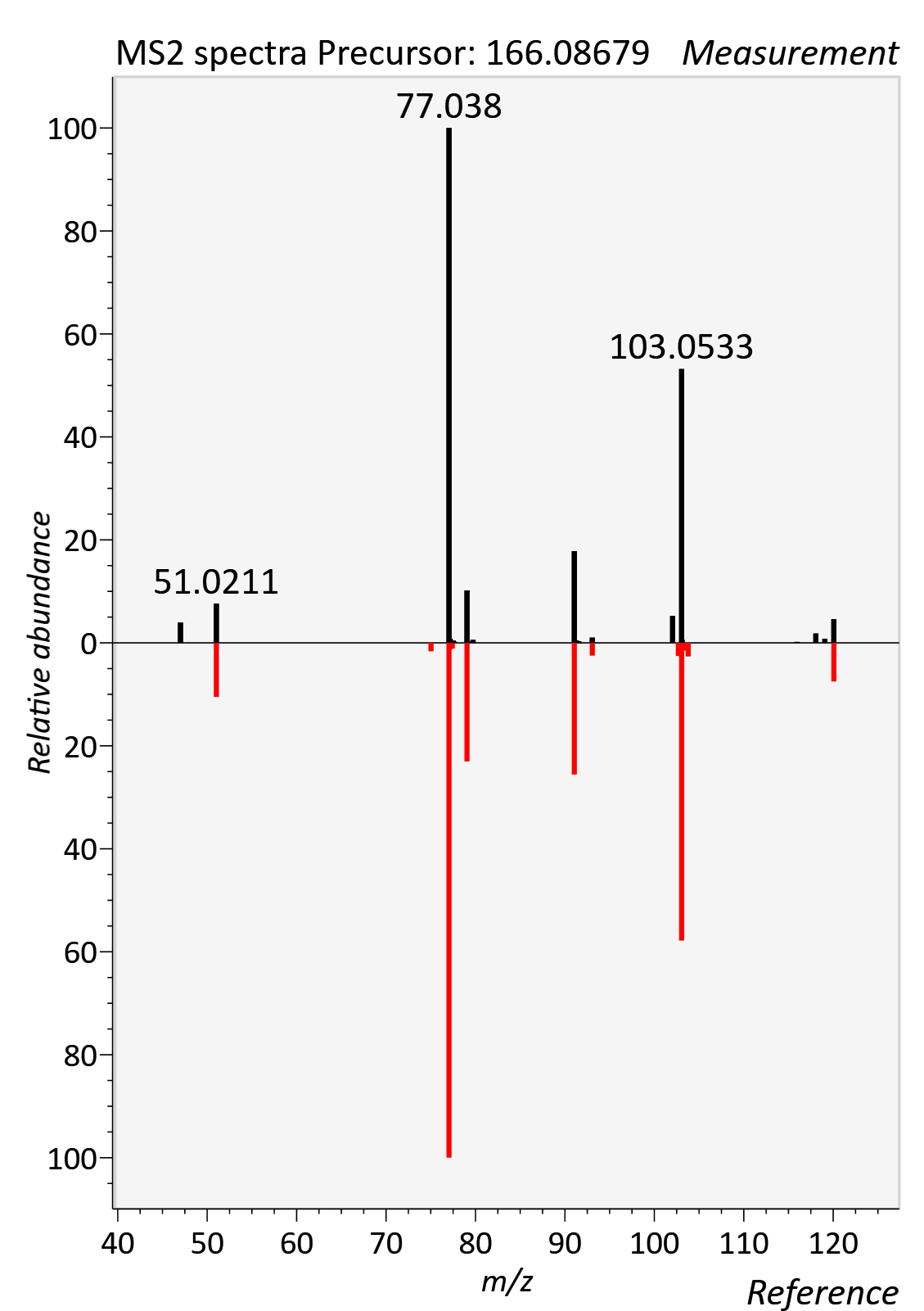


Pipecolic acid

RT std: 4.96min, RT experimental: 4.19min, RT Δ 0.75min


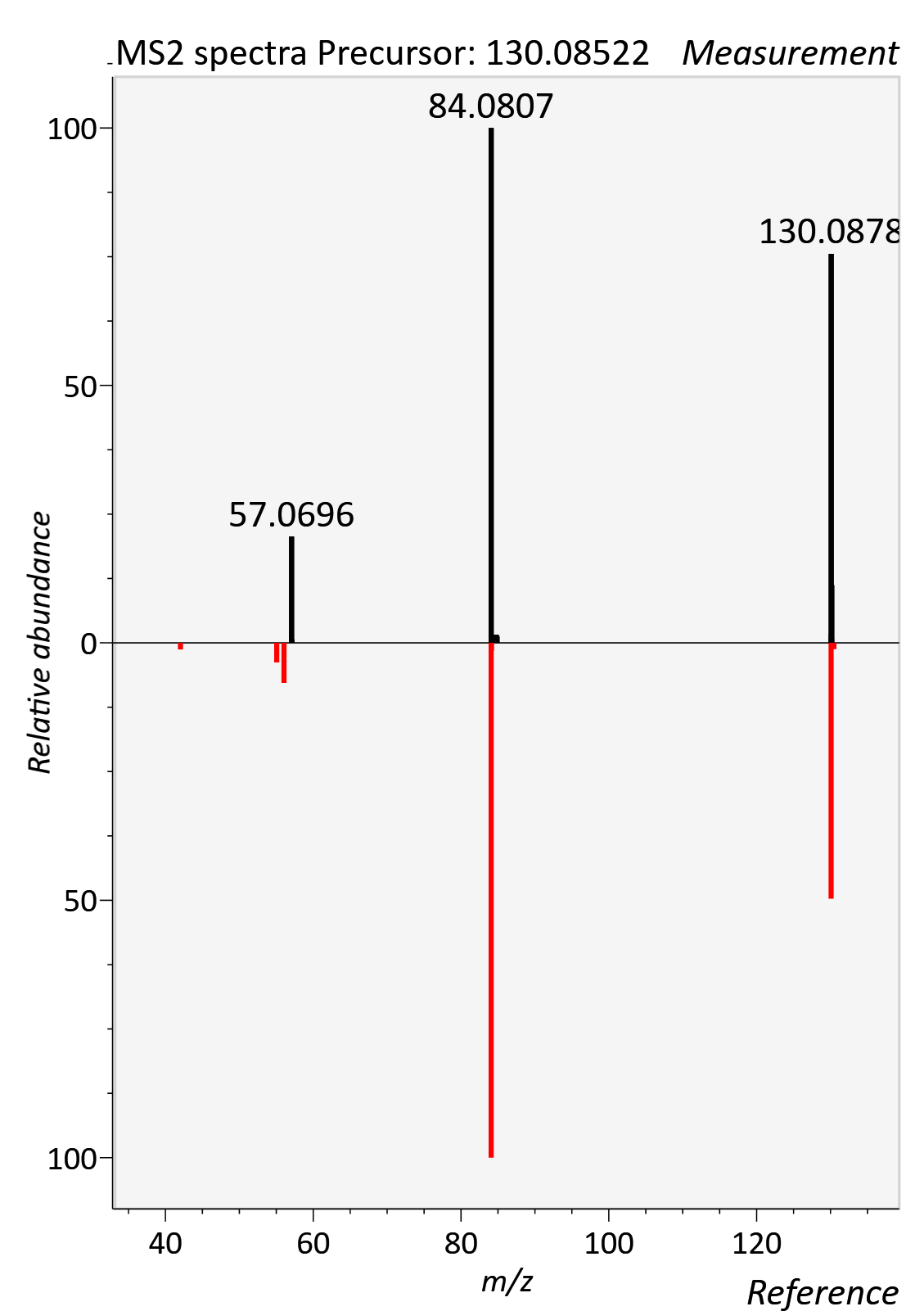


Proline Betaine

RT std: 3.46min, RT experimental: 3.85min, RT Δ 0.39min


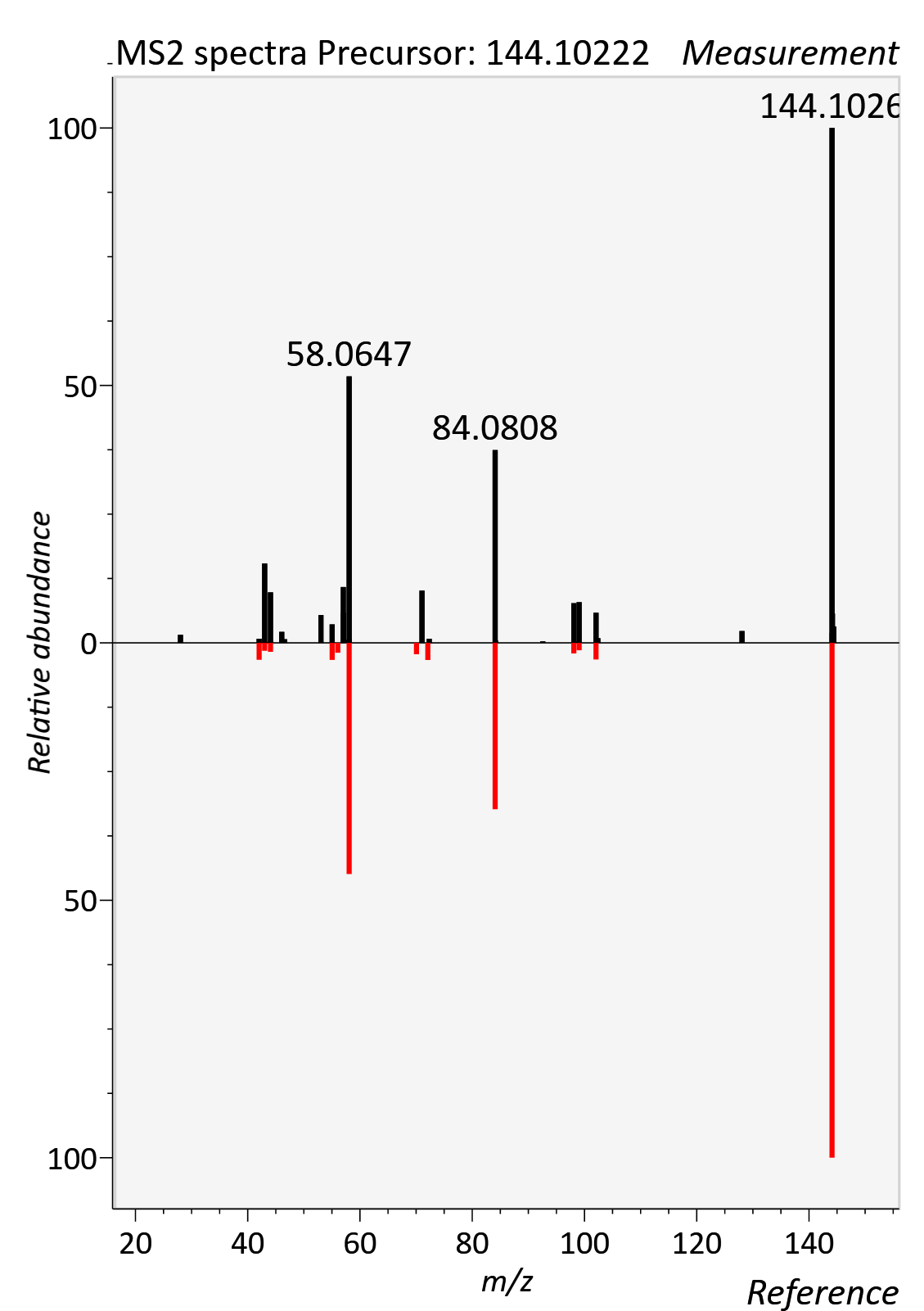


Theobromine

RT std: 2.40min, RT experimental: 2.07min, RT Δ 0.33min


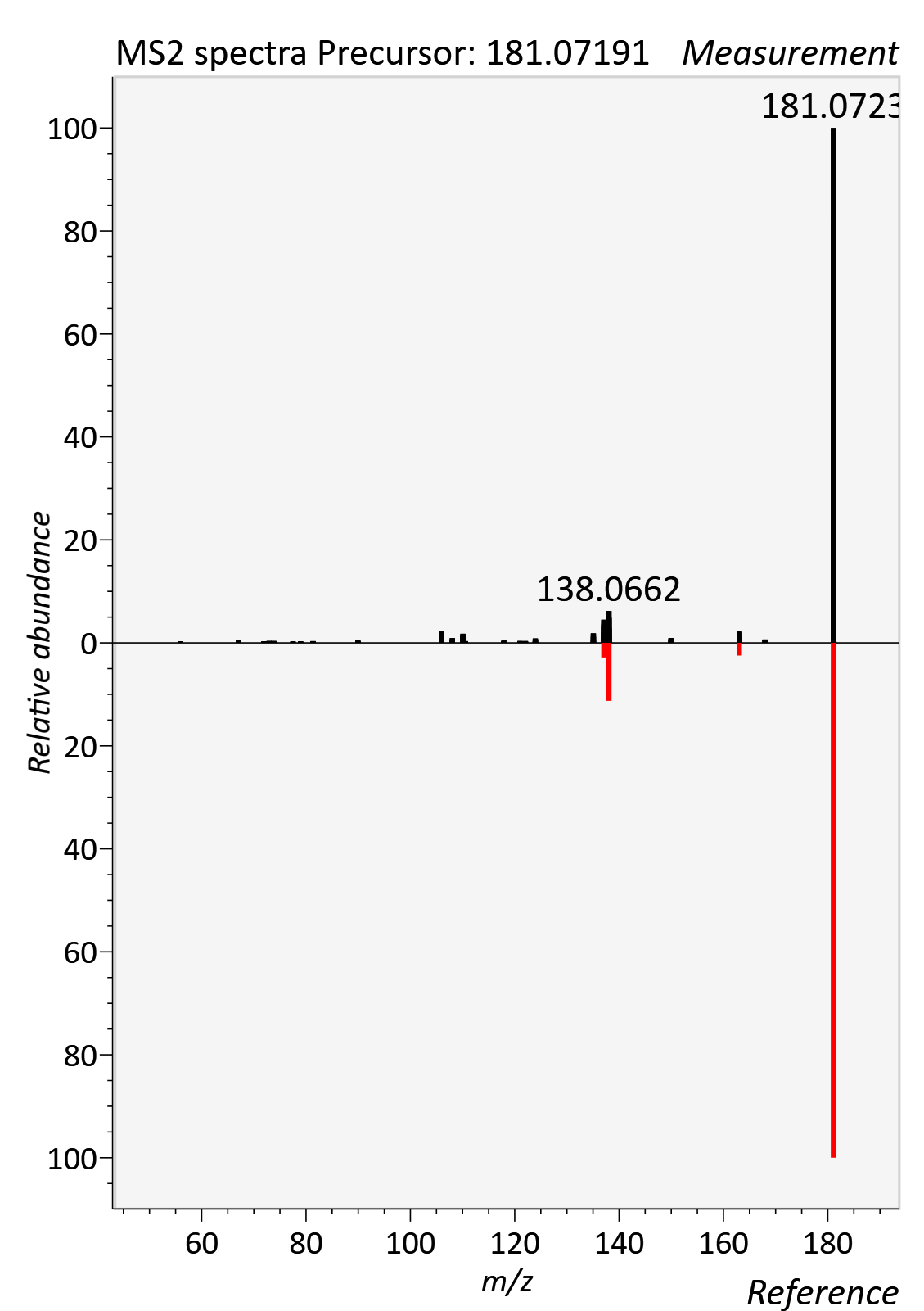


Theophylline

RT std: 2.93min, RT experimental: 3.06min, RT Δ 0.13min


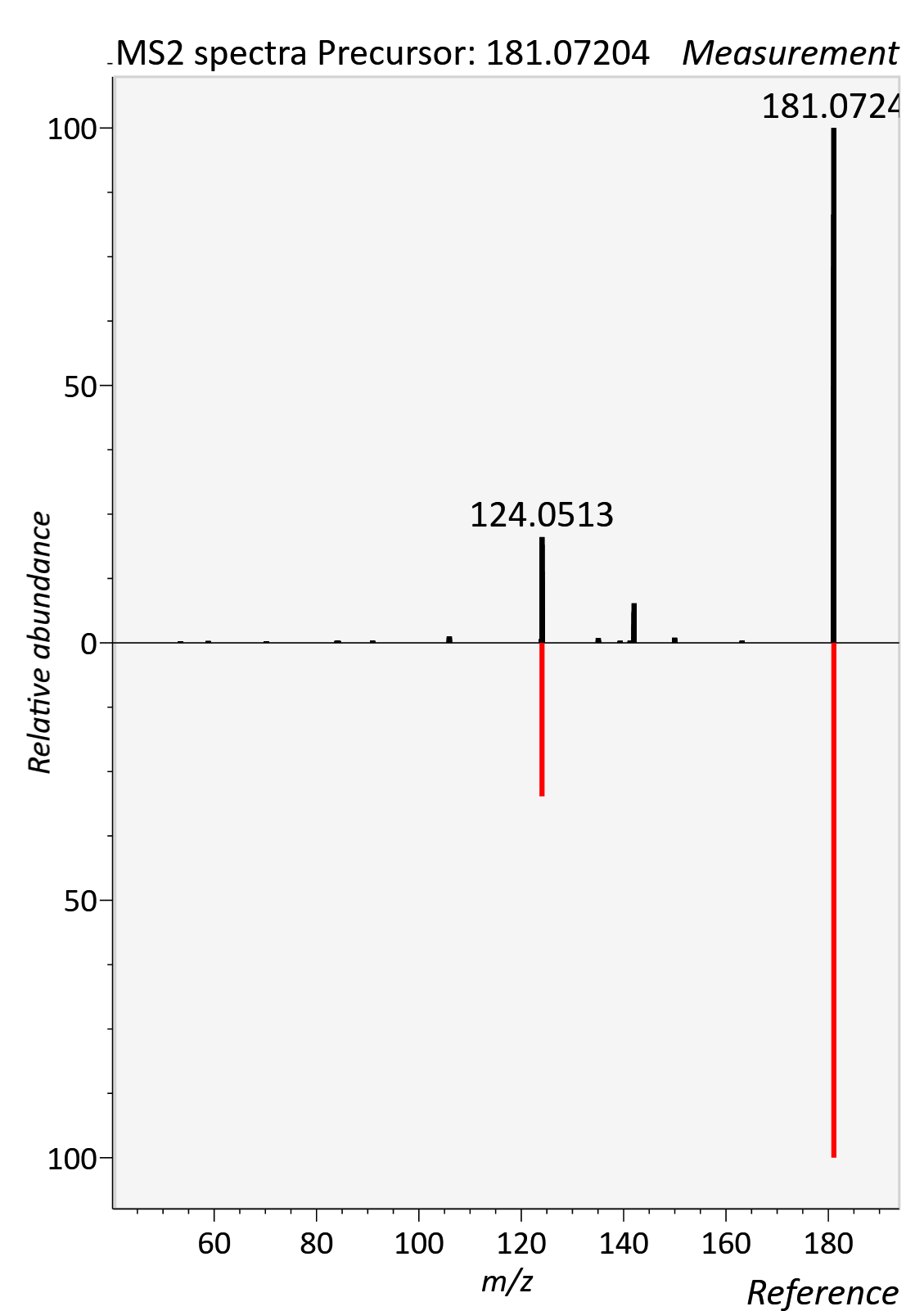


Threonine

RT std: 5.78min, RT experimental: 5.95min, RT Δ 0.17min


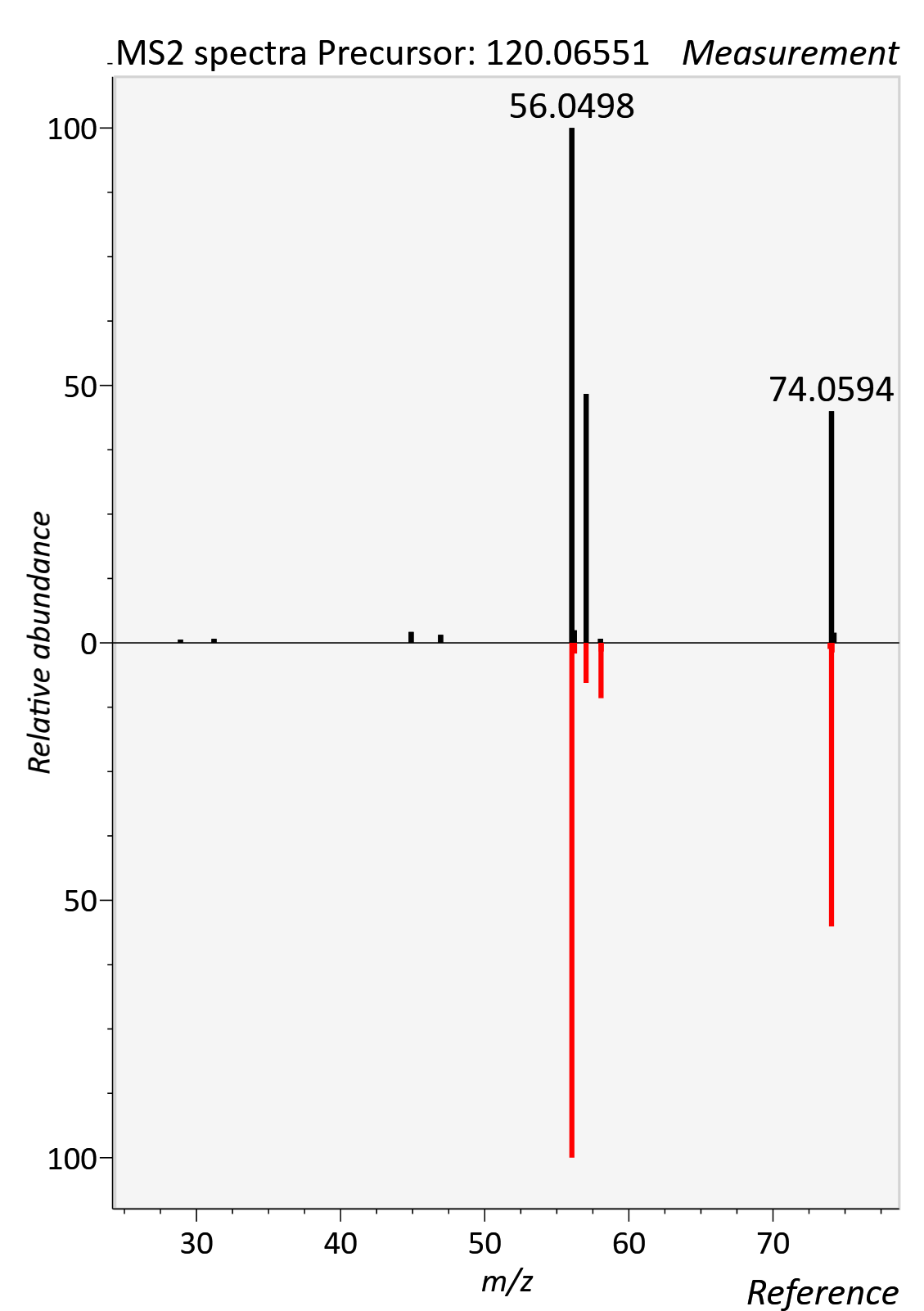


Trigonelline

RT std: 4.03min, RT experimental: 4.52min, RT Δ 0.49min


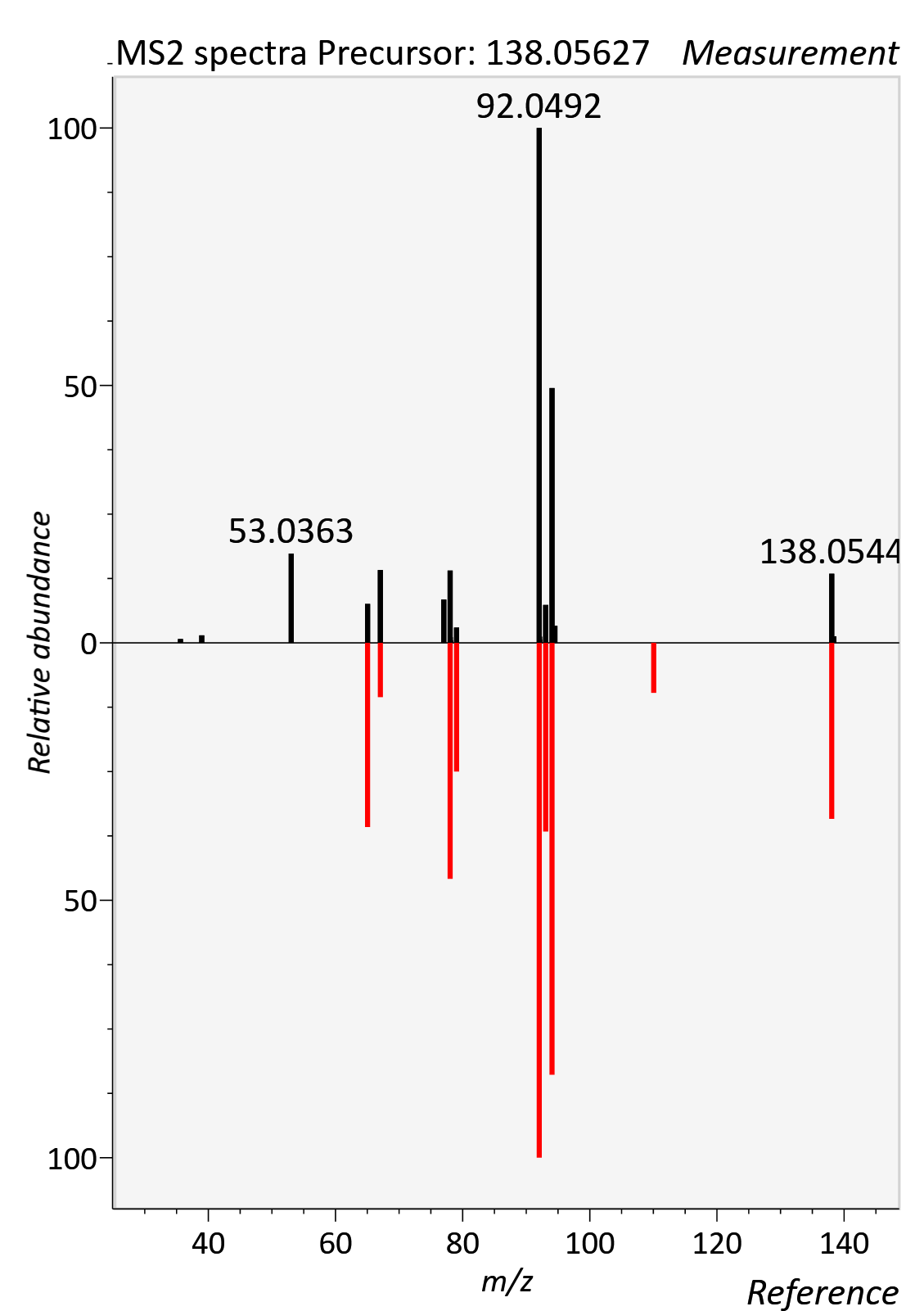


Tryptophan

RT std: 2.76min, RT experimental: 2.43min, RT Δ 0.33min


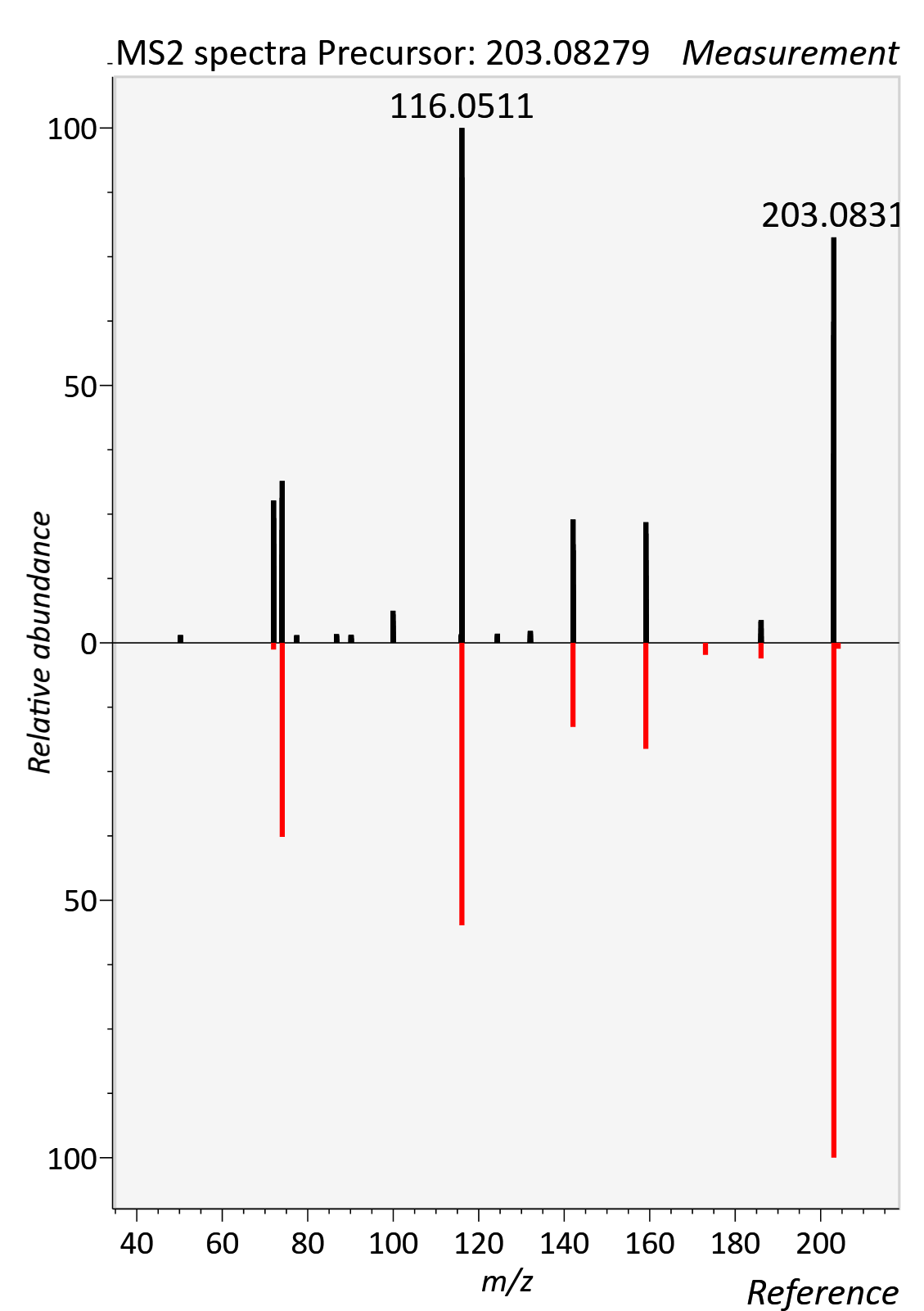


Tyrosine

RT std: 5.01min, RT experimental: 5.30min, RT Δ 0.29min


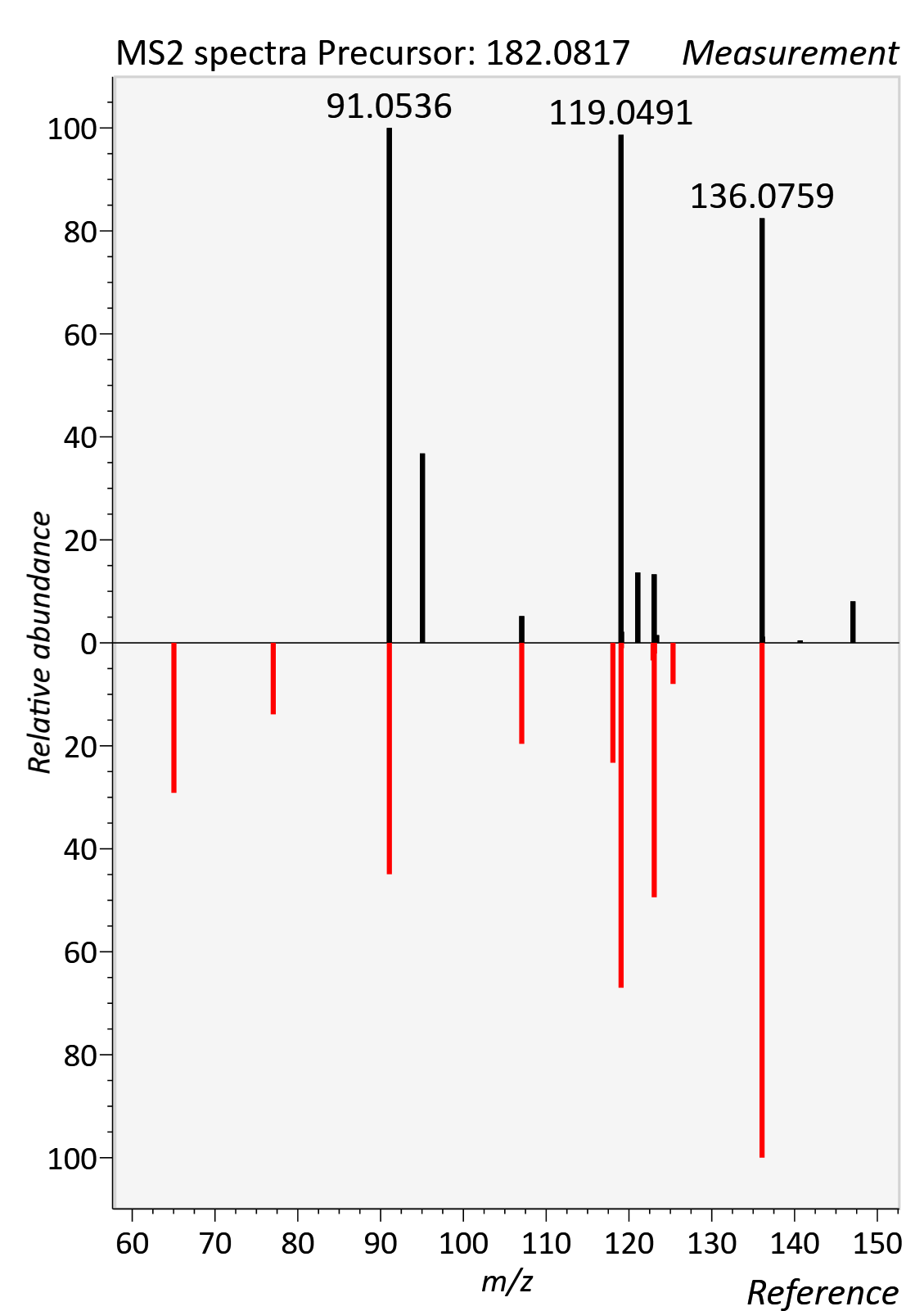

Supplement: Supplementary file 3. [file elife-96937-supp3.docx]
