# Supplementary material for: Blood metabolomic profiling reveals new targets in the management of psychological symptoms associated with severe alcohol use disorder
Source: eLife. 2024 Nov 29;13:RP96937. doi: 10.7554/eLife.96937 (PMC11606602; doi:10.7554/eLife.96937)
Supplement: MDAR checklist [file elife-96937-mdarchecklist1.docx]

eLife Version of Record checklist

Please refer to this checklist when you are uploading your Version of Record.

Article file

| **Title** is approx. 120 characters or fewer. It is one clear and concise sentence and does not include punctuation or multiple parts*. The biological system is indicated in the title and/or abstract.  *For articles in **Medicine or Epidemiology and Global Health,** two-part titles are allowed, and the study design is in the title. |  |
| --- | --- |
| **Authors:** all authors are listed with the correct names, in the desired order with all affiliations relevant to the study. |  |
| **Abstract:** normally a single 150–200 word paragraph*  If the biological system not mentioned in the title, it is included early in the abstract.  ***For Medical submissions:** up to 250 words. Subheadings: Background; Methods; Results; Conclusions; Funding; Clinical trial number (for submissions reporting results of a trial only). |  |
| **Article file:** uploaded as a .docx or .tex file.  **LaTex:** for .tex files, all .cls, .sty, and .bib files are uploaded as Latex Support files and a clean, compiled PDF is uploaded as a Related Manuscript file. |  |
| **Tables** are editable and included within the article file so that they can be typeset during the production process.  Where possible, bold and/or italics are used instead of colours to highlight cells or data. |  |
| **Key Resources Table:** follows the eLife [template](https://elife-cdn.s3.amazonaws.com/author-guide/elifeKRTtemplatev2.docx). The Key Resources Table is incorporated within the article file at the very beginning of the Materials and Methods section. | NA |
| **Appendices:** if applicable, included at the end of the main article file or uploaded in an editable format as a second article file. | NA |

Figure, figure supplement and video files

| **Figures:** each main figure is provided as an individual file in one of the accepted formats (PDF, EPS, AI, JPEG, PNG or 16 bit TIF).  No figure numbers, titles, or author names appear in the figure files (e.g., “Figure 1, Holmes et al.” is not permitted). |  |
| --- | --- |
| **Appendix figures:** provided as an individual file in one of the accepted formats (PDF, EPS, AI, JPEG, PNG or 16 bit TIF) and labelled in the format “Appendix 1–figure 1”, “Appendix 1–figure 2”, “Appendix 2–figure 1” etc. | NA |
| **Figure supplements:** each figure supplement is provided as an individual file in one of the accepted formats (PDF, EPS, AI, JPEG, PNG or 16 bit TIF) and fits on one page. | NA |
| Figure supplements are **linked to a main figure** and labelled in the format “Figure 1–figure supplement 1”, “Figure 1–figure supplement 2”, “Figure 2–figure supplement 1” etc. | NA |
| **Principles of** **Colour Universal Design** (Masataka, Okabe and Kei Ito, J*Fly) used when preparing figures, to ensure maximum accessibility for all types of colour vision. |  |
| **Rich Media/Videos:** uploaded in one of the accepted formats (AVI, WMV, MOV, MP4, or H264). Rich media files can be independent or linked to main figure (e.g. Animation 1, Video 1, Figure 1–video 1) . | NA |
| **Figure permissions:** the legend for any adapted, redrawn, or reproduced figure includes: the reference of the original publication (with original figure number); the licence of the original publication; and that the copyright holder has granted permissions to publish under a CC BY 4.0 licence. | NA |

Additional submission files

| **MDAR checklist**  A completed version of the MDAR checklist (PDF; Word) has been uploaded, which is suitable for publication in the event of acceptance. Any data information included in this file is also included in the Data Availability Statement. |  |
| --- | --- |
| **Source data files**: **All data generated and analysed should be shared in accordance with our** [**Data availability policy**](https://submit.elifesciences.org/html/elife_author_instructions.html#policies)**.**  Source data files under 100MB have been uploaded and are labelled correctly.  – Where linked to a figure, figure supplement or table, source data files follow this naming convention: ‘Figure 1–Source Data 1’, ‘Figure 1–Figure Supplement 1–Source Data 1’, 'Table 1–Source Data 1'. If the data is linked to more than one display item (e.g. Figure 2 and Figure 3), only link it to **one** of these (e.g. ‘Figure 2–Source Data 1’).  – Where linked to the manuscript as a whole, source data files are labelled ‘Source Data 1’, ‘Source Data 2’ etc.  (Datasets which include many files larger than 100MB need to be deposited to an external repository. See our [Data availability policy](https://submit.elifesciences.org/html/elife_author_instructions.html#policies) for more information.)  Each source data file has a title (and an optional legend) provided within the article file.  **If gels or blots are depicted**, source data files are required for the relevant figure(s) and figure supplement(s). Zipped folder(s) containing **both** the original files of the full raw unedited gels or blots **and** the uncropped gels or blots with relevant bands clearly labelled have been uploaded and the files have been labelled correctly (see above). | NA  NA  NA  NA |
| **Source code files: Software and code should be shared in accordance with our** [**Software sharing policy**](https://submit.elifesciences.org/html/elife_author_instructions.html#policies)**.**  Source code files are labelled correctly.  – Where linked to a figure, figure supplement or table, source code files follow this naming convention: ‘Figure 1–Source Code 1’, ‘Figure 1–Figure Supplement 1–Source Code 1’, 'Table 1–Source Code 1'. If the code is linked to more than one display item (e.g. Figure 2 and Figure 3), the label only links it to **one** of these (e.g. ‘Figure 2–Source Code 1’).  – Where linked to the manuscript as a whole, source code files are labelled ‘Source Code 1’, ‘Source Code 2’ etc.  Each source code file has a title (and an optional legend) provided within the article file. | NA  NA |
| **Supplementary files: Supplementary files are reserved for information not central to the narrative which falls outside of the other formats specified above, such as long lists of strains or plasmids.**  Supplementary files are uploaded individually in the most useful format (i.e. which allows for re-use of the information contained in the file). Each Supplementary File is uploaded individually using the ‘Supplementary File’ format and is correctly labelled “Supplementary File 1”, “Supplementary File 2”, etc. |  |
| **Answers for the *eLife* Digest [recommended]:** if interested, an ‘Answers for the eLife Digest’ file has been uploaded, as described in [this blog post](https://elifesciences.org/inside-elife/85518309/plain-language-summaries-how-to-write-an-elife-digest). | NA |
| **Potential Striking Image [recommended]:** is at least 1800 x 900 pixels, provided in png, tiff or jpeg formats. Image is a single panel and does not include labels or text. All Striking Images must be available to use under the terms of a [Creative Commons Attribution License](http://creativecommons.org/licenses/by/4.0/). Please see [here](https://elifesciences.org/archive/2017?_ga=2.185507817.1118761911.1634492834-644861666.1626705699), or look at [our social media channels](https://twitter.com/eLife/media) for more ideas about the types of images we look for. | NA |

Submission information

| **Title and Abstract** match the title and abstract provided in the Article file and follow the same guidelines. |  |
| --- | --- |
| **Impact Statement:** a single, complete sentence under 40 words that does not begin with phrases like: “We show...”, “This study…” or “Our work…” |  |
| **Author details** are complete for all authors (name, current affiliation, current email address, city, country and competing interest) and authors are listed in the same order as the Article File. |  |
| **Author contributions**: at least one box checked for every author. |  |
| **Permissions**: state whether any previously published material have been adapted or reproduced.  If so, the reference of the original publication (with original figure number); the licence of the original publication; and whether the copyright holder has granted permissions to publish under a CC BY 4.0 licence have been stated. | NA |
| **Data availability statement** describes where and how the data and code for this study has been made publicly available, including DOI or accession numbers. | NA |
| **Animal ethics statement** states the institutional review board or ethics committee that approved the study and/or the guidelines that have been followed. Statement includes any protocol or reference numbers associated with the ethical approval of your work.  **Human ethics statement** explains that informed consent, and consent to publish, was obtained, or if this was not necessary, please state this and explain the reasons. If necessary, the consent process can be described in depth in the Materials and Methods. The specific ethical approval obtained and/or guidelines that were followed, including the name of the institutional review board or ethics committee that approved the study, where applicable, along with any relevant approval identifier are included. | NA |
| **Funding information** is complete, with grant numbers where possible, and the authors associated with the specific funding sources. |  |
| **Cell line** source has been stated i.e. where the cell lines were obtained from.  **Cell identity** has been **authenticated** and include details of the authentication method used (such as STR profiling). Alternatively, the identity was verified by other means if cell line authentication is not commercially available.  Confirm that the **mycoplasma contamination testing** is negative. | NA |
