# Supplementary material for: Blood metabolomic profiling reveals new targets in the management of psychological symptoms associated with severe alcohol use disorder
Source: eLife. 2024 Nov 29;13:RP96937. doi: 10.7554/eLife.96937 (PMC11606602; doi:10.7554/eLife.96937)
Supplement: Supplementary file 2. [file elife-96937-supp2.pdf]

Column	ESI	Retention time (min)	m/z	Adduct.type	Molecular formula	Metabolite identification	MS/MS fragments (%)	ID level	HDMB ID	Present in Frontal cortex[1]	Present in Cerebrospinal fluid[1]	Frontal cortex metabolites, Alcohol vs. Control, Welch's t-test: t_test_P	t_test_P_FDR
Reversed-phase	Positive	7.97	225.19568	[M+H]+	C13H24N2O	**1,3-Dicyclohexylurea**	**225.196 (100), 100.112 (36)**	2	HMDB0244166				
Reversed-phase	Positive	11,66	338,34137	[M+H]+	C22H43NO	**13-Docosenamide**	**338.342 (100), 83.086 (71), 69.071 (69), 97.102 (64), 57.071 (50)**	2	HMDB0244507	x	x	4,85E-01	7,33E-01
HILIC	Positive	6.43	170.09248	[M+H]+	C7H11N3O2	**1-Methylhistidine**	**124.086 (100), 170.092 (31), 109.078 (10), 83.057 (5), 79.053 (4)**	1	HMDB0000001	x	x	1.35E-02	1.28E-01
HILIC	Positive	2.37	137.07065	[M]+	C7H9N2O	**1-Methylnicotinamide**	**137.071 (100), 94.066 (51), 78.034 (14), 120.045 (9), 67.055 (7), 87.494 (4)**	1	HMDB0000699	x	x	5.91E-01	8.15E-01
Reversed-phase	Positive	1,67	153,0659	[M+H]+	C7H8N2O2	**1-methyl-pyridone-carboxamide**	**153.065 (100), 106.964 (19), 104.948 (11)**	2	n/a				
Reversed-phase	Positive	6,13	167,99364	[M+H]+	C7H5NS2	**2-Mercaptobenzothiazole**	**Pos: 167.994 (100), 108.96 (6)**<br>**Neg: 165.98 (100), 134.008 (10)**	2	HMDB0030524				
Reversed-phase	Positive	6,46	211,09691	[M+H]+	C11H14O4	**3-(?,?-dimethoxyphenyl)propanoic acid**	**165.091 (100), 151.075 (95), 193.087 (80)**	3	n/a				
Reversed-phase	Negative	8.82	267.12415	[M-H]-	C14H20O5	**3-CMPFP**	**223.134 (100), 179.145 (46), 267.124 (23)**	2	HMDB0061643				
Reversed-phase	Negative	6.50	159.10274	[M-H]-	C8H16O3	**3-Hydroxyoctanoic acid**	**59.013 (100), 159.103 (15), 130.984 (12)**	2	HMDB0001954				
HILIC	Negative	0.79	117.05537	[M-H]-	C5H10O3	**3-Hydroxyvaleric acid**	**71.05 (100), 117.055 (44), 32.031 (10), 36.105 (8), 51.807 (5), 72.996 (5)**	2	HMDB0000531	x	x	6.35E-06	4.90E-04
Reversed-phase	Positive	5,29	176,07066	[M+H]+	C10H9NO2	**3-Indoleacetic acid**	**130.065 (100), 176.071 (11)**	1	HMDB0000197				
Reversed-phase	Positive	6.03	190.08633	[M+H]+	C11H11NO2	**3-Indolepropionic acid**	**130.065 (100), 172.075 (21), 190.086 (15)**	2	HMDB0002302				
HILIC	Positive	7.01	170.09267	[M+H]+	C7H11N3O2	**3-Methylhistidine**	**170.093 (100), 96.069 (40), 126.103 (32), 109.077 (30), 97.077 (14), 125.072 (4)**	2	HMDB0000479	x	x	8.50E-02	3.55E-01
Reversed-phase	Negative	5.09	201.02304	[M-H]-	C8H10O4S	**4-Ethylphenylsulfate**	**121.065 (100), 201.023 (92)**	2	HMDB0062551				
Reversed-phase	Positive	0.67	148.09689	[M+H]+	C6H13NO3	**4-Hydroxyisoleucine**	**74.024 (100), 102.092 (49)**	2	n/a				
Reversed-phase	Positive	1.72	184.06053	[M+H]+	C8H9NO4	**4-Pyridoxic acid**	**166.05 (100), 148.039 (27), 184.063 (19)**	2	HMDB0000017				
HILIC	Positive	3,57	146,11778	[M+H]+	C7H15NO2	**4-Trimethylammoniobutanoic acid**	**146.118 (100), 87.044 (61), 60.081 (30), 43.018 (6)**	1	HMDB0001161	x	x	1,43E-06	1,47E-04
HILIC	Positive	2.23	160.13298	[M+H]+	C8H17NO2	**5-AVAB**	**55.054 (100), 83.05 (56), 60.082 (51), 101.06 (39), 160.136 (38), 59.05 (37), 43.017 (14), 58.065 ([illegible]**	1	HMDB0240732	x	x	1.26E-01	4.27E-01
Reversed-phase	Positive	9,95	431,31561	[M+H]+	C27H42O4	**7a-hydroxy-3-oxo-4-cholestenoic acid**	**Pos: 395.292 (100), 97.065 (92), 431.316 (73)**<br>**Neg: 429.305 (100), 411.289 (24)**	1	HMDB0012458				
Reversed-phase	Positive	1,75	232,15427	[M+H]+	C11H21NO4	**AC 04:0 (Butanoylcarnitine)**	**85.029 (100), 232.156 (38)**	1	HMDB0002013	x	x	2,92E-02	2,02E-01
HILIC	Positive	1.39	246.1714	[M+H]+	C12H23NO4	**AC 05:0 (Isovalerylcarnitine)**	**85.029 (100), 246.173 (71), 60.082 (34), 187.095 (30), 144.106 (17), 44.996 (12), 57.071 (11)**	1	HMDB0013128	x	x	5.98E-02	2.94E-01
Reversed-phase	Positive	4,21	260,18552	[M]+	C13H26NO4	**AC 06:0 (Hexanoylcarnitine)**	**85.029 (100), 260.186 (44), 260.194 (20)**	1	HMDB0000756		x		
Reversed-phase	Positive	6.01	288,21683	[M+H]+	C15H29NO4	**AC 08:0 (Octanoylcarnitine)**	**85.029 (100), 288.217 (44)**	1	HMDB0000791				
Reversed-phase	Positive	5.20	286.20117	[M+H]+	C15H27NO4	**AC 08:1 (Octenoyl-L-carnitine)**	**85.029 (100), 286.199 (48)**	1	HMDB0240723				
Reversed-phase	Positive	6.37	302.23239	[M+H]+	C16H31NO4	**AC 09:0 (Nonanoylcarnitine)**	**85.029 (100), 302.233 (52)**	3	HMDB0013288				
Reversed-phase	Positive	7.27	316.24814	[M+H]+	C17H33NO4	**AC 10:0 (Decanoylcarnitine)**	**85.029 (100), 316.25 (47)**	1	HMDB0000651				
Reversed-phase	Positive	6.95	314.23242	[M+H]+	C17H31NO4	**AC 10:1 (Decenoylcarnitine)**	**85.029 (100), 314.233 (43)**	2	n/a				
Reversed-phase	Positive	6.21	312.2168	[M+H]+	C17H29NO4	**AC 10:2 (Decadienoylcarnitine)**	**85.029 (100), 312.218 (50)**	2	n/a				
Reversed-phase	Positive	5,95	310,20114	[M+H]+	C17H27NO4	**AC 10:3 (Decatrienoylcarnitine)**	**85.029 (100), 310.203 (34)**	2	n/a				
HILIC	Positive	0.82	328.24872	[M+H]+	C18H33NO4	**AC 11:1 (Undecenoylcarnitine)**	**328.249 (100), 85.028 (20), 269.177 (13), 133.081 (4)**	3	n/a				
Reversed-phase	Positive	8.15	344.27945	[M+H]+	C19H38NO4	**AC 12:0 (Dodecanoylcarnitine)**	**85.029 (100), 344.281 (49)**	1	HMDB0002250	x		5.61E-01	7.82E-01
Reversed-phase	Positive	7,74	342,26379	[M+H]+	C19H35NO4	**AC 12:1 (Dodecenoylcarnitine)**	**85.029 (100), 342.266 (46)**	1	n/a				
Reversed-phase	Positive	8.12	356.27933	[M+H]+	C20H37NO4	**AC 13:1 (Tridecenoylcarnitine)**	**85.029 (100), 356.281 (61)**	3	n/a				
Reversed-phase	Positive	8.81	372.3107	[M]+	C21H42NO4	**AC 14:0 (Tetradecanoyl-L-carnitine)**	**85.029 (100), 372.31 (45)**	1	HMDB0005066	x		8.24E-01	9.26E-01
Reversed-phase	Positive	8,49	370,2951	[M]+	C21H40NO4	**AC 14:1 (Tetradecenoylcarnitine)**	**85.029 (100), 370.293 (51)**	1	n/a				
Reversed-phase	Positive	8,11	368,27948	[M+H]+	C21H37NO4	**AC 14:2 (Tetradecadienoylcarnitine)**	**85.029 (100), 368.282 (55)**	2	n/a				
Reversed-phase	Positive	4.85	304.2117	[M+H]+	C15H29NO5	**AC 15:OH (Hydroxyoctanoylcarnitine)**	**85.029 (100), 304.208 (66)**	3	n/a				
Reversed-phase	Positive	9,32	400,34164	[M]+	C23H46NO4	**AC 16:0 (Palmitoylcarnitine)**	**85.029 (100), 400.343 (53), 400.358 (26)**	1	HMDB0000222	x	x	4,85E-01	7,33E-01
Reversed-phase	Positive	8.99	398.32642	[M]+	C23H43NO4	**AC 16:1 (O-Palmitoleoylcarnitine)**	**85.029 (100), 398.329 (61), 398.314 (35)**	2	HMDB0240782	x		4.79E-01	7.29E-01
Reversed-phase	Positive	6.31	332.24298	[M+H]+	C17H33NO5	**AC 17:OH (Hydroxydecanoylcarnitine)**	**85.029 (100), 332.245 (67), 288.036 (27)**	3	n/a				
Reversed-phase	Positive	9.44	426.35703	[M]+	C25H48NO4	**AC 18:1 (Oleylcarnitine)**	**85.029 (100), 426.361 (52)**	1	HMDB0005065	x	x	5.43E-01	7.72E-01
Reversed-phase	Positive	9.17	424.34195	[M]+	C25H46NO4	**AC 18:2 (Linoleylcarnitine)**	**85.029 (100), 424.34 (58)**	1	HMDB0006469	x	x	6,73E-01	8.48E-01
HILIC	Positive	3.20	204.1239	[M+H]+	C9H17NO4	**Acetylcarnitine**	**85.028 (100), 43.018 (37), 57.033 (28), 58.064 (18), 60.08 (16), 84.08 (11), 41.038 (5), 45.057 (4)**	1	HMDB0240773	x	x	5.41E-02	2.79E-01
Reversed-phase	Negative	7.16	369.17441	[M-H]-	C19H30O5S	**Androsterone sulfate isomer 1**	**369.171 (100)**	2	HMDB0002759				
Reversed-phase	Negative	7.85	369.17413	[M-H]-	C19H30O5S	**Androsterone sulfate isomer 2**	**369.173 (100), 96.96 (30)**	2	HMDB0002759		x		
Reversed-phase	Negative	8.99	369.17456	[M-H]-	C19H30O5S	**Androsterone sulfate isomer 3**	**369.173 (100), 96.96 (34)**	2	HMDB0002759				
Reversed-phase	Negative	9,47	512,2688	[M-H]-	C26H43NO7S	**Bile acid_C26H43NO7S**	**432.316 (100), 512.272 (70), 74.024 (62)**	3	n/a				
Reversed-phase	Negative	8.94	528.26453	[M-H]-	C26H43NO8S	**Bile acid C26H43NO8S isomer 1**	**448.304 (100), 528.263 (57), 74.024 (43)**	3	n/a				
Reversed-phase	Negative	7.56	528.2644	[M-H]-	C26H43NO8S	**Bile acid C26H43NO8S isomer 2**	**448.305 (100), 74.024 (77), 528.264 (76)**	3	n/a				
Reversed-phase	Negative	8.76	528.26453	[M-H]-	C26H43NO8S	**Bile acid C26H43NO8S isomer 3**	**448.304 (100), 74.024 (65), 528.261 (61)**	3	n/a				
Reversed-phase	Positive	7,16	585,27087	[M+H]+	C33H36N4O6	**Bilirubin isomer 1**	**Pos: 299.138 (100), 285.123 (72), 585.276 (62), 568.237 (50)**<br>**Neg: 227.12 (100), 299.142 (69), 253.135 (61), 239.119 (57)**	2	HMDB0000054				
[illegible]	[illegible]	[illegible]	[illegible]	[illegible]	[illegible]	[illegible]	[illegible]	[illegible]	[illegible]	[illegible]	[illegible]	[illegible]	[illegible]
Reversed-phase	Positive	9,08	451,22803	[M+Na]+		**Unknown**	**177.032 (100), 74.007 (97), 451.227 (57)**	4	n/a				
Reversed-phase	Positive	8,73	414,30035	[M+H-H2O]+		**Unknown**	**414.293 (100), 339.265 (62), 76.04 (60), 85.029 (57)**	4	n/a				
Reversed-phase	Negative	10,64	339,23309	[M-H]-		**Unknown**	**163.113 (100), 339.236 (52)**	4	n/a				
Reversed-phase	Positive	10,26	311,633	[M+H]+		**Unknown**	**184.075 (100), 311.632 (86), 86.097 (71), 256.003 (55)**	4	n/a				
Reversed-phase	Positive	10,27	590,32141	[M+Na]+		**Unknown**	**104.108 (100), 531.253 (74)**	4	n/a				
Reversed-phase	Positive	11,24	397,27139	[M+K]+		**Unknown**	**181.554 (100), 199.109 (86), 95.177 (83), 228.922 (83), 151.583 (83), 130.956 (81), 91.426 (80), 1[illegible]**	4	n/a				
Reversed-phase	Positive	11,22	485,33893	[M+K]+		**Unknown**	**485.34 (100), 112.896 (77), 72.011 (29), 80.913 (28)**	4	n/a				
HILIC	Positive	8,12	125,98598	[M+H]+		**Unknown**	**84.96 (100), 50.078 (12), 91.905 (9), 35.289 (8), 113.539 (6), 124.236 (4)**	4	n/a				
Reversed-phase	Positive	0,52	140,06805	[M+Na]+		**Unknown**	**140.069 (100), 74.097 (37)**	4	n/a				
Reversed-phase	Positive	4,17	406,16403	[M+H]+		**Unknown**	**406.165 (100), 210.088 (83)**	4	n/a				
Reversed-phase	Negative	9,67	498,29019	[M-H]-		**Unknown**	**498.291 (100),**	4	n/a				
Reversed-phase	Positive	3,85	264,9328	[M+K]+		**Unknown**	**142.574 (100), 158.809 (98), 71.038 (98), 89.094 (96)**	4	n/a				
Reversed-phase	Positive	10,35	454,29276	[M+H]+		**Unknown**	**313.273 (100), 57.071 (13), 71.086 (12)**	4	n/a				
Reversed-phase	Negative	9,64	250,14529	[M-H]-		**Unknown**	**250.144 (100), 148.406 (4)**	4	n/a				
Reversed-phase	Negative	0,73	207,83714	[M-H]-		**Unknown**	**103.921 (100), 87.925 (70)**	4	n/a				
Reversed-phase	Positive	10,27	606,29572	[M+K]+		**Unknown**	**104.108 (100), 547.226 (30), 86.097 (24)**	4	n/a				
Reversed-phase	Negative	0,52	201,80286	[M-H]-		**Unknown**	**164.836 (100), 201.802 (78), 166.833 (31)**	4	n/a				
Reversed-phase	Positive	8,26	302,21478	[M+H]+		**Unknown**	**165.163 (100), 189.165 (61), 95.086 (51), 109.102 (48)**	4	n/a				
Reversed-phase	Negative	10,96	379,15872	[M-H]-		**Unknown**	**116.928 (100), 99.926 (85), 379.157 (50)**	4	n/a				
Reversed-phase	Positive	10,48	568,33716	[M+Na]+		**Unknown**	**104.108 (100), 509.264 (96)**	4	n/a				
Reversed-phase	Negative	8,72	317,05875	[M-H]-		**Unknown**	**231.023 (100), 230.986 (5), 180.991 (5), 316.986 (5)**	4	n/a				
Reversed-phase	Negative	8,89	514,28491	[M-H]-		**Unknown**	**514.29 (100), 112.986 (6)**	4	n/a				
Reversed-phase	Positive	5,89	171,14954	[M+H]+		**Unknown**	**171.15 (100), 89.071 (61) 72.045 (36)**	4	n/a				
Reversed-phase	Negative	0,62	251,82265	[M+FA-H]-		**Unknown**	**99.926 (100), 207.833 (18)**	4	n/a				
Reversed-phase	Positive	5,89	193,13106	[M+Na]+		**Unknown**	**193.129 (100), 132.959 (58), 116.964 (37)**	4	n/a				
Reversed-phase	Positive	8,31	241,17316	[M+H-2H2O]+		**Unknown**	**241.175 (100), 100.112 (67)**	4	n/a				
Reversed-phase	Positive	8,01	397,14124	[M+K]+		**Unknown**	**306.21 (100), 331.042 (95), 55.028 (89), 74.702 (84), 88.425 (84)**	4	n/a				
Reversed-phase	Positive	10,25	172,13297	[M+H]+		**Unknown**	**109.102 (100), 67.055 (50), 96.963 (32)**	4	n/a				
Reversed-phase	Positive	3,88	148,03932	[M+H-H2]+		**Unknown**	**148.039 (100), 106.086 (18), 148.034 (11)**	4	n/a				
Reversed-phase	Negative	8,11	288,61978	[M-H]-		**Unknown**	**288.619 (100), 96.96 (33), 289.117 (21)**	4	n/a				
Reversed-phase	Negative	0,52	199,80569	[M+Cl]-		**Unknown**	**199.805 (100), 162.839 (97), 164.836 (86)**	4	n/a				
Reversed-phase	Positive	12,98	422,76212	[M+CH3OH+H]2+		**Unknown**	**367.192 (100), 86.097 (28), 368.195 (16)**	4	n/a				
Reversed-phase	Negative	9,39	397,22333	[M-H]-		**Unknown**	**75.008 (100), 280.983 (85), 85.029 (66), 397.224 (64)**	4	n/a				
Reversed-phase	Positive	9,37	259,10892	[M+H]+		**Unknown**	**259.11 (100), 231.807 (59), 205.965 (51), 76.362 (43)**	4	n/a				
Reversed-phase	Negative	4,61	199,00731	[M-H]-		**Unknown**	**119.051 (100), 199.009 (30**	4	n/a				
Reversed-phase	Negative	14,26	750,54565	[M-H]-		**Unknown**	**303.233 (100), 78.958 (10), 140.012 (8)**	4	n/a				
HILIC	Positive	4,42	229,15535	[M-H]-		**Unknown**	**229.156 (100)**	4	n/a				
Reversed-phase	Negative	8,43	187,13405	[M-H]-		**Unknown**	**141.128 (100),187.133 (80)**	4	n/a				

Metabolite identification	Cerebrospinal fluid metabolites, Alcohol vs. Control, Welch's t-test: t_test_P	t_test_P_FDR	AUD T1 vs. Control, sPLS-DA VIP: Component 1	Component 2	AUD T1 vs. AUD T2, sPLS-DA VIP: Component 1	Component 2	AUD T1 vs. Control, Plasma metabolites, Welch's t-test: t_test_P	t_test_P_FDR	Cohen_d	AUD T2 vs. Control, Plasma metabolites, Welch's t-test: <	t_test_P_FDR	Cohen_d	AUD T1 vs. AUD T2, Plasma metabolites, Paired t-test: t_test_P	t_test_P_FDR	Cohen_d	AUD T1 MS/MS features and Alcohol intake, Spearman: Correlation_p	AUD T1 MS/MS features and Obsession (OCDS), Spearman: Correlation_p	AUD T1 MS/MS features and Compulsion (OCDS), Spearman: Correlation_p	AUD T1 MS/MS features and Craving (OCDS - Total Score), Spearman: Correlation_p	AUD T1 MS/MS features and Anxiety state (STAI), Spearman: Correlation_p	AUD T1 MS/MS features and Depression (BDI), Spearman: Correlation_p	AUD T1 MS/MS features and Alcohol intake, Spearman: Correlation_R	AUD T1 MS/MS features and Obsession (OCDS), Spearman: Correlation_R	AUD T1 MS/MS features and Compulsion (OCDS), Spearman: Correlation_R	AUD T1 MS/MS features and Craving (OCDS - Total Score), Spearman: Correlation_R	AUD T1 MS/MS features and Anxiety state (STAI), Spearman: Correlation_R	AUD T1 MS/MS features and Depression (BDI), Spearman: Correlation_R
1,3-Dicyclohexylurea			1.419205014	1.342093579	0.148569713	0.412290533	8.30E-03	2.79E-02	-0.576133597	1.06E-05	3.33E-04	-1.321054313	8.52E-01	9.37E-01	0.042507482	6.32E-03	7.95E-01	5.74E-01	8.98E-01	4.61E-01	8.48E-01	-0.28121328	-0.030536839	0.06641939	0.015095471	0.078258596	0.020611009
13-Docosenamide	4,10E-02	2,14E-01	0,001376214	0,0477934	0,198086872	0,235731974	9,98E-01	9,99E-01	-0,000556385	6,18E-01	7,40E-01	-0,128623528	7,49E-01	8,97E-01	-0,056685093	1,87E-01	5,22E-01	6,27E-01	2,95E-01	1,52E-01	4,77E-01	0,138047578	-0,075135756	-0,057480901	-0,123403255	-0,151250553	-0,076345903
1-Methylhistidine	1.73E-01	4.63E-01	0.112994503	0.294321836	0.518031229	0.496017782	8.19E-01	8.79E-01	0.046273805	3.05E-01	4.57E-01	0.24291459	5.35E-01	7.76E-01	0.148598303	3.17E-01	9.60E-01	7.63E-01	9.96E-01	2.04E-01	9.79E-02	-0.104966296	-0.005881803	-0.035664422	0.000519	-0.13478578	-0.176539254
1-Methylnicotinamide	4.94E-01	7.71E-01	0.435663089	0.560524823	0.717975636	0.614366236	3.09E-01	4.52E-01	0.1907745	5.03E-01	6.41E-01	-0.149449461	3.56E-01	6.54E-01	-0.206490541	8.41E-02	5.42E-01	3.56E-01	3.09E-01	4.90E-01	1.15E-01	-0.180111689	-0.071595253	-0.108754104	-0.119903715	-0.073318632	-0.168150062
1-methyl-pyridone-carboxamide			1.1158179	0.991908267	0.017182972	0.325015176	1.96E-02	5.75E-02	-0.474044615	2.94E-02	9.95E-02	-0.565762328	9.83E-01	9.93E-01	-0.004915123	1.72E-01	1.63E-01	5.52E-03	3.65E-02	1.53E-02	1.82E-02	-0.142956799	-0.162691826	-0.31953233	-0.243588713	-0.253659201	-0.249904225
2-Mercaptobenzothiazole			1,980662636	1,791770967	0,072509349	0,142899477	1,17E-03	5,27E-03	-0,772650371	3,87E-04	4,60E-03	-1,19881743	9,20E-01	9,67E-01	0,020742097	5,12E-01	3,07E-01	7,64E-01	4,64E-01	8,67E-01	4,95E-01	0,068811156	0,119549083	-0,035455509	-0,086509804	-0,017839645	-0,073322387
[illegible]	[illegible]	[illegible]	[illegible]	[illegible]	[illegible]	[illegible]	[illegible]	[illegible]	[illegible]	[illegible]	[illegible]	[illegible]	[illegible]	[illegible]	[illegible]	[illegible]	[illegible]	[illegible]	[illegible]	[illegible]	[illegible]	[illegible]	[illegible]	[illegible]	[illegible]	[illegible]	[illegible]

[1] Detailed peak properties availavable at Kärkkäinen O, Kokla M, Lehtonen M, Auriola S, Martiskainen M, Tiihonen J, et al. Changes in the metabolic profile of human male postmortem frontal cortex and cerebrospinal fluid samples associated with heavy alcohol use. Addiction Biology. 2021;26:e13035.
